# Machine learning approaches for the prediction of bone mineral density by using genomic and phenotypic data of 5130 older men

**DOI:** 10.1038/s41598-021-83828-3

**Published:** 2021-02-24

**Authors:** Qing Wu, Fatma Nasoz, Jongyun Jung, Bibek Bhattarai, Mira V. Han, Robert A. Greenes, Kenneth G. Saag

**Affiliations:** 1 Nevada Institute of Personalized Medicine, University of Nevada Las Vegas, 4505 Maryland Parkway, Las Vegas, NV 89154-4009 USA; 2 Department of Epidemiology and Biostatistics, School of Public Health, University of Nevada, Las Vegas, NV USA; 3 Department of Computer Science, University of Nevada, Las Vegas, NV USA; 4 The Lincy Institute, University of Nevada, Las Vegas, NV USA; 5 School of Life Sciences, University of Nevada, Las Vegas, NV USA; 6 College of Health Solutions, Arizona State University, Phoenix, AZ USA; 7 Department of Health Science Research, Mayo Clinic, Scottsdale, AZ USA; 8 Department of Medicine, Division of Clinical Immunology and Rheumatology, the University of Alabama at Birmingham, Birmingham, AL USA

**Keywords:** Environmental sciences, Medical research

## Abstract

The study aimed to utilize machine learning (ML) approaches and genomic data to develop a prediction model for bone mineral density (BMD) and identify the best modeling approach for BMD prediction. The genomic and phenotypic data of Osteoporotic Fractures in Men Study (n = 5130) was analyzed. Genetic risk score (GRS) was calculated from 1103 associated SNPs for each participant after a comprehensive genotype imputation. Data were normalized and divided into a training set (80%) and a validation set (20%) for analysis. Random forest, gradient boosting, neural network, and linear regression were used to develop BMD prediction models separately. Ten-fold cross-validation was used for hyper-parameters optimization. Mean square error and mean absolute error were used to assess model performance. When using GRS and phenotypic covariates as the predictors, all ML models’ performance and linear regression in BMD prediction were similar. However, when replacing GRS with the 1103 individual SNPs in the model, ML models performed significantly better than linear regression (with lasso regularization), and the gradient boosting model performed the best. Our study suggested that ML models, especially gradient boosting, can improve BMD prediction in genomic data.

## Introduction

Osteoporosis is characterized by reduced bone mineral density (BMD) and deteriorated bone architecture, leading to increased fracture risk. Osteoporosis and its major complication, osteoporotic fracture, which affects both men and women, cause substantial morbidity and mortality worldwide^1^. Although women have a higher risk of osteoporosis, men suffer greater morbidity and mortality rates following osteoporotic fractures, especially at an advanced age. With populations aging worldwide, osteoporosis is a critical public health problem globally. For example, by 2050, Worldwide hip fracture incidence alone is projected to increase by threefold 10% in men and twofold in women compared to 1990 data^2^. The potentially high cumulative rate of fracture, which often results in excess disability and mortality^3^, has caused an inevitable high social and economic burden associated with bone health.

BMD has been used to define osteoporosis since 1994. The World Health Organization defines osteoporosis as a BMD that lies 2.5 or more standard deviations below the average value for young healthy women^4^. BMD is the single strongest predictor of primary osteoporotic fracture^5^. Each standard deviation decrease in BMD is associated with a 1.5 to threefold increase in fracture risk, depending on the skeletal region measured, type of fracture, and ethnicity of the study population^6^.

BMD is a highly heritable trait. Genetic differences in BMD are well documented^7^. Family and twin studies show BMD variances of 50–85% are attributable to genetic factors^8^. Other studies report BMD heritability estimates of 72–92%^9^. In the past decade, major genome-wide association studies (GWAS) and genome-wide meta-analyses have successfully identified numerous BMD-associated Single Nucleotide Polymorphisms (SNPs) associated with decreased BMD^10^. However, combining these large number of highly significant SNPs, surprisingly, only explained a very small proportion of BMD variance^11^. Such inconsistency may be caused by limitations of the conventional regression approaches employed as these traditional methods lack the flexibility and adequacy of modeling complex interactions and regulations.

Machine learning (ML) focuses on implementing computer algorithms capable of maximizing predictive accuracy from complex data. ML has an excellent capacity to model complex real-world relationships, including variable interactions. ML techniques have been applied in clinical research for disease prediction, and ML has shown much higher accuracy for diagnosis than conventional methods^12^. Gradient boosting, random forest, and neural network are widely used ML approach for modeling complex medical data^12^. However, the performance of these ML models for BMD prediction remains largely unknown, especially with genomic data.

Hence, the current study aims are (1) to develop models using ML algorithms to predict BMD from the data with genomic variants, and (2) to compare these models to determine which ML model performs the best for BMD prediction. We hypothesize that when we utilize the ML models to predict BMD, ML models will perform better than the linear regression model.

## Results

### Baseline characteristics

Table 1 shows the characteristics of participants within the training ($n=4104$) and the test ($n=1026$) datasets. Demographic and clinical variables were not significantly different in training and test datasets. All BMD measurements from the femur neck, total spine, and total hip were normally distributed, with means of 0.78, 0.96, 1.07, and standard deviations 0.13, 0.14, 0.19, respectively. The Pearson correlation between each BMD outcome and demographic/clinical variables ranged from − 0.19 to 0.41 (Table S1). In the study data, 12%, 12%, and 13% of the 1103 SNPs were significantly associated with femoral neck, total hip, and total spine BMD at the significance level $\alpha =0.05$, respectively.

**Table 1:** Baseline characteristics of training and testing dataset.

Variable*	Training dataset (n = 4104)	Test dataset (n = 1026)	*p* Value**
Femoral neck BMD (g/cm^2^)	0.78 ± 0.13	0.79 ± 0.13	0.61
Total hip BMD (g/cm^2^)	0.96 ± 0.14	0.96 ± 0.14	0.55
Total spine BMD (g/cm^2^)	1.07 ± 0.19	1.07 ± 0.19	0.36
Age (year)	73.77 ± 5.93	73.95 ± 5.83	0.78
Height (cm)	174.15 ± 6.81	174.16 ± 6.61	0.69
Weight (kg)	83.28 ± 13.46	82.73 ± 12.73	0.37
Alcohol use (drinks/week)	4.25 ± 6.87	3.98 ± 6.06	0.57
GRS***	31.6 ± 0.44	31.59 ± 0.45	0.81
Impairment of instrumental activities of daily living	0.37 ± 0.88	0.36 ± 0.83	0.79
Walking speed (m/s)	1.07 ± 0.27	1.07 ± 0.27	0.53
Smoking, no. (%)			0.41
No	1535 (37.4%)	406 (39.6%)	
Past	2430 (59.2%)	587 (57.3%)	
Current	139 (3.4%)	32 (3.1%)	
Race, no. (%)			0.11
White	3707 (90.3%)	909 (88.6%)	
African American	141 (3.4%)	40 (3.9%)	
Asian	120 (2.9%)	45 (4.4%)	
Hispanic	87 (2.1%)	24 (2.3%)	
Other	49 (1.2%)	8 (0.8%)	

*Continuous variables were expressed as mean $\pm$ SD, and categorical variables were expressed as number (%).

**p Value were obtained by t-test for continuous variables and chi-square tests for the categorical variable.

***GRS: genetic risk score, which was calculated based on 1103 BMD-related SNPs.

### Model performance

**Figure 1:**
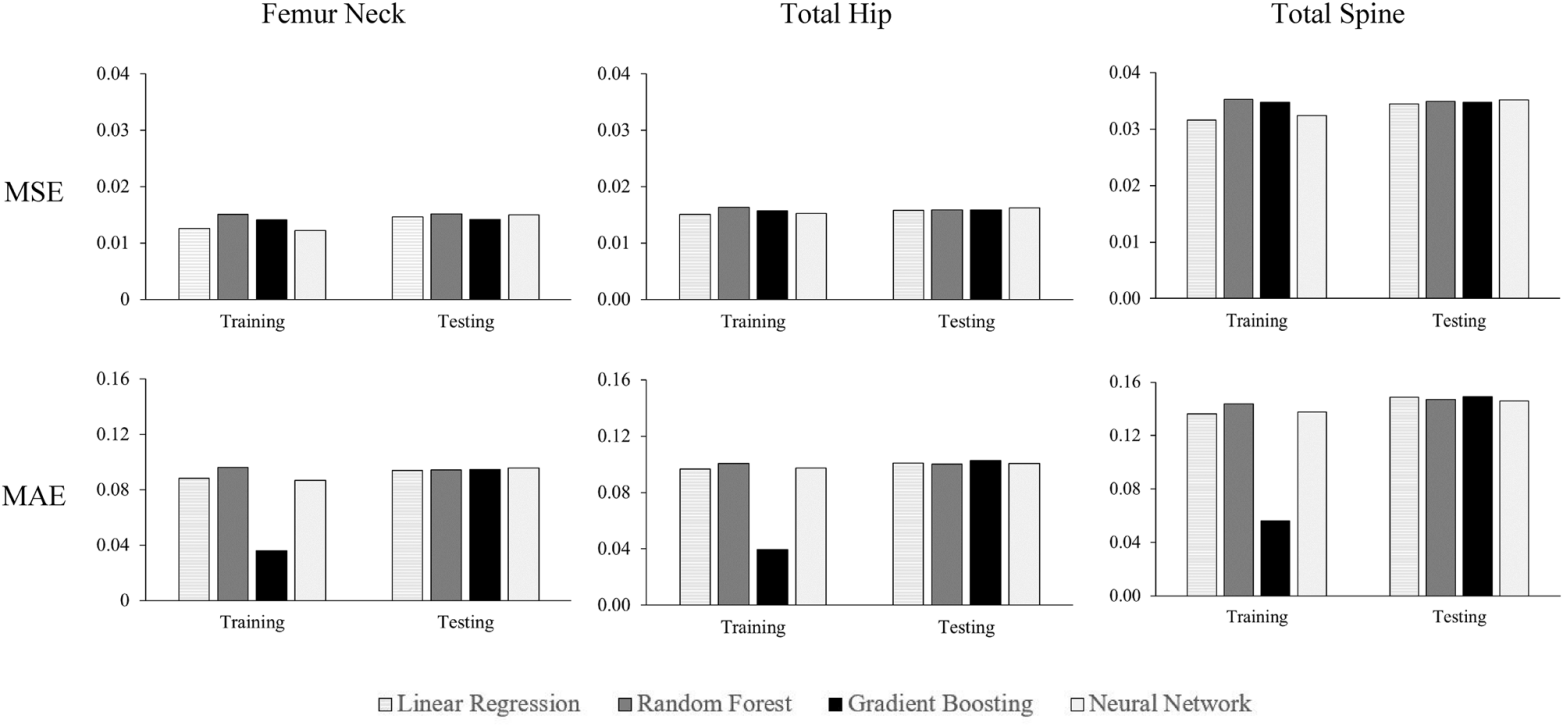
Mean square error (MSE) and mean absolute error (MAE) of different models in predicting various BMD in training (n = 4104) and testing (n = 1026); GRS and phenotypic covariates were used as predictors.

**Figure 2:**
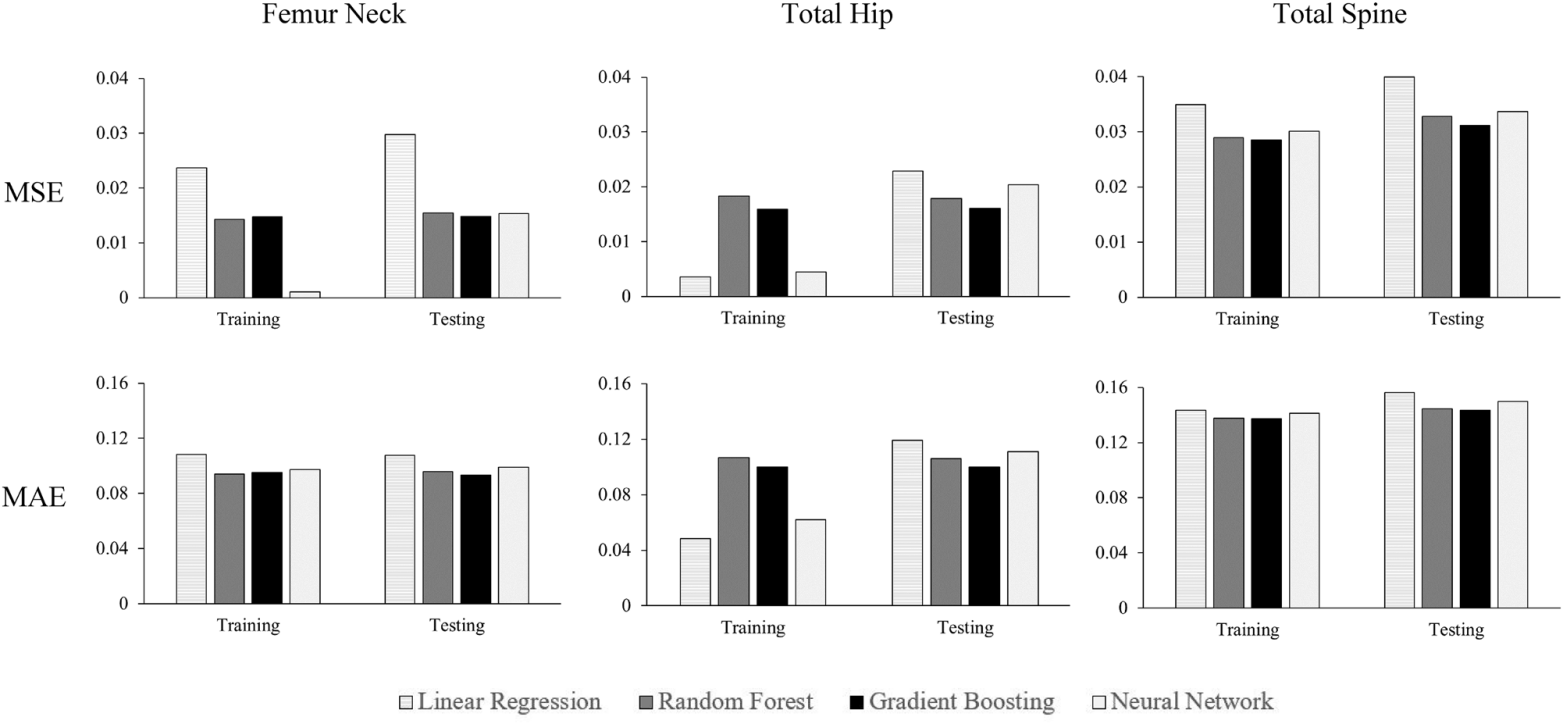
Mean square error (MSE) and mean absolute error (MAE) of different models in predicting various BMD in training (n = 4104) and testing (n = 1026); 1103 individual risk SNPs and phenotypic covariates were used as predictors. Lasso regularization, with the penalized value of 0.01, was applied to linear regression.

Figure 1 shows MSE and MAE for each model with phenotype covariate and GRS as predictors. In the test dataset ($n=1026$), MSE was similar between all models in each BMD outcome, and the same results were observed for MAE. When we replaced GRS with 1103 individual SNPs in the predictors (Fig. 2), ML models had smaller MSE than the linear model (with lasso regularization) in the testing dataset. Similar results were observed with MAE. Overall, the gradient boosting model had the lowest MSE and MAE in the testing dataset.

**Figure 3:**
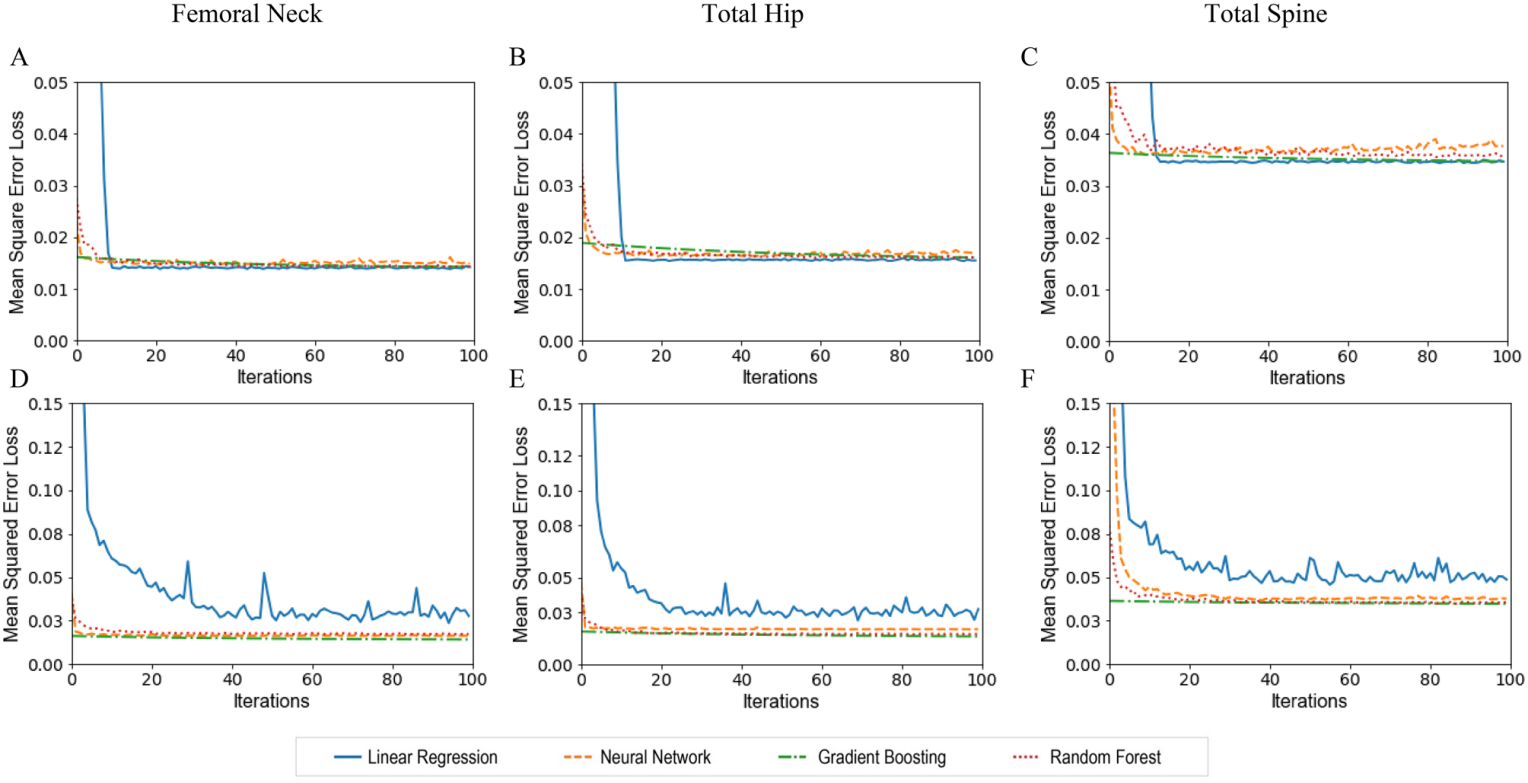
Mean squared error loss of various models with the number of training iterations for BMD prediction in the test dataset ($\mathrm{n}=1026)$. The upper panel shows the performance of each model with phenotype covariates and GRS as predictors in predicting BMD at the femoral neck (**A**), total hip (**B**), and total spine (**C**) in the testing dataset at different BMD sites. The lower panel shows the performance of each model with phenotype covariates and 1103 individual SNPs in predicting BMD at the femoral neck (**D**), total hip (**E**), and total spine (**F**). Lasso regularization with the penalized value of 0.01 was used in the linear regression model for 1103 individual SNPs and phenotype covariates.

Figure 3 shows each model’s performance in the test dataset ($$n=1026)$$. The upper panel in Fig. 3 compared model performance when using phenotype covariates and GRS as predictors. MSE in each model became similar in the test data with increased training iterations. Although the linear regression model had a relatively higher MSE in the first few iterations, in the testing dataset, linear regression and ML models’ performance became nearly identical with increased iterations in BMD measured from all three skeletal regions. All models had the best performance at femur neck BMD (Fig. 3A), followed by total hip BMD (Fig. 3B) and then total spine BMD (Fig. 3C). The lower panel in Fig. 3 compared model performance when using phenotype covariates and 1103 individual SNPs as predictors. MSE in linear regression (with lasso regularization) was much larger than that in other ML models in the testing data. The results were consistent with BMD measured at the three different skeletal regions.

We tested different hyper-parameters in each algorithm (Table S2) in the training data. We found that a relatively small depth of the tree (depth = 5) was observed in random forest and gradient boosting when phenotype covariate and GRS were included as predictors in the model. However, relatively higher for random forest (depth = 20) and gradient boosting (depth = 10) were obtained when phenotype covariate and 1103 individual SNPs were included in the model. The optimal hyper-parameters in the neural network were obtained when the batch size of 20, the first layer with 500 neurons, three hidden layers, and the sigmoid activation function were used.

The coefficient determination (R^2^) of each model was shown in Table S3. For each BMD outcome, the R^2^ in models with 1103 individual risk SNPs and phenotypic covariates ranged from 8 to 18%. The R^2^ in the model with GRS and phenotypic covariates ranged from 6 to 16%. In the testing data, the 1103 individual risk SNPs contribute the variance of each BMD about 1–2% in Femoral Neck and Total Hip BMD and 2–4% in Total Spine BMD, respectively (Table S3). The early stopping iteration in the training dataset for each model was shown in Table S4. Fewer iterations were required for the model with phenotype covariate and GRS as predictors. However, models with phenotype covariate and individual risk SNPs required more iterations for the training, especially in linear regression (with lasso regularization).

To compare the performance between models that uses GRS and phenotype covariates as the predictors, we used the nonparametric Wilcoxon signed-rank test results for multiple comparisons of MSE between models. The results are shown in Table 2. With Bonferroni corrections for multiple comparisons (α = 0.05/6 = 0.0083), none of the comparisons were statistically significant except the comparison between gradient boosting and random forest at femur neck BMD and total spine BMD. However, as shown in Table 3, when using phenotype covariates and 1103 SNPs as the predictors, the difference of MSE in most pairwise comparisons was statistically significant with *p* < 0.0001. The only exceptions were the comparison between the neural network and random forest at femur neck BMD and the comparison between the gradient boosting and random forest at total hip BMD with *p* > 0.05.

**Table 2:** Statistical comparisons of mean square errors in the testing dataset ($n=1026$) between various models when phenotype covariates and GRS were used as the predictors.

	Linear regression	Random forest	Gradient boosting
**Femoral neck BMD**			
Neural network	$> .05$	$> .05$	$> .05$
Gradient boosting	$> .05$	$< .0001$	–
Random forest	$> .05$	–	–
**Total hip BMD**			
Neural network	$> .05$	$> .05$	$> .05$
Gradient boosting	$> .05$	$> .05$	–
Random forest	$> .05$	–	–
**Total spine BMD**			
Neural network	$> .05$	$> .05$	$> .05$
Gradient boosting	$> .05$	$< .01$	–
Random forest	$> .05$	–	–

The Wilcoxon Signed-Rank Test was used to determine all *p* values.

**Table 3:** Statistical comparisons of mean square errors in the testing dataset ($n=1026$) between various models when phenotype covariates and 1103 SNPs were used as the predictors.

	Linear regression*	Random forest	Gradient boosting
**Femoral neck BMD**			
Neural network	$< .0001$	$> .05$	$< .0001$
Gradient boosting	$< .0001$	$< .0001$	–
Random forest	$< .0001$	–	–
**Total hip BMD**			
Neural network	$< .0001$	$< .0001$	$< .0001$
Gradient boosting	$< .0001$	$> .05$	–
Random forest	$< .0001$	–	–
**Total spine BMD**			
Neural network	$< .0001$	$< .0001$	$< .0001$
Gradient boosting	$< .0001$	$< .001$	–
Random forest	$< .0001$	–	–

The Wilcoxon Signed-Rank Test was used to determine all *p* values.

*Lasso regularization with the penalized value of 0.01 was used in the linear regression.

## Discussion

This study presents findings from employing various ML models and linear regression, as well as genotype and phenotype data for BMD prediction in older men. Interestingly, we found that if we use GRS—that is, the summarized genetic risk from associated SNPs—as the genetic predictor in the model, all ML approaches did not perform better than linear regression in predicting BMD. In contrast, if we replace GRS with the 1103 individual risk SNPs as predictors in the model, ML models all had significantly better performance than linear regression for BMD prediction. With the increasing availability of genomic and health big data, ML technologies, which employ a wide-ranging class of algorithms, have increasingly been utilized effectively in medical research, especially in disease prediction. However, our study findings suggested that the conventional approach may be sufficient if we use GRS, a summary metric for genetic risk, as the genetic predictor in the prediction model. In contrast, our study suggests that ML approaches instead may be recommended if a large number of individual genetic variants are included as predictors.

ML models have been used widely for prediction in classification problems, especially for disease prediction. However, studies that utilized ML technologies to predict quantitative traits are still rare. Reportedly, artificial neural networks were utilized for BMD prediction in a small sample of Japanese postmenopausal women, using common risk factors and a BMD previously measured^13^. However, to the best of our knowledge, our study is the first attempt to predict BMD using both advanced ML approaches and genomic information, as well as the first to identify the best ML model for BMD prediction. Our study demonstrated that ML technologies perform better than conventional methods for predicting quantitative traits in complex data that include a large number of genomic variants as predictors.

Risk SNPs identified in GWAS and genome-wide meta-analyses have posed a challenge in conventional statistical analysis because their effect size is very small. Each associated SNP contributes minimally to the variance of BMD. Thus, GRS is widely used to integrate the effects of individual associated SNPs into a single genetic summary variable for prediction research in many studies. Although such an approach improves the prediction ability, many uncertainties remain. For example, this approach does not account for gene interaction and regulation. To address these limitations, we utilized ML approaches in the current study so that individual SNPs can be included to replace GRS in the modeling process. ML approaches have the capacity to handle high dimension data and incorporate the various nonlinear interactions between genetic variants/predictors, which cannot be addressed by conventional modeling methods. Thus, ML approaches provide great potential for improving BMD prediction. In the present study, we employed random forest, gradient boosting, and neural networks, as well as 1103 individual related SNPs, to find a more accurate BMD prediction model. We found that the gradient boosting model performs best in predicting BMD as it has the lowest MSE and MAE, and the highest coefficient of determination in the validation for all three BMD outcomes.

Studies^14,15^ show that the recursive partitioning approaches such as the random forest and gradient boosting have been used to detect genetic loci interaction for the phenotypic outcome^16–19^. The highest predictive performance of the gradient boosting model has been utilized widely in predicting various diseases and outcomes, including hip fractures^20^, sepsis^21^, urinary tract infections^22^, hepatocellular carcinoma^23^, and bioactive molecules^24^. The present study suggested that the gradient boosting approach, combined with individual SNPs as the predictors and considering the interaction of SNPs, can provide a more accurate BMD prediction.

Our study has several strengths. To ensure that our study results were robust, we took the following strategies. First, we have used two metrics, MSE and MAE, to examine and compare the prediction accuracy of the four models we developed. The study findings were consistent between analyses using the two metrics. Second, we performed data analysis separately for the outcome variable BMD measured in three different skeletal regions. The study results were consistent. Finally, we employed the nonparametric Wilcoxon signed-rank test to examine the significance of the different MSE and MAE between any two models so as to ensure the data distribution did not bias the results of the statistical test. We also used Bonferroni corrections for the multiple comparisons in order to ensure our conclusions were robust.

However, our study has some limitations as well. First, the study sample size ($n=5130$) is relatively small for ML methods. ML methods often require a much larger sample size for training. To address this limitation, we used tenfold cross-validation for tuning the hyper-parameters within the training dataset. Therefore, we did not need to allocate part of the study sample for model validation, maximizing the sample size training model. Second, some covariates were not available in the MrOS through dbGaP, including related medications, comorbidities, and physical activities. Lacking these phenotypic variables could have impacted the performance of all prediction models. Third, the MrOS data only included men $\ge 65$ years old and mostly Caucasian (90%), so findings from the present study may not apply to women, younger individuals, or other ethnicities. Finally, rare risk SNPs were less likely to be included for modeling in this study because risk SNPs used in this study were identified from a GWAS study, which likely discovered common but not rare variants^25^. Nevertheless, these limitations are unlikely to have altered our findings in the current study because this is a self-controlled study, with all models developed and validated by the same datasets.

In summary, there was not a significant difference in predicting BMD between various ML models and linear regression if GRS, a metric used to summarize genetic variants, was used for model development. However, when using many individual SNPs as predictors to replace GRS, ML models performed significantly better than linear regression in BMD prediction. Among these ML models, the gradient boost model performed best for BMD prediction. Our study suggests that ML models, especially gradient boosting, can be used to identify patients with low BMD if their genetic information is available. Our study also suggested that when researchers used a large number of genetic variants or other predictors, ML approaches, especially gradient boosting, should be considered. Additionally, more comprehensive studies, especially those including women, young participants, rare genetic variants, and additional risk factors, are warranted to examine further and advance the research findings of the present study.

## Materials and methods

### Data source

The Osteoporotic Fractures in Men Study (MrOS) was used as the data source for this study. MrOS is a federal funded prospective cohort study that was designed to investigate anthropometric, lifestyle, and medical factors associated with bone health in older, community‐dwelling men. Details of the MrOS study design, recruitment, and baseline cohort characteristics have been reported elsewhere^26^. With the approval of the institutional review board at the University of Nevada, Las Vegas, and the National Institute of Health (NIH), the genotype and phenotype data of MrOS were acquired from dbGaP (Accession: phs000373.v1.p1). MrOS consisted of 5130 subjects, all of whom had genotype and phenotype data available for authorized access. All participants provided written informed consent in participating in this study, and all research was performed following relevant guidelines/regulations.

### Study participants

Participants in the MrOS were at least 65 years old, community-dwelling, ambulatory, and had not received bilateral hip replacement^27^ at the study entry. At enrollment, participants had to provide self-reported data, understand and sign the written informed consent, complete the self-administered questionnaire, attend a clinic visit, and complete the anthropometric, DEXA vertebral X-ray procedures. The participants could not have a medical condition that would result in imminent death, which was based on the investigators’ judgment. A total of 5994 men were enrolled between March 2000 and April 2002, all of whom were from six communities in the United States (Birmingham, AL; Minneapolis, MN; Palo Alto, CA; Pittsburgh, PA; Portland, OR; and San Diego, CA.)^28^.

### Outcome BMD measurements

Total body, total femur BMD, and lumbar spine (L1 to L4) were measured using a fan-beam dual-energy X-ray absorptiometry (QDR 4500 W, Hologic, Inc., Bedford, MA, USA) at the second visit of MrOS. Participants were scanned for BMD measurements by licensed densitometrists, using standardized procedures. All DXA operators were centrally certified, based on the evaluation results of scanning and analysis techniques. Cross-calibrations conducted before participants’ visits for BMD measurement found no linear differences across scanners. The maximum percentage difference between scanners was 1.4% in mean BMD of the total spine^29^. No shifts or drifts in scanner performance was found, based on daily quality control in each clinical center.

### Assessment of covariates

Bone health-related information, including demographics, clinical history, medications, and lifestyle factors, was obtained by self-administered questionnaires. The information collected contained the variables used in this study, including age, race, smoking, and alcohol consumption. Height (cm) was measured using a Harpenden stadiometer, and weight (kg) was measured by a standard balance beam or an electric scale.

Smoking was categorized as “never,” “past,” and “current.” Alcohol intake was quantified in terms of the usual number of drinks per day. Walking speed was determined by timed completion of a 6-m course, performed at each participant’s typical walking speed. Mobility limitations were quantified by using a participant’s ability to rise from a chair without using his arms and his ability to complete five chair stands. Each participant’s function status was quantified by assessing daily living difficulty on a scale of 0–3, with five instrumental activities of daily living, which include walking on level ground, climbing steps, preparing meals, performing housework, and shopping.

### Genotyping data

Whole blood samples at the baseline were used for DNA extraction. Consent for DNA use was obtained through written permission. Quality-control genotype data files were acquired through dbGaP and implemented with PLINK^30^. Genotype imputation was conducted at the Sanger Imputation Server^31^. The Haplotype Reference Consortium imputation reference panel^32^ and the Positional Burrows–Wheeler Transform imputing algorithm^33^ were used to ensure high quality of genotype imputation. Based on the study published by Morris et al. in 2019, a total of 1103 associated SNPs were extracted for this analysis^10^. These 1103 SNPs were conditionally independent at genome-wide significance $(p<6.6\times {10}^{-9})$ with BMD estimated by heel quantitative ultrasound (eBMD)^10^. All the 1103 SNPs were successfully imputed in the MrOS data and were included in the analysis. The imputation quality was excellent, with a mean ${R}^{2}$ of 0.99. The Chi-square test was used to examine the correlations between these SNPs in the study population, and we found that 58% of these SNPs are not independent. Among the SNPs that are not independent (635 SNPs, 58%) in the Lasso linear regression model, the estimated coefficients of 88% SNPs (59) became zero, which were eliminated in the final linear regression model. The Analysis of Variance test^34^ was employed to examine the association between each SNP and each BMD outcome. Genotyping for MrOS samples was performed with the Illumina HumanOmni1_Quad_v1-0 H array. A total of autosomal 934,940 SNPs passed the quality control with the following criteria: minor allele frequency $\ge 0.05$ individual missingness < 5%, SNPs call rate > 95%, and Hardy–Weinberg equilibrium $p$ value < 0.0001.

### Genetic risk score

A genetic risk score (GRS) is a standardized metric that allows the composite assessment of genetic risk in complex traits. The GRS was derived from the number of risk alleles and their effect size for each study subject. We performed a linkage disequilibrium (LD) pruning in advance to eliminate possible LD between SNPs; however, none of the SNPs were eligible for removal during the pruning process. The weighted GRS was then calculated with the algorithms described previously^35^. Briefly, for each participant in MrOS, weighted GRS was calculated by summing the number of risk alleles at each locus weighted by regression coefficients related to BMD^10^.

### Data processing

**Figure 4:**
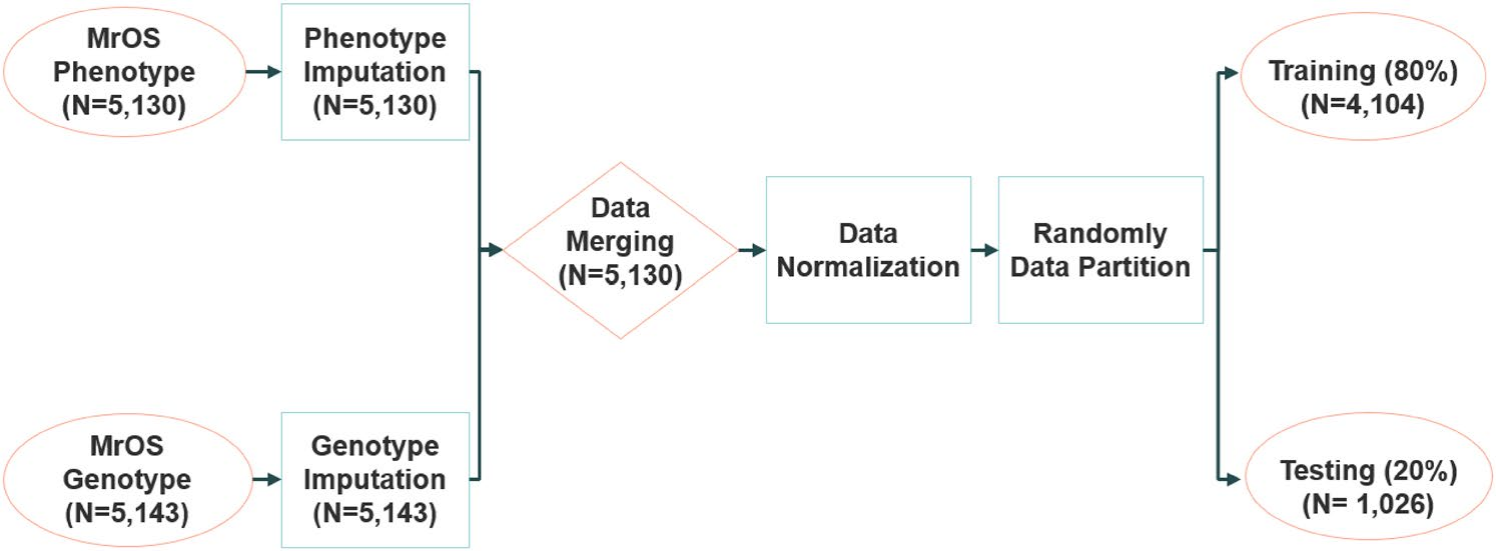
Overview of data process flow.

Figure 4 shows an overview of our data process flow for this study. After genotype imputation, the phenotype data set (n = 5130) and genotype data set (n = 5143) were merged, and 13 participants were removed from the analysis due to the lack of all phenotype data. After combining the data set, each phenotype variable has less than 1% missing value in 5130 participants. One participant has a missing value of femoral neck and total hip BMD, and ten participants have a missing value of total spine BMD. All other variables do not have the missing value. We normalized all continuous variables in the data, then randomly divided the dataset into a training set (80%, n = 4104) and a test set (20%, n = 1026). The median imputation^36^, the most common imputation method for continuous variables, was used to replace missing values in the data, maximizing the sample size for analysis.

### Data analysis

The outcome variables were BMD measured from various skeletal regions, which included femoral neck, total spine, and total hip. The predictors included GRS, age, race, body weight, height, smoking, alcohol consumption, walking speed, impairment of instrumental activities of daily living, and mobility limitations. Linear regression, random forest, gradient boosting, and neural network with backpropagation were used to train the model separately. The neural network with backpropagation consists of three sequential layers: input, hidden, and output. The number of hidden layers was considered as the hyper-parameters in this study^37^. Lasso regularization, with the penalized value of 0.01, was used to address the overfitting problem in the first layer of the neural network model^38^. The rectified linear unit, Sigmoid, and Hyperbolic tangent activation functions were considered^39^. We also conducted analyses that replaced the GRS with the 1103 individual SNPs in each model. We encoded each risk SNP as three different genotypes (dominant homozygous allele, heterozygotes, homozygous minor allele) with 0, 1, and 2.

In model training, tenfold cross-validation was used for hyper-parameter optimization. We divided the training set into 10-folds, and chose one fold as a validation set, with the remaining folds used as the training set. We used Scikit-learn’s randomized search cross-validation^40^ to find the best hyperparameters for different algorithms. To avoid the high computational burden in searching for all the possible parameters, we utilized randomized search cross-validation to find the optimal hyper-parameters by sampling a different combination of parameters from the given distribution^41^. The training set was used to train and construct linear regression models, random forest, gradient boosting, and neural networks. For linear regression modeling, as multicollinearity may occur when including 1103 individual SNPs in the model as predictors, we implemented the three shrinkage methods of Lasso^42^, Ridge regression^43^, and Elastic Net^44^ separately for the linear regression model. We found that lasso regularization with the penalized value of 0.01 showed the lowest error rate in the linear regression model. For each other type of ML, we tested different combinations of hyper-parameters in the training sets to obtain the most optimal model for each ML (Table S2). Each combination of various hyper-parameters was evaluated by the mean squared error or mean absolute error of the model. For each type of ML, the set of parameters with the lowest mean squared error or mean absolute error in the testing data was selected. The early stopping criteria of ML training were defined as no decrease in the MSE or MAE by 0.001 in the training data set of 100 consecutive iterations. The early stopping procedure was applied in each 10-folds cross-validation of training dataset and each early stopping iteration number was recorded. Then, the average early stopping iteration was calculated for each model.

The testing set (20%) was used to evaluate the prediction performance of the developed model. Metrics for model performance evaluation are mean squared error^45^, mean absolute error^46^, and the coefficient of determination $\left({R}^{2}\right)$^47^. We adopted two metrics:

- Mean Squared Error (MSE):
  $$MSE= \frac{1}{n}\sum_{i=1}^{n}{\left({y}_{i}- \widehat{{y}_{i}}\right)}^{2}$$
- Mean Absolute Error (MAE*)*:
  $$MAE= \frac{1}{n}\sum_{i=1}^{n}|{y}_{i}- \widehat{{y}_{i}}|$$

where $n$ is the sample size, ${y}_{i}$ is the actual value for each observation, and $\widehat{{y}_{i}}$ is the estimated value for each observation from the model. We first used MSE as a loss function to develop the model in a training set. We also calculated MSE for each model in the test set and used the MSE for model evaluation. We then reanalyzed the data by replacing MSE with MAE, in which MAE was used as a loss function to develop the model in a training set and was calculated in the test set for model evaluation. We also calculated the coefficient of determination for each model in the training and testing dataset. Wilcoxon signed-rank test was employed to examine the difference of MSE or MAE between ML models because the data distribution assumption for the student t-test was not met. All of the analyses were performed in the Python Software Foundation and Python Language Reference, version 3.7.3, with the package Scikit-learn: Machine Learning in Python^40^, and the *glmnet*^48^ packages of R software was used for Lasso, Ridge regression, and Elastic Net.

## Supplementary Information

Supplementary Tables

## Data Availability

The data/analyses presented in the current publication are based on the use of study data downloaded from the dbGaP website under phs000373.v1.p1 (https://www.ncbi.nlm.nih.gov/projects/gap/cgi-bin/study.cgi?study_id=phs000373.v1.p1). The code required for replicating the results reported in this paper is available at https://github.com/wulabunlv/BMD_ML.

## Abbreviations

MrOS: Osteoporotic Fractures in Men Study
BMD: Bone Mineral Density
ML: Machine Learning
GRS: Genetic Risk Score
LR: Linear Regression
RF: Random Forest
GB: Gradient Boosting
NN: Neural Network
SNPs: Single Nucleotide Polymorphisms
GWAS: Genome-Wide Association Study
