# Supplementary material for: Machine learning approaches for the prediction of bone mineral density by using genomic and phenotypic data of 5130 older men
Source: Sci Rep. 2021 Feb 24;11:4482. doi: 10.1038/s41598-021-83828-3 (PMC7904941; doi:10.1038/s41598-021-83828-3)
Supplement: Supplementary file 1 — Supplementary Tables [file 41598_2021_83828_MOESM1_ESM.docx]

**Title:** Machine learning approaches for the prediction of bone mineral density by using genomic and phenotypic data of 5130 older men

Qing Wu, M.D., Sc.D^1,2*^  (0000-0003-4679-8903)

Fatma Nasoz, Ph.D.^3,4^

Jongyun Jung, MS^1,2^ (0000-0002-9258-5397)

Bibek Bhattarai, MS^3^

Mira V Han Ph.D.^1,5^

Robert A Greenes, M.D., Ph.D.^6,7^

Kenneth G. Saag, M.D., MSc^8^

**Author Affiliations:**

^1^Nevada Institute of Personalized Medicine (Dr. Qing Wu, Jongyun Jung, and Mira V. Han), University of Nevada, Las Vegas, Nevada;

^2^ Department of Epidemiology and Biostatistics, School of Public Health (Dr. Qing Wu and Jongyun Jung), University of Nevada, Las Vegas, Nevada;

^3^Department of Computer Science (Dr. Fatma Nasoz and Bibek Bhattarai), University of Nevada, Las Vegas, Nevada;

^4^The Lincy Institute (Dr. Fatma Nasoz), University of Nevada, Las Vegas, Nevada;

^5^School of Life Sciences (Mira V. Han), University of Nevada, Las Vegas, Nevada

^6^Department of Biomedical Informatics, College of Health Solutions, Arizona State University, Phoenix, Arizona;

^7^Department of Health Science Research, Mayo Clinic, Scottsdale, Arizona;

^8^Department of Medicine, Division of Clinical Immunology and Rheumatology, the University of Alabama at Birmingham, Birmingham, AL

*Corresponding author: [qing.wu@unlv.edu](mailto:qing.wu@unlv.edu)

Supplemental Material

Table S1. Pearson’s Correlation coefficients between the bone mineral density and the phenotypic risk factors.

| Variable | Femoral Neck BMD | Total Hip BMD | Total Spine BMD |
| --- | --- | --- | --- |
| Age | -0.185* | -0.193* | -0.047* |
| Height | 0.150* | 0.164* | 0.103* |
| Weight | 0.361* | 0.408* | 0.189* |
| Alcohol Use | 0.075* | 0.061* | 0.038* |
| GRS | -0.022 | -0.023 | -0.030 |
| Impairment of Instrumental Activities of Daily Living | -0.019 | -0.042* | -0.055* |
| Walking Speed | 0.006 | 0.025 | 0.045 |

Notes: *$\boldsymbol{p}$–value $\boldsymbol{<0.05}$, BMD: Bone Mineral Density, GRS: Genetic Risk Score

Table S2. Different hyper-parameters settings for machine learning algorithms in the training data.

| Algorithms | Hyper-parameters | Value Tested | Value Used |
| --- | --- | --- | --- |
| Random Forest | Whether samples are drawn with replacement. If false, sampling without replacement is performed | True, False | True |
|  | The maximum depth of the tree | 3, 5, 10, 20 | 20 |
|  | The number of trees in the forest | 200, 400, 600, 800, 1000 | 600 |
|  | The number of features to consider when looking for the best split | Auto, Square root | Auto |
|  | The minimum number of samples required to be at a leaf node | 1, 2, 4 | 2 |
|  | The minimum number of samples required to split an internal node | 2, 5, 10 | 5 |
| Gradient Boosting | The number of trees in the forest | 200, 400, 600, 800, 1000 | 600 |
|  | The loss function to be optimized | Least square regression (LSR), Least absolute deviation (LAD) | LSR |
|  | The depth of trees | 3, 5, 10, 20 | 10 |
|  | The minimum number of samples required to be at a leaf node | 1, 2, 4 | 2 |
|  | The minimum of samples required to split | 2, 5, 10 | 5 |
|  | The learning rate shrinks the contribution of each tree | 0.1, 0.05, 0.01, 0.005, 0.001 | 0.01 |
| Neural Network | Batch size | 10, 20 | 20 |
|  | The number of neurons in the first layer | 100, 200, 300, 500, 1000 | 500 |
|  | The number of hidden layers | 2, 3, 4, 5, 6, 7, 8, 9, 10 | 3 |
|  | Activation function | Sigmoid, Rectified linear unit (ReLU), Hyperbolic tangent (Tanh) | Sigmoid |

Table S3. The performance of the coefficient of determination $\left( R^{2} \right)$ for each different model in predicting various BMD of testing dataset (n = 1,026).

|  | Model 1^a^ | |  | Model 2^b^ | |  | Model 3^c^ | |
| --- | --- | --- | --- | --- | --- | --- | --- | --- |
|  | Testing | Training |  | Testing | Training |  | Testing | Training |
| Femoral Neck BMD |  |  |  |  |  |  |  |  |
| Linear Regression | 0.1225 | 0.1473 |  | 0.1356 | 0.1587 |  | 0.0097 | 0.0104 |
| Random Forest | 0.1206 | 0.1536 |  | 0.1392 | 0.1635 |  | 0.0135 | 0.0889 |
| Gradient Boosting | 0.1234 | 0.1551 |  | 0.1402 | 0.1639 |  | 0.0155 | 0.0954 |
| Neural Network | 0.1276 | 0.1469 |  | 0.1321 | 0.1592 |  | 0.0102 | 0.0605 |
| Total Hip BMD |  |  |  | |  |  |  | |
| Linear Regression | 0.1576 | 0.1725 |  | 0.1794 | 0.1981 |  | 0.0102 | 0.0336 |
| Random Forest | 0.1539 | 0.1694 |  | 0.1825 | 0.2056 |  | 0.0128 | 0.0549 |
| Gradient Boosting | 0.1595 | 0.1769 |  | 0.1839 | 0.2116 |  | 0.0147 | 0.0754 |
| Neural Network | 0.1625 | 0.1793 |  | 0.1802 | 0.2032 |  | 0.0115 | 0.0649 |
| Total Spine BMD |  |  |  | |  |  |  | |
| Linear Regression | 0.0687 | 0.0719 |  | 0.0871 | 0.0746 |  | 0.0213 | 0.0418 |
| Random Forest | 0.0704 | 0.0768 |  | 0.1098 | 0.0873 |  | 0.0374 | 0.0658 |
| Gradient Boosting | 0.0747 | 0.0847 |  | 0.1114 | 0.0938 |  | 0.0402 | 0.0832 |
| Neural Network | 0.0695 | 0.0791 |  | 0.0919 | 0.0801 |  | 0.0392 | 0.0582 |

^a^ Model 1: Used GRS and phenotypic covariates as the predictors

^b^ Model 2: Used 1,103 SNPs and phenotypic covariates as the predictors. Lasso regularization, with the penalized value of 0.01, was used in the linear regression of model 2.

^c^ Model 3: Used only 1,103 SNPs as the predictors. Lasso regularization, with the penalized value of 0.01, was used in the linear regression of model 3.

Table S4. The best iteration numbers from the training of each model. The early stopping procedure was applied in each 10-folds cross-validation of the training dataset, and each early stopping iteration number was recorded. Then, the average early stopping iteration was calculated for each model.

|  | Model 1^a^ | Model 2^b^ | |
| --- | --- | --- | --- |
| Femoral Neck BMD |  |  | |
| Linear Regression | 15 | 96 | |
| Random Forest | 10 | 11 | |
| Gradient Boosting | 9 | 10 | |
| Neural Network | 13 | 14 | |
| Total Hip BMD |  | | |
| Linear Regression | 18 | | 85 |
| Random Forest | 12 | | 15 |
| Gradient Boosting | 10 | | 13 |
| Neural Network | 15 | | 18 |
| Total Spine BMD |  | | |
| Linear Regression | 19 | | 96 |
| Random Forest | 12 | | 20 |
| Gradient Boosting | 11 | | 14 |
| Neural Network | 15 | | 35 |

^a^ Model 1: Used GRS and phenotypic covariates as the predictors

^b^ Model 2: Used 1,103 SNPs and phenotypic covariates as the predictors. Lasso regularization, with the penalized value of 0.01, was used in the linear regression of model 2.

Table S5. The result of the ANOVA test for the association of 1,103 SNPs with each BMD region. Each SNPs with the number of alternative alleles expressed at the end of rsID in the SNPs column. All of the SNPs estimated coefficients were referenced to no alternative allele.

|  | Total Hip BMD | | |  | Femoral Neck BMD | | |  | Total Spine BMD | | |
| --- | --- | --- | --- | --- | --- | --- | --- | --- | --- | --- | --- |
| SNPs | Beta | SE | P-value |  | Beta | SE | P-value |  | Beta | SE | P-value |
| rs9984655-2 | -0.051 | 0.053 | 0.334 |  | -0.073 | 0.054 | 0.170 |  | -0.007 | 0.053 | 0.893 |
| rs9984655-1 | -0.021 | 0.038 | 0.588 |  | -0.024 | 0.038 | 0.521 |  | 0.008 | 0.038 | 0.840 |
| rs9976876-2 | 0.022 | 0.052 | 0.667 |  | 0.028 | 0.052 | 0.595 |  | 0.003 | 0.052 | 0.951 |
| rs9976876-1 | 0.032 | 0.040 | 0.424 |  | 0.045 | 0.041 | 0.271 |  | 0.022 | 0.040 | 0.588 |
| rs9975345-2 | 0.009 | 0.049 | 0.855 |  | 0.048 | 0.049 | 0.330 |  | 0.040 | 0.049 | 0.416 |
| rs9975345-1 | 0.025 | 0.046 | 0.587 |  | 0.037 | 0.046 | 0.429 |  | 0.032 | 0.046 | 0.488 |
| rs9974172-2 | -0.001 | 0.135 | 0.996 |  | -0.073 | 0.135 | 0.590 |  | -0.056 | 0.134 | 0.678 |
| rs9974172-1 | -0.037 | 0.040 | 0.354 |  | -0.053 | 0.040 | 0.192 |  | -0.038 | 0.040 | 0.340 |
| rs9952412-2 | -0.038 | 0.049 | 0.442 |  | -0.041 | 0.049 | 0.407 |  | -0.046 | 0.049 | 0.349 |
| rs9952412-1 | -0.022 | 0.040 | 0.584 |  | 0.004 | 0.040 | 0.925 |  | -0.010 | 0.040 | 0.795 |
| rs9927137-2 | -0.086 | 0.050 | 0.084 |  | -0.068 | 0.050 | 0.171 |  | -0.080 | 0.050 | 0.106 |
| rs9927137-1 | 0.023 | 0.041 | 0.577 |  | 0.038 | 0.041 | 0.350 |  | -0.006 | 0.041 | 0.887 |
| rs9924983-2 | -0.087 | 0.049 | 0.077 |  | -0.056 | 0.049 | 0.260 |  | -0.046 | 0.049 | 0.354 |
| rs9924983-1 | -0.062 | 0.044 | 0.158 |  | -0.033 | 0.044 | 0.460 |  | -0.024 | 0.044 | 0.590 |
| rs9921222-2 | -0.047 | 0.050 | 0.345 |  | -0.077 | 0.051 | 0.129 |  | -0.112 | 0.050 | 0.026 |
| rs9921222-1 | -0.102 | 0.040 | 0.011 |  | -0.103 | 0.040 | 0.010 |  | -0.036 | 0.040 | 0.365 |
| rs9914258-2 | -0.062 | 0.049 | 0.209 |  | -0.084 | 0.050 | 0.090 |  | -0.117 | 0.049 | 0.018 |
| rs9914258-1 | 0.024 | 0.047 | 0.604 |  | 0.024 | 0.047 | 0.614 |  | -0.035 | 0.046 | 0.446 |
| rs9909922-2 | 0.150 | 0.169 | 0.374 |  | 0.184 | 0.170 | 0.278 |  | 0.297 | 0.168 | 0.078 |
| rs9909922-1 | -0.040 | 0.044 | 0.364 |  | -0.039 | 0.044 | 0.379 |  | 0.020 | 0.044 | 0.650 |
| rs9908318-2 | 0.073 | 0.059 | 0.215 |  | 0.077 | 0.059 | 0.192 |  | 0.067 | 0.059 | 0.254 |
| rs9908318-1 | 0.037 | 0.060 | 0.531 |  | 0.040 | 0.060 | 0.501 |  | 0.035 | 0.059 | 0.558 |
| rs9901257-2 | 0.018 | 0.049 | 0.716 |  | 0.015 | 0.049 | 0.760 |  | -0.050 | 0.049 | 0.310 |
| rs9901257-1 | -0.022 | 0.038 | 0.573 |  | -0.006 | 0.039 | 0.872 |  | -0.018 | 0.038 | 0.635 |
| rs9896306-2 | 0.068 | 0.063 | 0.281 |  | 0.037 | 0.063 | 0.561 |  | 0.018 | 0.063 | 0.773 |
| rs9896306-1 | 0.061 | 0.036 | 0.091 |  | 0.036 | 0.036 | 0.318 |  | -0.002 | 0.036 | 0.953 |
| rs9873544-2 | 0.029 | 0.055 | 0.601 |  | 0.041 | 0.056 | 0.465 |  | -0.052 | 0.055 | 0.351 |
| rs9873544-1 | -0.032 | 0.037 | 0.381 |  | -0.005 | 0.037 | 0.889 |  | -0.070 | 0.037 | 0.056 |
| rs983034-2 | -0.001 | 0.054 | 0.980 |  | -0.011 | 0.054 | 0.832 |  | -0.060 | 0.054 | 0.260 |
| rs983034-1 | 0.051 | 0.038 | 0.181 |  | 0.049 | 0.038 | 0.199 |  | 0.020 | 0.038 | 0.607 |
| rs9813107-2 | 0.264 | 0.130 | 0.043 |  | 0.230 | 0.131 | 0.078 |  | 0.256 | 0.130 | 0.048 |
| rs9813107-1 | -0.004 | 0.041 | 0.931 |  | -0.016 | 0.041 | 0.701 |  | -0.029 | 0.041 | 0.478 |
| rs9626436-2 | 0.051 | 0.123 | 0.681 |  | 0.053 | 0.124 | 0.668 |  | -0.110 | 0.123 | 0.372 |
| rs9626436-1 | 0.072 | 0.127 | 0.574 |  | 0.088 | 0.128 | 0.492 |  | -0.048 | 0.127 | 0.706 |
| rs9613775-2 | -0.050 | 0.079 | 0.523 |  | -0.003 | 0.079 | 0.971 |  | -0.011 | 0.078 | 0.884 |
| rs9613775-1 | -0.065 | 0.081 | 0.423 |  | -0.034 | 0.081 | 0.680 |  | -0.071 | 0.081 | 0.377 |
| rs9606138-2 | 0.081 | 0.154 | 0.602 |  | 0.221 | 0.155 | 0.155 |  | -0.070 | 0.154 | 0.649 |
| rs9606138-1 | -0.021 | 0.047 | 0.653 |  | -0.019 | 0.047 | 0.691 |  | -0.029 | 0.047 | 0.539 |
| rs960192-2 | 0.002 | 0.050 | 0.973 |  | -0.037 | 0.050 | 0.460 |  | 0.091 | 0.050 | 0.068 |
| rs960192-1 | 0.048 | 0.038 | 0.209 |  | 0.028 | 0.038 | 0.470 |  | 0.112 | 0.038 | 0.003 |
| rs9594738-2 | -0.138 | 0.054 | 0.011 |  | -0.087 | 0.055 | 0.110 |  | -0.204 | 0.054 | 0.000 |
| rs9594738-1 | -0.118 | 0.042 | 0.005 |  | -0.098 | 0.042 | 0.019 |  | -0.125 | 0.042 | 0.003 |
| rs9561331-2 | -0.204 | 0.117 | 0.080 |  | -0.255 | 0.117 | 0.030 |  | -0.098 | 0.117 | 0.399 |
| rs9561331-1 | -0.030 | 0.041 | 0.464 |  | -0.049 | 0.042 | 0.243 |  | 0.020 | 0.041 | 0.622 |
| rs9553006-2 | 0.001 | 0.058 | 0.984 |  | -0.044 | 0.058 | 0.450 |  | -0.050 | 0.058 | 0.389 |
| rs9553006-1 | -0.017 | 0.058 | 0.771 |  | -0.063 | 0.058 | 0.277 |  | -0.063 | 0.057 | 0.275 |
| rs9532858-2 | 0.033 | 0.056 | 0.560 |  | 0.078 | 0.057 | 0.166 |  | 0.071 | 0.056 | 0.207 |
| rs9532858-1 | -0.004 | 0.056 | 0.949 |  | 0.089 | 0.056 | 0.111 |  | 0.016 | 0.056 | 0.777 |
| rs9530279-2 | -0.058 | 0.064 | 0.361 |  | -0.083 | 0.064 | 0.193 |  | -0.097 | 0.063 | 0.127 |
| rs9530279-1 | -0.025 | 0.036 | 0.492 |  | -0.033 | 0.036 | 0.361 |  | -0.060 | 0.036 | 0.092 |
| rs9521510-2 | -0.084 | 0.056 | 0.139 |  | -0.028 | 0.057 | 0.617 |  | -0.034 | 0.056 | 0.541 |
| rs9521510-1 | -0.041 | 0.037 | 0.263 |  | -0.030 | 0.037 | 0.408 |  | 0.017 | 0.036 | 0.645 |
| rs9513510-2 | -0.092 | 0.063 | 0.141 |  | -0.095 | 0.063 | 0.129 |  | -0.086 | 0.062 | 0.169 |
| rs9513510-1 | -0.059 | 0.063 | 0.348 |  | -0.047 | 0.064 | 0.458 |  | -0.050 | 0.063 | 0.426 |
| rs9509989-2 | -0.231 | 0.146 | 0.113 |  | -0.312 | 0.146 | 0.033 |  | 0.017 | 0.145 | 0.909 |
| rs9509989-1 | -0.071 | 0.041 | 0.085 |  | -0.060 | 0.041 | 0.147 |  | 0.007 | 0.041 | 0.870 |
| rs949782-2 | -0.078 | 0.069 | 0.256 |  | -0.084 | 0.069 | 0.227 |  | -0.086 | 0.069 | 0.212 |
| rs949782-1 | -0.012 | 0.070 | 0.863 |  | 0.012 | 0.071 | 0.862 |  | -0.001 | 0.070 | 0.986 |
| rs9482770-2 | -0.106 | 0.051 | 0.036 |  | -0.100 | 0.051 | 0.049 |  | -0.061 | 0.050 | 0.225 |
| rs9482770-1 | -0.052 | 0.046 | 0.257 |  | -0.057 | 0.046 | 0.215 |  | -0.028 | 0.046 | 0.549 |
| rs947091-2 | 0.060 | 0.049 | 0.225 |  | 0.068 | 0.049 | 0.169 |  | 0.067 | 0.049 | 0.173 |
| rs947091-1 | -0.010 | 0.044 | 0.829 |  | -0.020 | 0.044 | 0.656 |  | 0.008 | 0.044 | 0.848 |
| rs9466056-2 | -0.054 | 0.053 | 0.304 |  | -0.059 | 0.053 | 0.262 |  | -0.049 | 0.052 | 0.347 |
| rs9466056-1 | 0.015 | 0.038 | 0.694 |  | 0.019 | 0.038 | 0.623 |  | -0.028 | 0.038 | 0.454 |
| rs945508-2 | 0.008 | 0.049 | 0.861 |  | -0.021 | 0.049 | 0.670 |  | -0.028 | 0.048 | 0.568 |
| rs945508-1 | -0.014 | 0.039 | 0.723 |  | -0.015 | 0.040 | 0.702 |  | -0.094 | 0.039 | 0.016 |
| rs9447004-2 | 0.002 | 0.048 | 0.963 |  | 0.019 | 0.048 | 0.691 |  | 0.026 | 0.048 | 0.589 |
| rs9447004-1 | -0.042 | 0.042 | 0.323 |  | -0.025 | 0.042 | 0.562 |  | 0.075 | 0.042 | 0.074 |
| rs9417567-2 | -0.005 | 0.110 | 0.965 |  | -0.075 | 0.111 | 0.498 |  | 0.005 | 0.110 | 0.962 |
| rs9417567-1 | 0.024 | 0.041 | 0.547 |  | 0.012 | 0.041 | 0.764 |  | 0.012 | 0.041 | 0.763 |
| rs939666-2 | -0.136 | 0.095 | 0.155 |  | 0.021 | 0.096 | 0.825 |  | 0.058 | 0.095 | 0.545 |
| rs939666-1 | -0.085 | 0.094 | 0.365 |  | 0.061 | 0.094 | 0.515 |  | 0.090 | 0.094 | 0.334 |
| rs938295-2 | -0.009 | 0.082 | 0.912 |  | 0.010 | 0.082 | 0.899 |  | 0.001 | 0.082 | 0.992 |
| rs938295-1 | -0.033 | 0.084 | 0.699 |  | 0.014 | 0.085 | 0.867 |  | 0.018 | 0.084 | 0.827 |
| rs9379084-2 | -0.038 | 0.138 | 0.782 |  | -0.002 | 0.138 | 0.990 |  | -0.020 | 0.137 | 0.885 |
| rs9379084-1 | -0.021 | 0.042 | 0.612 |  | -0.018 | 0.042 | 0.666 |  | 0.008 | 0.042 | 0.850 |
| rs9378485-2 | 0.003 | 0.052 | 0.954 |  | -0.004 | 0.052 | 0.945 |  | 0.015 | 0.052 | 0.766 |
| rs9378485-1 | -0.019 | 0.038 | 0.606 |  | -0.034 | 0.038 | 0.367 |  | -0.023 | 0.038 | 0.546 |
| rs9376164-2 | -0.142 | 0.122 | 0.247 |  | -0.175 | 0.123 | 0.155 |  | 0.014 | 0.122 | 0.911 |
| rs9376164-1 | -0.064 | 0.042 | 0.130 |  | -0.089 | 0.043 | 0.037 |  | -0.106 | 0.042 | 0.012 |
| rs9375477-2 | -0.164 | 0.096 | 0.086 |  | -0.199 | 0.096 | 0.039 |  | -0.201 | 0.095 | 0.035 |
| rs9375477-1 | -0.160 | 0.097 | 0.099 |  | -0.168 | 0.097 | 0.085 |  | -0.189 | 0.097 | 0.050 |
| rs9372944-2 | 0.087 | 0.072 | 0.223 |  | 0.127 | 0.072 | 0.077 |  | -0.083 | 0.071 | 0.246 |
| rs9372944-1 | 0.133 | 0.074 | 0.073 |  | 0.181 | 0.074 | 0.015 |  | -0.082 | 0.074 | 0.269 |
| rs9364386-2 | 0.073 | 0.099 | 0.462 |  | 0.048 | 0.100 | 0.631 |  | -0.041 | 0.099 | 0.679 |
| rs9364386-1 | -0.018 | 0.038 | 0.641 |  | 0.011 | 0.038 | 0.774 |  | 0.011 | 0.038 | 0.781 |
| rs933561-2 | 0.011 | 0.057 | 0.841 |  | 0.019 | 0.057 | 0.740 |  | -0.031 | 0.057 | 0.580 |
| rs933561-1 | -0.050 | 0.037 | 0.173 |  | -0.040 | 0.037 | 0.281 |  | 0.001 | 0.037 | 0.985 |
| rs9332407-2 | 0.063 | 0.050 | 0.207 |  | 0.060 | 0.050 | 0.228 |  | -0.029 | 0.050 | 0.555 |
| rs9332407-1 | 0.034 | 0.045 | 0.452 |  | 0.028 | 0.045 | 0.527 |  | 0.011 | 0.045 | 0.808 |
| rs932536-2 | -0.029 | 0.157 | 0.853 |  | -0.055 | 0.158 | 0.728 |  | 0.076 | 0.156 | 0.626 |
| rs932536-1 | -0.043 | 0.043 | 0.324 |  | -0.014 | 0.043 | 0.740 |  | -0.110 | 0.043 | 0.011 |
| rs932203-2 | 0.006 | 0.048 | 0.895 |  | 0.033 | 0.048 | 0.489 |  | 0.050 | 0.048 | 0.302 |
| rs932203-1 | 0.054 | 0.043 | 0.205 |  | 0.038 | 0.043 | 0.373 |  | 0.086 | 0.043 | 0.044 |
| rs9310995-2 | 0.038 | 0.050 | 0.440 |  | -0.008 | 0.050 | 0.875 |  | -0.024 | 0.050 | 0.630 |
| rs9310995-1 | 0.016 | 0.047 | 0.725 |  | -0.016 | 0.047 | 0.741 |  | -0.045 | 0.047 | 0.335 |
| rs9299597-2 | -0.066 | 0.089 | 0.455 |  | -0.009 | 0.089 | 0.920 |  | 0.160 | 0.088 | 0.071 |
| rs9299597-1 | 0.050 | 0.039 | 0.195 |  | 0.043 | 0.039 | 0.270 |  | 0.092 | 0.039 | 0.017 |
| rs9296151-2 | 0.007 | 0.225 | 0.977 |  | 0.190 | 0.226 | 0.401 |  | 0.323 | 0.224 | 0.150 |
| rs9296151-1 | 0.013 | 0.061 | 0.837 |  | 0.064 | 0.061 | 0.295 |  | -0.046 | 0.061 | 0.447 |
| rs9290351-2 | -0.052 | 0.121 | 0.668 |  | -0.133 | 0.121 | 0.274 |  | -0.151 | 0.120 | 0.209 |
| rs9290351-1 | -0.040 | 0.123 | 0.748 |  | -0.133 | 0.124 | 0.281 |  | -0.072 | 0.123 | 0.556 |
| rs914615-2 | -0.046 | 0.049 | 0.351 |  | 0.007 | 0.050 | 0.890 |  | -0.093 | 0.049 | 0.059 |
| rs914615-1 | 0.013 | 0.044 | 0.761 |  | 0.015 | 0.044 | 0.736 |  | -0.038 | 0.044 | 0.389 |
| rs913257-2 | 0.019 | 0.049 | 0.697 |  | -0.013 | 0.049 | 0.785 |  | 0.050 | 0.049 | 0.309 |
| rs913257-1 | 0.036 | 0.041 | 0.378 |  | 0.037 | 0.041 | 0.361 |  | -0.005 | 0.041 | 0.912 |
| rs901865-2 | -0.054 | 0.099 | 0.586 |  | 0.007 | 0.099 | 0.945 |  | 0.023 | 0.098 | 0.815 |
| rs901865-1 | -0.058 | 0.101 | 0.564 |  | -0.014 | 0.101 | 0.889 |  | 0.042 | 0.101 | 0.679 |
| rs900399-2 | 0.053 | 0.051 | 0.304 |  | 0.050 | 0.051 | 0.331 |  | 0.026 | 0.051 | 0.616 |
| rs900399-1 | 0.044 | 0.038 | 0.238 |  | 0.084 | 0.038 | 0.026 |  | 0.036 | 0.038 | 0.345 |
| rs900348-2 | 0.057 | 0.086 | 0.506 |  | 0.067 | 0.086 | 0.436 |  | 0.060 | 0.085 | 0.485 |
| rs900348-1 | 0.085 | 0.087 | 0.331 |  | 0.080 | 0.088 | 0.362 |  | 0.077 | 0.087 | 0.379 |
| rs899631-2 | 0.009 | 0.051 | 0.853 |  | 0.037 | 0.051 | 0.466 |  | 0.004 | 0.051 | 0.935 |
| rs899631-1 | 0.028 | 0.049 | 0.571 |  | 0.053 | 0.049 | 0.281 |  | -0.023 | 0.049 | 0.631 |
| rs890074-2 | -0.024 | 0.048 | 0.610 |  | -0.022 | 0.048 | 0.654 |  | -0.055 | 0.048 | 0.252 |
| rs890074-1 | -0.029 | 0.041 | 0.480 |  | -0.020 | 0.041 | 0.625 |  | -0.043 | 0.041 | 0.296 |
| rs884205-2 | -0.072 | 0.075 | 0.339 |  | -0.006 | 0.075 | 0.935 |  | -0.099 | 0.075 | 0.184 |
| rs884205-1 | -0.055 | 0.036 | 0.134 |  | -0.050 | 0.037 | 0.174 |  | -0.041 | 0.036 | 0.265 |
| rs87-2 | -0.051 | 0.074 | 0.487 |  | -0.069 | 0.074 | 0.349 |  | -0.073 | 0.073 | 0.320 |
| rs87-1 | 0.028 | 0.037 | 0.448 |  | 0.033 | 0.037 | 0.377 |  | 0.054 | 0.037 | 0.143 |
| rs868127-2 | 0.104 | 0.068 | 0.126 |  | 0.119 | 0.069 | 0.082 |  | 0.024 | 0.068 | 0.727 |
| rs868127-1 | 0.007 | 0.037 | 0.860 |  | -0.014 | 0.037 | 0.706 |  | 0.026 | 0.037 | 0.485 |
| rs865438-2 | -0.080 | 0.095 | 0.399 |  | 0.041 | 0.095 | 0.669 |  | 0.054 | 0.095 | 0.569 |
| rs865438-1 | -0.066 | 0.098 | 0.497 |  | 0.037 | 0.098 | 0.708 |  | 0.008 | 0.098 | 0.936 |
| rs856999-2 | 0.040 | 0.055 | 0.466 |  | 0.054 | 0.055 | 0.332 |  | 0.080 | 0.055 | 0.146 |
| rs856999-1 | 0.054 | 0.052 | 0.305 |  | 0.081 | 0.052 | 0.123 |  | 0.067 | 0.052 | 0.200 |
| rs851320-2 | -0.082 | 0.071 | 0.251 |  | -0.094 | 0.071 | 0.186 |  | 0.043 | 0.071 | 0.542 |
| rs851320-1 | -0.059 | 0.038 | 0.113 |  | -0.071 | 0.038 | 0.059 |  | -0.007 | 0.038 | 0.849 |
| rs847147-2 | 0.005 | 0.070 | 0.946 |  | -0.061 | 0.071 | 0.391 |  | -0.083 | 0.070 | 0.237 |
| rs847147-1 | -0.028 | 0.036 | 0.444 |  | -0.021 | 0.036 | 0.562 |  | -0.021 | 0.036 | 0.566 |
| rs833823-2 | 0.055 | 0.261 | 0.835 |  | 0.021 | 0.263 | 0.937 |  | 0.247 | 0.261 | 0.343 |
| rs833823-1 | 0.001 | 0.266 | 0.997 |  | 0.009 | 0.268 | 0.974 |  | 0.236 | 0.266 | 0.375 |
| rs825453-2 | -0.097 | 0.050 | 0.052 |  | -0.104 | 0.050 | 0.039 |  | -0.053 | 0.050 | 0.289 |
| rs825453-1 | -0.083 | 0.048 | 0.081 |  | -0.085 | 0.048 | 0.075 |  | -0.066 | 0.048 | 0.167 |
| rs8192803-2 | -0.030 | 0.049 | 0.548 |  | -0.021 | 0.049 | 0.676 |  | -0.035 | 0.049 | 0.473 |
| rs8192803-1 | -0.081 | 0.046 | 0.081 |  | -0.057 | 0.047 | 0.219 |  | -0.044 | 0.046 | 0.344 |
| rs8134775-2 | -0.067 | 0.084 | 0.430 |  | -0.127 | 0.085 | 0.135 |  | -0.019 | 0.084 | 0.823 |
| rs8134775-1 | -0.057 | 0.085 | 0.498 |  | -0.095 | 0.085 | 0.265 |  | -0.042 | 0.084 | 0.622 |
| rs8132680-2 | -0.015 | 0.069 | 0.831 |  | -0.018 | 0.069 | 0.797 |  | 0.037 | 0.069 | 0.587 |
| rs8132680-1 | -0.029 | 0.070 | 0.684 |  | -0.059 | 0.071 | 0.404 |  | 0.046 | 0.070 | 0.515 |
| rs8121146-2 | -0.015 | 0.049 | 0.764 |  | 0.004 | 0.049 | 0.941 |  | -0.033 | 0.049 | 0.505 |
| rs8121146-1 | -0.052 | 0.043 | 0.232 |  | -0.021 | 0.043 | 0.629 |  | -0.043 | 0.043 | 0.318 |
| rs8109532-2 | 0.021 | 0.057 | 0.714 |  | 0.076 | 0.058 | 0.189 |  | 0.002 | 0.057 | 0.973 |
| rs8109532-1 | 0.067 | 0.037 | 0.070 |  | 0.058 | 0.037 | 0.118 |  | 0.040 | 0.037 | 0.281 |
| rs8104679-2 | 0.017 | 0.069 | 0.811 |  | 0.057 | 0.070 | 0.410 |  | -0.042 | 0.069 | 0.545 |
| rs8104679-1 | -0.039 | 0.037 | 0.291 |  | -0.014 | 0.037 | 0.698 |  | -0.023 | 0.037 | 0.533 |
| rs8096658-2 | -0.002 | 0.049 | 0.964 |  | 0.016 | 0.049 | 0.745 |  | 0.007 | 0.049 | 0.884 |
| rs8096658-1 | 0.029 | 0.042 | 0.482 |  | 0.047 | 0.042 | 0.258 |  | 0.034 | 0.042 | 0.408 |
| rs8095921-2 | 0.098 | 0.081 | 0.224 |  | 0.192 | 0.081 | 0.017 |  | 0.163 | 0.080 | 0.043 |
| rs8095921-1 | 0.059 | 0.084 | 0.478 |  | 0.151 | 0.084 | 0.073 |  | 0.136 | 0.083 | 0.104 |
| rs8070737-2 | -0.099 | 0.104 | 0.341 |  | -0.148 | 0.104 | 0.155 |  | -0.037 | 0.104 | 0.722 |
| rs8070737-1 | -0.070 | 0.106 | 0.508 |  | -0.111 | 0.107 | 0.300 |  | -0.005 | 0.106 | 0.963 |
| rs8068234-2 | 0.609 | 1.180 | 0.606 |  | 0.501 | 1.185 | 0.673 |  | 3.018 | 1.176 | 0.010 |
| rs8068234-1 | 0.519 | 1.184 | 0.661 |  | 0.384 | 1.190 | 0.747 |  | 3.051 | 1.180 | 0.010 |
| rs8066620-2 | -0.018 | 0.061 | 0.772 |  | -0.014 | 0.061 | 0.820 |  | -0.004 | 0.061 | 0.954 |
| rs8066620-1 | -0.028 | 0.036 | 0.433 |  | -0.019 | 0.036 | 0.585 |  | -0.004 | 0.035 | 0.914 |
| rs8063057-2 | 0.028 | 0.052 | 0.586 |  | -0.030 | 0.052 | 0.565 |  | 0.043 | 0.051 | 0.405 |
| rs8063057-1 | 0.039 | 0.050 | 0.438 |  | -0.002 | 0.051 | 0.975 |  | 0.083 | 0.050 | 0.097 |
| rs805770-2 | -0.054 | 0.050 | 0.279 |  | -0.002 | 0.051 | 0.972 |  | 0.040 | 0.050 | 0.421 |
| rs805770-1 | -0.011 | 0.047 | 0.812 |  | -0.004 | 0.047 | 0.933 |  | 0.085 | 0.047 | 0.070 |
| rs8052826-2 | 0.158 | 0.087 | 0.070 |  | 0.165 | 0.088 | 0.060 |  | 0.159 | 0.087 | 0.069 |
| rs8052826-1 | 0.192 | 0.090 | 0.032 |  | 0.221 | 0.090 | 0.014 |  | 0.126 | 0.090 | 0.159 |
| rs8045421-2 | -0.049 | 0.075 | 0.514 |  | -0.058 | 0.076 | 0.440 |  | -0.049 | 0.075 | 0.514 |
| rs8045421-1 | 0.029 | 0.077 | 0.707 |  | 0.021 | 0.077 | 0.788 |  | -0.021 | 0.077 | 0.783 |
| rs8038591-2 | 0.045 | 0.150 | 0.767 |  | 0.081 | 0.151 | 0.594 |  | 0.035 | 0.150 | 0.814 |
| rs8038591-1 | -0.077 | 0.044 | 0.084 |  | -0.083 | 0.045 | 0.062 |  | -0.091 | 0.044 | 0.040 |
| rs80222069-2 | 0.024 | 0.181 | 0.894 |  | 0.068 | 0.181 | 0.708 |  | 0.074 | 0.180 | 0.682 |
| rs80222069-1 | -0.045 | 0.045 | 0.310 |  | 0.002 | 0.045 | 0.971 |  | -0.013 | 0.045 | 0.766 |
| rs80040580-2 | -0.141 | 0.196 | 0.471 |  | -0.138 | 0.197 | 0.482 |  | -0.130 | 0.195 | 0.506 |
| rs80040580-1 | -0.034 | 0.051 | 0.507 |  | -0.043 | 0.051 | 0.399 |  | 0.010 | 0.051 | 0.839 |
| rs8002850-2 | 0.058 | 0.060 | 0.333 |  | 0.009 | 0.060 | 0.882 |  | -0.053 | 0.060 | 0.379 |
| rs8002850-1 | -0.039 | 0.036 | 0.275 |  | -0.070 | 0.036 | 0.053 |  | -0.032 | 0.036 | 0.371 |
| rs79999320-2 | -0.107 | 0.209 | 0.607 |  | 0.116 | 0.210 | 0.580 |  | -0.127 | 0.211 | 0.547 |
| rs79999320-1 | -0.124 | 0.214 | 0.561 |  | 0.125 | 0.215 | 0.559 |  | -0.165 | 0.216 | 0.445 |
| rs7991314-2 | 0.029 | 0.053 | 0.583 |  | 0.038 | 0.053 | 0.475 |  | 0.066 | 0.053 | 0.210 |
| rs7991314-1 | -0.004 | 0.036 | 0.918 |  | 0.013 | 0.037 | 0.731 |  | 0.012 | 0.036 | 0.735 |
| rs798545-2 | -0.093 | 0.067 | 0.163 |  | -0.024 | 0.067 | 0.719 |  | -0.003 | 0.066 | 0.958 |
| rs798545-1 | -0.078 | 0.067 | 0.250 |  | -0.026 | 0.068 | 0.702 |  | 0.037 | 0.067 | 0.579 |
| rs7981875-2 | -0.108 | 0.175 | 0.538 |  | 0.017 | 0.176 | 0.922 |  | -0.024 | 0.174 | 0.889 |
| rs7981875-1 | -0.205 | 0.179 | 0.252 |  | -0.061 | 0.179 | 0.735 |  | -0.128 | 0.178 | 0.474 |
| rs7975791-2 | -0.002 | 0.427 | 0.996 |  | 0.057 | 0.428 | 0.893 |  | 0.084 | 0.425 | 0.843 |
| rs7975791-1 | 0.043 | 0.070 | 0.539 |  | 0.059 | 0.071 | 0.401 |  | 0.004 | 0.070 | 0.950 |
| rs7974900-2 | -0.083 | 0.066 | 0.210 |  | -0.080 | 0.067 | 0.228 |  | -0.201 | 0.066 | 0.002 |
| rs7974900-1 | -0.019 | 0.037 | 0.599 |  | 0.013 | 0.037 | 0.731 |  | -0.049 | 0.037 | 0.181 |
| rs79730878-2 | 0.079 | 0.082 | 0.336 |  | 0.117 | 0.083 | 0.156 |  | -0.050 | 0.082 | 0.545 |
| rs79730878-1 | 0.011 | 0.037 | 0.777 |  | 0.003 | 0.037 | 0.944 |  | -0.042 | 0.037 | 0.254 |
| rs79719017-2 | 0.056 | 0.069 | 0.416 |  | 0.072 | 0.070 | 0.302 |  | -0.047 | 0.069 | 0.495 |
| rs79719017-1 | 0.046 | 0.071 | 0.512 |  | 0.051 | 0.071 | 0.470 |  | -0.091 | 0.071 | 0.199 |
| rs79717953-2 | 1.176 | 1.240 | 0.343 |  | 1.057 | 1.246 | 0.397 |  | 1.522 | 1.236 | 0.218 |
| rs79717953-1 | -0.236 | 0.132 | 0.073 |  | -0.211 | 0.132 | 0.111 |  | -0.352 | 0.131 | 0.007 |
| rs7961920-2 | 0.130 | 0.102 | 0.205 |  | 0.124 | 0.103 | 0.228 |  | 0.107 | 0.102 | 0.297 |
| rs7961920-1 | -0.038 | 0.039 | 0.330 |  | -0.034 | 0.039 | 0.387 |  | -0.032 | 0.039 | 0.415 |
| rs79598313-2 | -0.513 | 0.615 | 0.405 |  | -0.415 | 0.618 | 0.502 |  | -0.899 | 0.613 | 0.143 |
| rs79598313-1 | -0.433 | 0.620 | 0.485 |  | -0.399 | 0.623 | 0.521 |  | -0.733 | 0.618 | 0.236 |
| rs7959604-2 | -0.117 | 0.161 | 0.467 |  | 0.021 | 0.161 | 0.898 |  | 0.099 | 0.160 | 0.538 |
| rs7959604-1 | -0.096 | 0.046 | 0.037 |  | -0.117 | 0.046 | 0.012 |  | -0.007 | 0.046 | 0.879 |
| rs7953929-2 | -0.027 | 0.083 | 0.747 |  | -0.131 | 0.083 | 0.114 |  | -0.108 | 0.083 | 0.192 |
| rs7953929-1 | -0.033 | 0.037 | 0.383 |  | -0.078 | 0.037 | 0.037 |  | -0.062 | 0.037 | 0.095 |
| rs7949048-2 | 0.046 | 0.054 | 0.399 |  | 0.078 | 0.054 | 0.154 |  | -0.063 | 0.054 | 0.243 |
| rs7949048-1 | 0.053 | 0.053 | 0.315 |  | 0.096 | 0.053 | 0.071 |  | -0.010 | 0.053 | 0.846 |
| rs7943117-2 | 0.065 | 0.153 | 0.672 |  | 0.177 | 0.154 | 0.249 |  | 0.143 | 0.153 | 0.348 |
| rs7943117-1 | -0.008 | 0.045 | 0.852 |  | -0.021 | 0.045 | 0.636 |  | -0.041 | 0.045 | 0.365 |
| rs79409705-2 | -0.018 | 0.440 | 0.967 |  | 0.032 | 0.441 | 0.943 |  | -0.085 | 0.438 | 0.846 |
| rs79409705-1 | 0.166 | 0.442 | 0.707 |  | 0.222 | 0.444 | 0.618 |  | -0.028 | 0.441 | 0.949 |
| rs7937689-2 | 0.071 | 0.085 | 0.399 |  | 0.063 | 0.085 | 0.462 |  | 0.108 | 0.084 | 0.202 |
| rs7937689-1 | 0.029 | 0.037 | 0.438 |  | 0.005 | 0.037 | 0.895 |  | 0.011 | 0.037 | 0.773 |
| rs79364962-2 | 0.314 | 0.280 | 0.263 |  | 0.267 | 0.282 | 0.343 |  | -0.190 | 0.279 | 0.497 |
| rs79364962-1 | -0.012 | 0.055 | 0.831 |  | 0.037 | 0.056 | 0.511 |  | 0.001 | 0.055 | 0.990 |
| rs7931899-2 | 0.029 | 0.047 | 0.535 |  | 0.051 | 0.047 | 0.284 |  | -0.049 | 0.047 | 0.294 |
| rs7931899-1 | -0.015 | 0.043 | 0.736 |  | 0.003 | 0.044 | 0.943 |  | -0.056 | 0.043 | 0.197 |
| rs79163323-2 | -0.012 | 0.451 | 0.979 |  | -0.004 | 0.453 | 0.993 |  | -0.618 | 0.449 | 0.169 |
| rs79163323-1 | 0.014 | 0.453 | 0.975 |  | 0.054 | 0.455 | 0.906 |  | -0.577 | 0.452 | 0.201 |
| rs79028154-2 | -0.858 | 0.402 | 0.033 |  | -1.120 | 0.404 | 0.006 |  | -1.102 | 0.401 | 0.006 |
| rs79028154-1 | -0.773 | 0.406 | 0.057 |  | -1.040 | 0.408 | 0.011 |  | -1.022 | 0.404 | 0.012 |
| rs7902460-2 | 0.024 | 0.052 | 0.643 |  | 0.062 | 0.053 | 0.240 |  | -0.038 | 0.052 | 0.467 |
| rs7902460-1 | -0.007 | 0.038 | 0.851 |  | 0.014 | 0.038 | 0.712 |  | -0.075 | 0.038 | 0.048 |
| rs79016257-2 | -0.271 | 1.210 | 0.823 |  | -0.272 | 1.216 | 0.823 |  | 1.488 | 1.206 | 0.217 |
| rs79016257-1 | -0.233 | 1.213 | 0.848 |  | -0.274 | 1.218 | 0.822 |  | 1.603 | 1.209 | 0.185 |
| rs7893940-2 | 0.006 | 0.057 | 0.912 |  | 0.023 | 0.058 | 0.692 |  | -0.013 | 0.057 | 0.823 |
| rs7893940-1 | 0.018 | 0.036 | 0.629 |  | 0.030 | 0.036 | 0.405 |  | -0.019 | 0.036 | 0.597 |
| rs78817479-2 | 0.156 | 0.213 | 0.465 |  | 0.219 | 0.214 | 0.307 |  | 0.232 | 0.213 | 0.276 |
| rs78817479-1 | 0.209 | 0.217 | 0.335 |  | 0.257 | 0.218 | 0.239 |  | 0.339 | 0.217 | 0.118 |
| rs78733883-2 | -0.149 | 0.464 | 0.749 |  | -0.216 | 0.466 | 0.643 |  | -0.089 | 0.463 | 0.848 |
| rs78733883-1 | -0.047 | 0.467 | 0.920 |  | -0.107 | 0.469 | 0.820 |  | 0.020 | 0.465 | 0.965 |
| rs7866254-2 | 0.106 | 0.082 | 0.198 |  | 0.122 | 0.082 | 0.140 |  | -0.013 | 0.082 | 0.878 |
| rs7866254-1 | 0.074 | 0.084 | 0.383 |  | 0.095 | 0.085 | 0.265 |  | -0.025 | 0.084 | 0.764 |
| rs7866211-2 | 0.112 | 0.062 | 0.072 |  | 0.121 | 0.063 | 0.054 |  | 0.049 | 0.062 | 0.428 |
| rs7866211-1 | 0.023 | 0.036 | 0.532 |  | 0.045 | 0.037 | 0.218 |  | 0.049 | 0.036 | 0.177 |
| rs785836-2 | -0.051 | 0.055 | 0.358 |  | -0.048 | 0.055 | 0.389 |  | 0.030 | 0.055 | 0.582 |
| rs785836-1 | -0.111 | 0.055 | 0.044 |  | -0.119 | 0.055 | 0.033 |  | 0.008 | 0.055 | 0.883 |
| rs78520297-2 | -0.160 | 0.124 | 0.197 |  | -0.016 | 0.125 | 0.896 |  | 0.055 | 0.124 | 0.659 |
| rs78520297-1 | -0.115 | 0.041 | 0.005 |  | -0.080 | 0.041 | 0.052 |  | -0.095 | 0.041 | 0.019 |
| rs78438678-2 | -0.110 | 0.217 | 0.613 |  | -0.028 | 0.218 | 0.898 |  | -0.149 | 0.217 | 0.491 |
| rs78438678-1 | -0.111 | 0.056 | 0.046 |  | -0.062 | 0.056 | 0.267 |  | -0.107 | 0.056 | 0.055 |
| rs78432519-2 | 0.086 | 0.132 | 0.516 |  | 0.139 | 0.133 | 0.296 |  | 0.046 | 0.132 | 0.726 |
| rs78432519-1 | -0.048 | 0.042 | 0.249 |  | -0.034 | 0.042 | 0.417 |  | -0.052 | 0.042 | 0.209 |
| rs7830123-2 | 0.081 | 0.077 | 0.290 |  | 0.024 | 0.077 | 0.753 |  | 0.035 | 0.077 | 0.646 |
| rs7830123-1 | -0.014 | 0.036 | 0.690 |  | -0.030 | 0.036 | 0.402 |  | 0.030 | 0.036 | 0.411 |
| rs78252812-2 | -0.457 | 0.694 | 0.510 |  | 0.015 | 0.697 | 0.983 |  | -0.760 | 0.692 | 0.272 |
| rs78252812-1 | -0.717 | 0.696 | 0.303 |  | -0.140 | 0.699 | 0.841 |  | -1.009 | 0.694 | 0.146 |
| rs7820881-2 | -0.100 | 0.056 | 0.075 |  | -0.105 | 0.056 | 0.063 |  | -0.072 | 0.056 | 0.201 |
| rs7820881-1 | -0.106 | 0.055 | 0.053 |  | -0.064 | 0.055 | 0.243 |  | -0.033 | 0.055 | 0.552 |
| rs7815105-2 | 0.016 | 0.052 | 0.759 |  | 0.010 | 0.052 | 0.842 |  | -0.043 | 0.051 | 0.398 |
| rs7815105-1 | 0.021 | 0.050 | 0.667 |  | 0.019 | 0.050 | 0.710 |  | 0.015 | 0.050 | 0.754 |
| rs78150433-2 | 1.234 | 0.485 | 0.011 |  | 1.399 | 0.487 | 0.004 |  | 0.074 | 0.483 | 0.878 |
| rs78150433-1 | 1.225 | 0.491 | 0.013 |  | 1.403 | 0.493 | 0.005 |  | 0.123 | 0.490 | 0.801 |
| rs7814941-2 | 0.008 | 0.074 | 0.917 |  | -0.037 | 0.074 | 0.616 |  | 0.000 | 0.074 | 0.997 |
| rs7814941-1 | 0.021 | 0.074 | 0.778 |  | -0.029 | 0.074 | 0.693 |  | 0.035 | 0.074 | 0.632 |
| rs7812039-2 | -0.057 | 0.082 | 0.484 |  | -0.020 | 0.082 | 0.809 |  | 0.008 | 0.082 | 0.921 |
| rs7812039-1 | -0.042 | 0.082 | 0.611 |  | -0.008 | 0.083 | 0.921 |  | 0.049 | 0.082 | 0.549 |
| rs781142826-1 | -0.034 | 0.595 | 0.954 |  | 0.209 | 0.598 | 0.727 |  | -0.004 | 0.593 | 0.995 |
| rs78034425-2 | -0.065 | 0.161 | 0.687 |  | -0.113 | 0.162 | 0.486 |  | 0.049 | 0.161 | 0.760 |
| rs78034425-1 | -0.083 | 0.164 | 0.614 |  | -0.094 | 0.165 | 0.570 |  | -0.001 | 0.164 | 0.993 |
| rs78015143-2 | 0.022 | 0.214 | 0.919 |  | 0.135 | 0.215 | 0.531 |  | -0.152 | 0.214 | 0.478 |
| rs78015143-1 | -0.023 | 0.218 | 0.915 |  | 0.122 | 0.219 | 0.576 |  | -0.196 | 0.217 | 0.368 |
| rs7789097-2 | 0.062 | 0.123 | 0.614 |  | 0.072 | 0.123 | 0.558 |  | 0.002 | 0.122 | 0.989 |
| rs7789097-1 | -0.003 | 0.040 | 0.932 |  | 0.003 | 0.041 | 0.931 |  | 0.006 | 0.040 | 0.880 |
| rs7781279-2 | -0.134 | 0.067 | 0.047 |  | -0.105 | 0.068 | 0.121 |  | -0.176 | 0.067 | 0.009 |
| rs7781279-1 | 0.007 | 0.036 | 0.853 |  | 0.031 | 0.036 | 0.383 |  | 0.011 | 0.036 | 0.757 |
| rs7778451-2 | 0.019 | 0.054 | 0.724 |  | 0.020 | 0.055 | 0.717 |  | 0.013 | 0.054 | 0.808 |
| rs7778451-1 | -0.035 | 0.037 | 0.349 |  | -0.059 | 0.037 | 0.113 |  | -0.027 | 0.037 | 0.460 |
| rs77718124-2 | -0.238 | 0.293 | 0.418 |  | 0.050 | 0.294 | 0.865 |  | 0.053 | 0.292 | 0.857 |
| rs77718124-1 | -0.091 | 0.297 | 0.758 |  | 0.210 | 0.298 | 0.482 |  | 0.107 | 0.296 | 0.719 |
| rs7762532-2 | 0.095 | 0.081 | 0.239 |  | 0.124 | 0.081 | 0.128 |  | 0.142 | 0.081 | 0.079 |
| rs7762532-1 | 0.057 | 0.083 | 0.492 |  | 0.052 | 0.083 | 0.532 |  | 0.045 | 0.083 | 0.587 |
| rs77606322-2 | 0.003 | 0.341 | 0.993 |  | 0.073 | 0.343 | 0.832 |  | -0.200 | 0.340 | 0.556 |
| rs77606322-1 | -0.014 | 0.345 | 0.967 |  | 0.008 | 0.346 | 0.983 |  | -0.254 | 0.344 | 0.460 |
| rs77553148-2 | 0.488 | 0.220 | 0.026 |  | 0.460 | 0.220 | 0.037 |  | 0.251 | 0.223 | 0.259 |
| rs77553148-1 | 0.585 | 0.222 | 0.009 |  | 0.568 | 0.223 | 0.011 |  | 0.287 | 0.226 | 0.203 |
| rs77495054-2 | -0.225 | 0.486 | 0.643 |  | 0.067 | 0.488 | 0.891 |  | -0.782 | 0.484 | 0.106 |
| rs77495054-1 | -0.181 | 0.492 | 0.713 |  | 0.118 | 0.494 | 0.812 |  | -0.778 | 0.490 | 0.113 |
| rs7747253-2 | 0.017 | 0.048 | 0.720 |  | 0.020 | 0.048 | 0.683 |  | -0.005 | 0.048 | 0.916 |
| rs7747253-1 | -0.025 | 0.044 | 0.567 |  | -0.045 | 0.044 | 0.310 |  | 0.006 | 0.044 | 0.900 |
| rs77431781-2 | 0.088 | 0.242 | 0.718 |  | -0.121 | 0.244 | 0.618 |  | -0.360 | 0.242 | 0.137 |
| rs77431781-1 | -0.118 | 0.051 | 0.020 |  | -0.098 | 0.051 | 0.057 |  | 0.004 | 0.051 | 0.943 |
| rs77420750-2 | 0.058 | 0.063 | 0.351 |  | 0.040 | 0.063 | 0.522 |  | -0.091 | 0.062 | 0.145 |
| rs77420750-1 | 0.035 | 0.064 | 0.589 |  | 0.004 | 0.064 | 0.945 |  | -0.083 | 0.064 | 0.193 |
| rs77392239-2 | -0.052 | 0.173 | 0.763 |  | -0.025 | 0.174 | 0.884 |  | 0.060 | 0.173 | 0.727 |
| rs77392239-1 | 0.014 | 0.177 | 0.935 |  | 0.039 | 0.177 | 0.826 |  | 0.094 | 0.176 | 0.593 |
| rs772843886-1 | -0.391 | 0.837 | 0.640 |  | -0.803 | 0.841 | 0.340 |  | -0.036 | 0.834 | 0.966 |
| rs77216612-2 | 0.044 | 0.064 | 0.495 |  | 0.043 | 0.065 | 0.504 |  | 0.050 | 0.064 | 0.439 |
| rs77216612-1 | 0.049 | 0.065 | 0.449 |  | 0.058 | 0.065 | 0.374 |  | 0.060 | 0.065 | 0.358 |
| rs7703751-2 | 0.017 | 0.073 | 0.822 |  | -0.063 | 0.074 | 0.394 |  | -0.001 | 0.073 | 0.992 |
| rs7703751-1 | -0.058 | 0.036 | 0.104 |  | -0.064 | 0.036 | 0.076 |  | 0.010 | 0.036 | 0.784 |
| rs76990766-2 | -0.117 | 0.433 | 0.788 |  | -0.190 | 0.435 | 0.663 |  | 0.140 | 0.432 | 0.746 |
| rs76990766-1 | -0.064 | 0.438 | 0.884 |  | -0.141 | 0.440 | 0.749 |  | 0.108 | 0.436 | 0.805 |
| rs76983463-2 | 0.130 | 0.492 | 0.792 |  | 0.237 | 0.494 | 0.631 |  | -0.121 | 0.490 | 0.805 |
| rs76983463-1 | 0.127 | 0.496 | 0.799 |  | 0.233 | 0.498 | 0.640 |  | -0.192 | 0.495 | 0.698 |
| rs7694707-2 | 0.063 | 0.055 | 0.245 |  | 0.076 | 0.055 | 0.164 |  | 0.085 | 0.054 | 0.118 |
| rs7694707-1 | 0.048 | 0.052 | 0.362 |  | 0.089 | 0.053 | 0.093 |  | 0.075 | 0.052 | 0.152 |
| rs76895963-2 | 0.626 | 0.851 | 0.463 |  | 0.476 | 0.855 | 0.578 |  | 0.472 | 0.849 | 0.578 |
| rs76895963-1 | -0.086 | 0.095 | 0.367 |  | -0.103 | 0.096 | 0.281 |  | -0.014 | 0.095 | 0.881 |
| rs76833657-2 | -0.459 | 0.188 | 0.015 |  | -0.467 | 0.189 | 0.013 |  | -0.078 | 0.187 | 0.675 |
| rs76833657-1 | -0.054 | 0.051 | 0.287 |  | -0.053 | 0.051 | 0.302 |  | -0.074 | 0.051 | 0.147 |
| rs76819935-2 | -0.170 | 0.433 | 0.694 |  | -0.389 | 0.435 | 0.371 |  | -0.213 | 0.431 | 0.622 |
| rs76819935-1 | -0.340 | 0.436 | 0.436 |  | -0.578 | 0.438 | 0.187 |  | -0.301 | 0.435 | 0.489 |
| rs7681056-2 | -0.073 | 0.061 | 0.227 |  | -0.051 | 0.061 | 0.405 |  | -0.039 | 0.060 | 0.521 |
| rs7681056-1 | -0.064 | 0.061 | 0.295 |  | -0.058 | 0.061 | 0.344 |  | -0.066 | 0.061 | 0.279 |
| rs7679094-2 | 0.002 | 0.050 | 0.966 |  | -0.014 | 0.050 | 0.776 |  | -0.096 | 0.050 | 0.053 |
| rs7679094-1 | -0.002 | 0.039 | 0.967 |  | -0.023 | 0.039 | 0.561 |  | -0.029 | 0.039 | 0.448 |
| rs767855-2 | 0.087 | 0.059 | 0.136 |  | 0.120 | 0.059 | 0.042 |  | 0.121 | 0.058 | 0.038 |
| rs767855-1 | 0.087 | 0.057 | 0.129 |  | 0.102 | 0.058 | 0.078 |  | 0.098 | 0.057 | 0.088 |
| rs76742266-2 | -0.289 | 0.203 | 0.154 |  | -0.325 | 0.204 | 0.111 |  | -0.589 | 0.202 | 0.004 |
| rs76742266-1 | -0.296 | 0.203 | 0.146 |  | -0.364 | 0.204 | 0.075 |  | -0.632 | 0.203 | 0.002 |
| rs76504223-2 | 0.219 | 0.192 | 0.254 |  | 0.219 | 0.193 | 0.254 |  | -0.027 | 0.191 | 0.886 |
| rs76504223-1 | -0.019 | 0.047 | 0.691 |  | -0.024 | 0.047 | 0.606 |  | 0.013 | 0.047 | 0.774 |
| rs764396-2 | -0.058 | 0.049 | 0.233 |  | -0.036 | 0.049 | 0.462 |  | -0.124 | 0.049 | 0.011 |
| rs764396-1 | -0.033 | 0.043 | 0.449 |  | -0.012 | 0.044 | 0.787 |  | -0.072 | 0.043 | 0.098 |
| rs7633119-2 | -0.075 | 0.061 | 0.217 |  | -0.035 | 0.061 | 0.567 |  | 0.048 | 0.061 | 0.436 |
| rs7633119-1 | -0.074 | 0.037 | 0.044 |  | -0.027 | 0.037 | 0.461 |  | -0.009 | 0.037 | 0.797 |
| rs7632937-2 | -0.230 | 0.145 | 0.112 |  | -0.164 | 0.145 | 0.260 |  | -0.178 | 0.144 | 0.217 |
| rs7632937-1 | -0.213 | 0.145 | 0.141 |  | -0.157 | 0.145 | 0.279 |  | -0.214 | 0.144 | 0.137 |
| rs76287541-2 | -0.140 | 0.084 | 0.095 |  | -0.069 | 0.084 | 0.412 |  | -0.084 | 0.083 | 0.314 |
| rs76287541-1 | -0.105 | 0.085 | 0.217 |  | -0.036 | 0.085 | 0.676 |  | -0.142 | 0.085 | 0.093 |
| rs76210166-2 | -0.059 | 0.090 | 0.511 |  | 0.026 | 0.090 | 0.771 |  | 0.050 | 0.089 | 0.573 |
| rs76210166-1 | -0.035 | 0.038 | 0.349 |  | -0.055 | 0.038 | 0.148 |  | 0.023 | 0.037 | 0.535 |
| rs760938-2 | -0.054 | 0.048 | 0.259 |  | -0.045 | 0.048 | 0.345 |  | -0.062 | 0.048 | 0.191 |
| rs760938-1 | 0.014 | 0.043 | 0.735 |  | 0.000 | 0.043 | 0.995 |  | -0.020 | 0.043 | 0.642 |
| rs75983310-2 | 0.054 | 0.074 | 0.472 |  | 0.065 | 0.075 | 0.386 |  | 0.085 | 0.074 | 0.249 |
| rs75983310-1 | -0.040 | 0.036 | 0.269 |  | -0.032 | 0.036 | 0.383 |  | 0.026 | 0.036 | 0.474 |
| rs75937733-2 | 0.058 | 0.207 | 0.777 |  | 0.082 | 0.208 | 0.694 |  | 0.148 | 0.206 | 0.474 |
| rs75937733-1 | 0.081 | 0.210 | 0.700 |  | 0.116 | 0.211 | 0.584 |  | 0.270 | 0.210 | 0.198 |
| rs7584710-2 | -0.058 | 0.053 | 0.279 |  | -0.025 | 0.054 | 0.635 |  | 0.007 | 0.053 | 0.888 |
| rs7584710-1 | -0.133 | 0.053 | 0.012 |  | -0.091 | 0.053 | 0.086 |  | -0.040 | 0.053 | 0.449 |
| rs757980-2 | 0.101 | 0.083 | 0.219 |  | 0.089 | 0.083 | 0.284 |  | -0.085 | 0.083 | 0.301 |
| rs757980-1 | 0.117 | 0.084 | 0.166 |  | 0.126 | 0.085 | 0.136 |  | -0.106 | 0.084 | 0.209 |
| rs7573217-2 | -0.072 | 0.179 | 0.689 |  | -0.179 | 0.180 | 0.319 |  | -0.127 | 0.179 | 0.478 |
| rs7573217-1 | -0.039 | 0.046 | 0.388 |  | 0.020 | 0.046 | 0.663 |  | -0.019 | 0.045 | 0.684 |
| rs7571898-2 | 0.153 | 0.074 | 0.038 |  | 0.191 | 0.074 | 0.010 |  | 0.053 | 0.074 | 0.474 |
| rs7571898-1 | 0.071 | 0.037 | 0.053 |  | 0.063 | 0.037 | 0.085 |  | 0.008 | 0.037 | 0.819 |
| rs75660521-2 | -0.154 | 0.206 | 0.454 |  | -0.128 | 0.207 | 0.536 |  | -0.009 | 0.206 | 0.964 |
| rs75660521-1 | 0.061 | 0.049 | 0.217 |  | 0.060 | 0.049 | 0.223 |  | 0.126 | 0.049 | 0.010 |
| rs75587692-1 | 0.013 | 0.113 | 0.905 |  | 0.006 | 0.114 | 0.961 |  | 0.107 | 0.113 | 0.344 |
| rs7554551-2 | -0.129 | 0.061 | 0.033 |  | -0.126 | 0.061 | 0.038 |  | -0.184 | 0.060 | 0.002 |
| rs7554551-1 | -0.108 | 0.057 | 0.060 |  | -0.109 | 0.057 | 0.057 |  | -0.124 | 0.057 | 0.030 |
| rs75475627-2 | -0.212 | 0.180 | 0.238 |  | -0.189 | 0.181 | 0.295 |  | -0.006 | 0.179 | 0.975 |
| rs75475627-1 | -0.112 | 0.057 | 0.050 |  | -0.087 | 0.057 | 0.129 |  | -0.061 | 0.057 | 0.283 |
| rs7546500-2 | 0.046 | 0.061 | 0.447 |  | 0.052 | 0.061 | 0.397 |  | 0.021 | 0.061 | 0.736 |
| rs7546500-1 | 0.023 | 0.061 | 0.704 |  | 0.029 | 0.062 | 0.643 |  | 0.021 | 0.061 | 0.735 |
| rs75439322-2 | 0.306 | 0.208 | 0.141 |  | 0.093 | 0.209 | 0.657 |  | 0.148 | 0.208 | 0.475 |
| rs75439322-1 | 0.300 | 0.211 | 0.155 |  | 0.076 | 0.212 | 0.721 |  | 0.140 | 0.211 | 0.508 |
| rs7527300-2 | 0.026 | 0.051 | 0.608 |  | 0.019 | 0.051 | 0.713 |  | 0.031 | 0.051 | 0.542 |
| rs7527300-1 | -0.026 | 0.037 | 0.476 |  | -0.022 | 0.037 | 0.558 |  | -0.005 | 0.037 | 0.900 |
| rs75230517-2 | 0.024 | 0.385 | 0.951 |  | -0.088 | 0.387 | 0.820 |  | -0.522 | 0.384 | 0.174 |
| rs75230517-1 | 0.016 | 0.058 | 0.785 |  | 0.043 | 0.058 | 0.460 |  | 0.037 | 0.058 | 0.518 |
| rs751979-2 | -0.002 | 0.055 | 0.968 |  | -0.006 | 0.055 | 0.907 |  | -0.050 | 0.055 | 0.363 |
| rs751979-1 | 0.022 | 0.054 | 0.690 |  | 0.001 | 0.054 | 0.979 |  | -0.044 | 0.054 | 0.416 |
| rs7516171-2 | 0.012 | 0.090 | 0.897 |  | -0.065 | 0.090 | 0.473 |  | 0.025 | 0.090 | 0.781 |
| rs7516171-1 | 0.038 | 0.092 | 0.684 |  | -0.063 | 0.093 | 0.499 |  | 0.070 | 0.092 | 0.444 |
| rs75147459-2 | -0.399 | 0.464 | 0.390 |  | -0.377 | 0.466 | 0.419 |  | 0.088 | 0.462 | 0.849 |
| rs75147459-1 | -0.557 | 0.468 | 0.234 |  | -0.522 | 0.470 | 0.267 |  | -0.032 | 0.466 | 0.946 |
| rs75077568-2 | -1.067 | 0.505 | 0.035 |  | -0.613 | 0.507 | 0.227 |  | -0.068 | 0.503 | 0.893 |
| rs75077568-1 | -1.229 | 0.512 | 0.017 |  | -0.840 | 0.515 | 0.103 |  | -0.278 | 0.511 | 0.586 |
| rs7504492-2 | -0.088 | 0.055 | 0.107 |  | -0.085 | 0.055 | 0.124 |  | 0.011 | 0.055 | 0.842 |
| rs7504492-1 | -0.047 | 0.053 | 0.378 |  | -0.041 | 0.053 | 0.440 |  | -0.030 | 0.053 | 0.567 |
| rs74910854-2 | 0.018 | 0.263 | 0.944 |  | -0.014 | 0.264 | 0.958 |  | -0.008 | 0.262 | 0.976 |
| rs74910854-1 | 0.021 | 0.052 | 0.680 |  | -0.009 | 0.052 | 0.859 |  | -0.025 | 0.052 | 0.629 |
| rs7488974-2 | -0.009 | 0.056 | 0.873 |  | 0.034 | 0.056 | 0.544 |  | -0.031 | 0.056 | 0.575 |
| rs7488974-1 | 0.019 | 0.051 | 0.712 |  | 0.052 | 0.051 | 0.307 |  | -0.045 | 0.051 | 0.370 |
| rs7484147-2 | -0.068 | 0.055 | 0.215 |  | -0.067 | 0.055 | 0.223 |  | 0.030 | 0.055 | 0.581 |
| rs7484147-1 | -0.071 | 0.051 | 0.164 |  | -0.038 | 0.051 | 0.454 |  | -0.030 | 0.051 | 0.552 |
| rs747091-2 | 0.002 | 0.050 | 0.975 |  | 0.030 | 0.050 | 0.545 |  | 0.063 | 0.050 | 0.200 |
| rs747091-1 | -0.003 | 0.039 | 0.938 |  | 0.034 | 0.039 | 0.379 |  | -0.006 | 0.039 | 0.880 |
| rs7444282-2 | -0.111 | 0.081 | 0.169 |  | -0.074 | 0.081 | 0.363 |  | -0.089 | 0.080 | 0.267 |
| rs7444282-1 | 0.035 | 0.036 | 0.329 |  | 0.031 | 0.036 | 0.387 |  | -0.014 | 0.036 | 0.692 |
| rs74394067-2 | 0.340 | 1.192 | 0.775 |  | 0.180 | 1.197 | 0.880 |  | 0.947 | 1.188 | 0.425 |
| rs74394067-1 | -0.057 | 0.094 | 0.542 |  | -0.084 | 0.094 | 0.373 |  | -0.155 | 0.093 | 0.098 |
| rs74331022-2 | 0.018 | 0.105 | 0.864 |  | -0.011 | 0.106 | 0.920 |  | 0.083 | 0.105 | 0.430 |
| rs74331022-1 | -0.066 | 0.041 | 0.105 |  | -0.059 | 0.041 | 0.150 |  | -0.050 | 0.040 | 0.220 |
| rs74119759-2 | 0.157 | 0.106 | 0.140 |  | 0.112 | 0.107 | 0.293 |  | 0.101 | 0.106 | 0.340 |
| rs74119759-1 | -0.043 | 0.039 | 0.264 |  | -0.045 | 0.039 | 0.245 |  | -0.072 | 0.039 | 0.063 |
| rs740753-2 | 0.118 | 0.179 | 0.512 |  | 0.137 | 0.180 | 0.445 |  | 0.018 | 0.179 | 0.918 |
| rs740753-1 | 0.172 | 0.183 | 0.349 |  | 0.175 | 0.184 | 0.342 |  | 0.136 | 0.183 | 0.457 |
| rs737534-2 | -0.021 | 0.081 | 0.791 |  | -0.015 | 0.082 | 0.854 |  | 0.057 | 0.081 | 0.481 |
| rs737534-1 | -0.011 | 0.038 | 0.761 |  | -0.003 | 0.038 | 0.932 |  | 0.024 | 0.038 | 0.521 |
| rs73479996-2 | 0.040 | 0.216 | 0.854 |  | -0.080 | 0.217 | 0.712 |  | 0.277 | 0.216 | 0.199 |
| rs73479996-1 | 0.021 | 0.051 | 0.685 |  | 0.042 | 0.052 | 0.414 |  | -0.002 | 0.051 | 0.971 |
| rs73388841-2 | 0.155 | 0.201 | 0.439 |  | 0.119 | 0.201 | 0.554 |  | 0.157 | 0.200 | 0.433 |
| rs73388841-1 | -0.028 | 0.054 | 0.610 |  | 0.012 | 0.055 | 0.828 |  | 0.033 | 0.054 | 0.543 |
| rs73260919-2 | -0.175 | 0.247 | 0.478 |  | -0.162 | 0.248 | 0.513 |  | -0.368 | 0.246 | 0.135 |
| rs73260919-1 | 0.046 | 0.055 | 0.398 |  | 0.055 | 0.055 | 0.312 |  | 0.004 | 0.054 | 0.940 |
| rs7324365-2 | -0.158 | 0.098 | 0.107 |  | -0.142 | 0.098 | 0.149 |  | -0.061 | 0.098 | 0.533 |
| rs7324365-1 | -0.157 | 0.100 | 0.116 |  | -0.164 | 0.100 | 0.102 |  | -0.070 | 0.100 | 0.482 |
| rs73238169-2 | -0.073 | 0.134 | 0.586 |  | -0.045 | 0.134 | 0.735 |  | 0.109 | 0.134 | 0.415 |
| rs73238169-1 | -0.086 | 0.041 | 0.035 |  | -0.058 | 0.041 | 0.151 |  | -0.026 | 0.041 | 0.526 |
| rs73212812-2 | -0.672 | 0.861 | 0.435 |  | -0.937 | 0.865 | 0.278 |  | -1.222 | 0.858 | 0.155 |
| rs73212812-1 | -0.615 | 0.863 | 0.476 |  | -0.851 | 0.867 | 0.326 |  | -1.272 | 0.860 | 0.139 |
| rs73203066-2 | -0.170 | 0.132 | 0.196 |  | -0.167 | 0.132 | 0.207 |  | -0.202 | 0.131 | 0.124 |
| rs73203066-1 | -0.176 | 0.135 | 0.193 |  | -0.177 | 0.136 | 0.193 |  | -0.204 | 0.135 | 0.130 |
| rs73156468-2 | 0.017 | 0.112 | 0.882 |  | 0.121 | 0.113 | 0.282 |  | -0.091 | 0.112 | 0.416 |
| rs73156468-1 | -0.061 | 0.040 | 0.126 |  | -0.054 | 0.040 | 0.175 |  | -0.026 | 0.040 | 0.519 |
| rs73091252-2 | -0.112 | 0.619 | 0.857 |  | 0.220 | 0.621 | 0.723 |  | 0.315 | 0.617 | 0.610 |
| rs73091252-1 | 0.074 | 0.624 | 0.906 |  | 0.332 | 0.626 | 0.596 |  | 0.420 | 0.622 | 0.500 |
| rs73082474-2 | 1.485 | 1.190 | 0.212 |  | 1.749 | 1.195 | 0.143 |  | 1.512 | 1.187 | 0.203 |
| rs73082474-1 | -0.020 | 0.090 | 0.821 |  | -0.021 | 0.090 | 0.819 |  | 0.080 | 0.090 | 0.372 |
| rs73066226-2 | -0.011 | 0.111 | 0.920 |  | -0.038 | 0.111 | 0.731 |  | -0.084 | 0.110 | 0.446 |
| rs73066226-1 | 0.011 | 0.113 | 0.921 |  | -0.018 | 0.114 | 0.877 |  | 0.000 | 0.113 | 0.998 |
| rs73029263-2 | 0.017 | 0.121 | 0.890 |  | -0.041 | 0.122 | 0.737 |  | 0.106 | 0.121 | 0.380 |
| rs73029263-1 | -0.043 | 0.041 | 0.297 |  | -0.032 | 0.041 | 0.435 |  | -0.013 | 0.041 | 0.745 |
| rs7301013-2 | 0.069 | 0.105 | 0.510 |  | 0.024 | 0.105 | 0.823 |  | -0.053 | 0.104 | 0.608 |
| rs7301013-1 | 0.085 | 0.107 | 0.432 |  | 0.056 | 0.108 | 0.604 |  | -0.027 | 0.107 | 0.798 |
| rs72945685-2 | -0.074 | 0.157 | 0.638 |  | -0.074 | 0.158 | 0.640 |  | -0.224 | 0.156 | 0.152 |
| rs72945685-1 | -0.111 | 0.160 | 0.490 |  | -0.125 | 0.161 | 0.438 |  | -0.294 | 0.160 | 0.066 |
| rs72912147-2 | 0.060 | 0.080 | 0.452 |  | -0.029 | 0.080 | 0.717 |  | 0.041 | 0.080 | 0.607 |
| rs72912147-1 | 0.028 | 0.082 | 0.733 |  | -0.078 | 0.082 | 0.341 |  | 0.021 | 0.082 | 0.797 |
| rs72874231-2 | -0.108 | 0.338 | 0.750 |  | -0.184 | 0.339 | 0.587 |  | -0.164 | 0.337 | 0.627 |
| rs72874231-1 | -0.104 | 0.343 | 0.762 |  | -0.158 | 0.345 | 0.646 |  | -0.088 | 0.342 | 0.797 |
| rs72868839-2 | -0.217 | 0.383 | 0.572 |  | -0.512 | 0.385 | 0.183 |  | 0.379 | 0.382 | 0.321 |
| rs72868839-1 | -0.187 | 0.387 | 0.628 |  | -0.493 | 0.389 | 0.204 |  | 0.289 | 0.385 | 0.454 |
| rs72840032-2 | -1.281 | 0.345 | 0.000 |  | -1.371 | 0.347 | 0.000 |  | -0.617 | 0.344 | 0.073 |
| rs72840032-1 | -1.176 | 0.350 | 0.001 |  | -1.264 | 0.351 | 0.000 |  | -0.547 | 0.349 | 0.117 |
| rs7283257-2 | -0.016 | 0.257 | 0.952 |  | 0.083 | 0.258 | 0.746 |  | -0.085 | 0.256 | 0.740 |
| rs7283257-1 | 0.004 | 0.260 | 0.987 |  | 0.153 | 0.261 | 0.558 |  | -0.054 | 0.259 | 0.836 |
| rs72813180-2 | -0.056 | 0.101 | 0.579 |  | -0.062 | 0.102 | 0.546 |  | -0.101 | 0.101 | 0.316 |
| rs72813180-1 | 0.009 | 0.040 | 0.831 |  | 0.017 | 0.040 | 0.678 |  | 0.029 | 0.040 | 0.470 |
| rs72805220-2 | 0.165 | 0.313 | 0.598 |  | 0.214 | 0.314 | 0.495 |  | -0.195 | 0.312 | 0.532 |
| rs72805220-1 | -0.006 | 0.055 | 0.917 |  | -0.032 | 0.055 | 0.560 |  | -0.032 | 0.055 | 0.559 |
| rs72801445-2 | -0.119 | 0.076 | 0.116 |  | -0.050 | 0.076 | 0.514 |  | -0.012 | 0.075 | 0.872 |
| rs72801445-1 | -0.126 | 0.078 | 0.104 |  | -0.044 | 0.078 | 0.576 |  | -0.076 | 0.077 | 0.325 |
| rs7274697-2 | -0.077 | 0.155 | 0.618 |  | -0.141 | 0.155 | 0.364 |  | -0.258 | 0.154 | 0.095 |
| rs7274697-1 | -0.155 | 0.156 | 0.323 |  | -0.236 | 0.157 | 0.133 |  | -0.252 | 0.156 | 0.105 |
| rs72726582-2 | -0.124 | 0.199 | 0.534 |  | -0.181 | 0.200 | 0.367 |  | -0.105 | 0.199 | 0.599 |
| rs72726582-1 | 0.031 | 0.051 | 0.544 |  | 0.048 | 0.051 | 0.347 |  | -0.087 | 0.051 | 0.088 |
| rs7270518-2 | 0.074 | 0.308 | 0.811 |  | -0.304 | 0.309 | 0.325 |  | -0.246 | 0.307 | 0.423 |
| rs7270518-1 | -0.004 | 0.311 | 0.989 |  | -0.351 | 0.313 | 0.262 |  | -0.314 | 0.310 | 0.312 |
| rs72703614-2 | -0.115 | 0.084 | 0.170 |  | -0.109 | 0.084 | 0.195 |  | -0.109 | 0.084 | 0.195 |
| rs72703614-1 | -0.086 | 0.086 | 0.319 |  | -0.094 | 0.086 | 0.275 |  | -0.119 | 0.086 | 0.167 |
| rs72692842-2 | -0.248 | 0.197 | 0.209 |  | -0.057 | 0.198 | 0.772 |  | -0.415 | 0.196 | 0.035 |
| rs72692842-1 | 0.017 | 0.046 | 0.710 |  | -0.002 | 0.047 | 0.964 |  | 0.047 | 0.046 | 0.310 |
| rs726857-2 | 0.047 | 0.057 | 0.405 |  | 0.040 | 0.057 | 0.482 |  | -0.015 | 0.057 | 0.794 |
| rs726857-1 | 0.037 | 0.053 | 0.484 |  | 0.020 | 0.053 | 0.712 |  | 0.007 | 0.053 | 0.891 |
| rs7267595-2 | -0.036 | 0.053 | 0.494 |  | -0.081 | 0.053 | 0.128 |  | -0.082 | 0.053 | 0.121 |
| rs7267595-1 | -0.021 | 0.044 | 0.627 |  | -0.085 | 0.044 | 0.053 |  | -0.072 | 0.043 | 0.099 |
| rs72656010-2 | -0.050 | 0.115 | 0.664 |  | -0.035 | 0.116 | 0.760 |  | 0.086 | 0.115 | 0.455 |
| rs72656010-1 | 0.010 | 0.118 | 0.931 |  | 0.010 | 0.118 | 0.931 |  | 0.121 | 0.118 | 0.305 |
| rs72644599-2 | 0.184 | 0.684 | 0.787 |  | 0.029 | 0.687 | 0.967 |  | 1.252 | 0.681 | 0.066 |
| rs72644599-1 | 0.072 | 0.076 | 0.343 |  | 0.099 | 0.076 | 0.194 |  | 0.063 | 0.076 | 0.404 |
| rs72643433-2 | -0.089 | 0.072 | 0.220 |  | -0.094 | 0.072 | 0.196 |  | -0.019 | 0.072 | 0.787 |
| rs72643433-1 | 0.009 | 0.037 | 0.809 |  | -0.023 | 0.037 | 0.537 |  | 0.028 | 0.037 | 0.458 |
| rs72640504-1 | 0.316 | 0.179 | 0.076 |  | 0.259 | 0.179 | 0.148 |  | 0.132 | 0.178 | 0.458 |
| rs72636758-2 | -0.012 | 0.143 | 0.931 |  | -0.006 | 0.144 | 0.969 |  | -0.104 | 0.143 | 0.468 |
| rs72636758-1 | -0.038 | 0.146 | 0.793 |  | -0.011 | 0.147 | 0.938 |  | -0.142 | 0.146 | 0.329 |
| rs72613886-2 | -0.198 | 0.121 | 0.103 |  | -0.191 | 0.122 | 0.116 |  | -0.081 | 0.121 | 0.500 |
| rs72613886-1 | -0.016 | 0.042 | 0.702 |  | -0.040 | 0.042 | 0.346 |  | -0.014 | 0.042 | 0.738 |
| rs7259714-2 | 0.015 | 0.051 | 0.774 |  | 0.030 | 0.051 | 0.551 |  | -0.020 | 0.050 | 0.686 |
| rs7259714-1 | 0.014 | 0.038 | 0.707 |  | 0.029 | 0.038 | 0.439 |  | -0.017 | 0.038 | 0.660 |
| rs7237942-2 | -0.044 | 0.085 | 0.607 |  | -0.053 | 0.085 | 0.534 |  | -0.046 | 0.085 | 0.590 |
| rs7237942-1 | -0.098 | 0.088 | 0.265 |  | -0.091 | 0.088 | 0.299 |  | -0.093 | 0.087 | 0.286 |
| rs7236090-2 | 0.087 | 0.049 | 0.078 |  | 0.099 | 0.050 | 0.047 |  | 0.083 | 0.049 | 0.091 |
| rs7236090-1 | 0.031 | 0.039 | 0.433 |  | 0.017 | 0.039 | 0.665 |  | 0.025 | 0.039 | 0.529 |
| rs722526-2 | -0.042 | 0.047 | 0.371 |  | 0.009 | 0.048 | 0.848 |  | -0.005 | 0.047 | 0.913 |
| rs722526-1 | -0.043 | 0.041 | 0.291 |  | 0.003 | 0.041 | 0.950 |  | -0.043 | 0.041 | 0.291 |
| rs7217523-2 | -0.296 | 0.317 | 0.351 |  | 0.072 | 0.318 | 0.822 |  | -0.128 | 0.316 | 0.686 |
| rs7217523-1 | -0.230 | 0.320 | 0.473 |  | 0.156 | 0.322 | 0.629 |  | -0.028 | 0.319 | 0.930 |
| rs7217502-2 | -0.202 | 0.054 | 0.000 |  | -0.126 | 0.055 | 0.021 |  | -0.104 | 0.054 | 0.055 |
| rs7217502-1 | -0.113 | 0.050 | 0.024 |  | -0.053 | 0.050 | 0.288 |  | -0.008 | 0.050 | 0.870 |
| rs7215879-2 | -0.131 | 0.121 | 0.280 |  | -0.130 | 0.122 | 0.283 |  | 0.009 | 0.121 | 0.938 |
| rs7215879-1 | -0.128 | 0.124 | 0.302 |  | -0.153 | 0.125 | 0.219 |  | -0.006 | 0.124 | 0.960 |
| rs7212578-2 | 0.102 | 0.087 | 0.241 |  | 0.062 | 0.087 | 0.478 |  | -0.024 | 0.087 | 0.782 |
| rs7212578-1 | -0.006 | 0.037 | 0.873 |  | -0.026 | 0.037 | 0.489 |  | -0.037 | 0.037 | 0.311 |
| rs7211043-2 | 0.078 | 0.066 | 0.241 |  | 0.066 | 0.067 | 0.321 |  | -0.007 | 0.066 | 0.910 |
| rs7211043-1 | 0.024 | 0.036 | 0.498 |  | 0.007 | 0.036 | 0.848 |  | -0.005 | 0.036 | 0.879 |
| rs7209460-2 | -0.098 | 0.062 | 0.110 |  | -0.192 | 0.062 | 0.002 |  | -0.080 | 0.061 | 0.195 |
| rs7209460-1 | -0.062 | 0.036 | 0.083 |  | -0.121 | 0.036 | 0.001 |  | -0.039 | 0.036 | 0.279 |
| rs7198843-2 | -0.021 | 0.050 | 0.666 |  | -0.015 | 0.050 | 0.760 |  | -0.094 | 0.049 | 0.057 |
| rs7198843-1 | -0.014 | 0.040 | 0.725 |  | -0.016 | 0.040 | 0.696 |  | -0.086 | 0.039 | 0.029 |
| rs7191269-2 | -0.105 | 0.050 | 0.038 |  | -0.162 | 0.051 | 0.001 |  | -0.050 | 0.050 | 0.321 |
| rs7191269-1 | -0.064 | 0.038 | 0.092 |  | -0.055 | 0.038 | 0.149 |  | -0.035 | 0.038 | 0.355 |
| rs7186061-2 | -0.150 | 0.123 | 0.223 |  | -0.106 | 0.123 | 0.390 |  | -0.194 | 0.123 | 0.114 |
| rs7186061-1 | -0.049 | 0.040 | 0.225 |  | -0.042 | 0.040 | 0.300 |  | 0.003 | 0.040 | 0.949 |
| rs7175531-2 | -0.123 | 0.062 | 0.047 |  | -0.190 | 0.062 | 0.002 |  | -0.135 | 0.062 | 0.029 |
| rs7175531-1 | -0.015 | 0.038 | 0.693 |  | -0.028 | 0.039 | 0.472 |  | -0.022 | 0.038 | 0.562 |
| rs7170637-2 | -0.122 | 0.091 | 0.183 |  | -0.135 | 0.092 | 0.142 |  | -0.112 | 0.091 | 0.219 |
| rs7170637-1 | -0.039 | 0.039 | 0.326 |  | -0.021 | 0.039 | 0.593 |  | -0.028 | 0.039 | 0.472 |
| rs7167692-2 | -0.512 | 0.210 | 0.015 |  | -0.332 | 0.211 | 0.116 |  | -0.177 | 0.209 | 0.397 |
| rs7167692-1 | -0.055 | 0.057 | 0.332 |  | -0.047 | 0.057 | 0.404 |  | -0.033 | 0.056 | 0.556 |
| rs71539572-2 | 0.063 | 0.081 | 0.435 |  | 0.023 | 0.082 | 0.775 |  | 0.006 | 0.081 | 0.941 |
| rs71539572-1 | 0.011 | 0.083 | 0.899 |  | -0.026 | 0.084 | 0.759 |  | -0.035 | 0.083 | 0.674 |
| rs7147775-2 | 0.008 | 0.048 | 0.864 |  | 0.039 | 0.049 | 0.419 |  | -0.005 | 0.048 | 0.916 |
| rs7147775-1 | -0.011 | 0.040 | 0.785 |  | -0.021 | 0.040 | 0.607 |  | -0.021 | 0.040 | 0.595 |
| rs7147577-2 | -0.095 | 0.054 | 0.081 |  | -0.085 | 0.054 | 0.121 |  | -0.058 | 0.054 | 0.287 |
| rs7147577-1 | -0.051 | 0.037 | 0.164 |  | -0.041 | 0.037 | 0.270 |  | 0.017 | 0.037 | 0.651 |
| rs7146983-2 | -0.123 | 0.153 | 0.419 |  | -0.097 | 0.153 | 0.525 |  | -0.029 | 0.152 | 0.848 |
| rs7146983-1 | -0.057 | 0.046 | 0.211 |  | 0.004 | 0.046 | 0.938 |  | 0.038 | 0.046 | 0.408 |
| rs71420186-2 | -0.315 | 0.255 | 0.217 |  | -0.195 | 0.256 | 0.446 |  | -0.227 | 0.254 | 0.372 |
| rs71420186-1 | -0.014 | 0.053 | 0.785 |  | -0.089 | 0.053 | 0.096 |  | 0.020 | 0.053 | 0.699 |
| rs71390846-2 | -0.202 | 0.093 | 0.030 |  | -0.128 | 0.094 | 0.171 |  | -0.162 | 0.093 | 0.081 |
| rs71390846-1 | -0.100 | 0.038 | 0.008 |  | -0.024 | 0.038 | 0.526 |  | -0.030 | 0.038 | 0.420 |
| rs7135535-2 | -0.067 | 0.050 | 0.179 |  | -0.079 | 0.050 | 0.116 |  | -0.086 | 0.050 | 0.086 |
| rs7135535-1 | -0.102 | 0.038 | 0.008 |  | -0.108 | 0.039 | 0.005 |  | -0.094 | 0.038 | 0.014 |
| rs71330619-2 | 0.448 | 0.226 | 0.047 |  | 0.617 | 0.227 | 0.007 |  | 0.187 | 0.225 | 0.406 |
| rs71330619-1 | 0.487 | 0.228 | 0.033 |  | 0.661 | 0.229 | 0.004 |  | 0.174 | 0.227 | 0.443 |
| rs7121746-2 | -0.045 | 0.053 | 0.399 |  | -0.029 | 0.053 | 0.580 |  | -0.051 | 0.053 | 0.335 |
| rs7121746-1 | 0.028 | 0.050 | 0.578 |  | 0.039 | 0.050 | 0.437 |  | 0.005 | 0.050 | 0.917 |
| rs7121378-2 | -0.278 | 0.147 | 0.058 |  | -0.223 | 0.148 | 0.131 |  | -0.066 | 0.147 | 0.652 |
| rs7121378-1 | 0.022 | 0.042 | 0.599 |  | 0.063 | 0.043 | 0.141 |  | 0.049 | 0.042 | 0.248 |
| rs7117516-2 | -0.061 | 0.114 | 0.589 |  | -0.088 | 0.114 | 0.444 |  | -0.048 | 0.113 | 0.674 |
| rs7117516-1 | 0.002 | 0.041 | 0.964 |  | -0.026 | 0.042 | 0.529 |  | 0.012 | 0.041 | 0.765 |
| rs7117140-2 | -0.092 | 0.093 | 0.326 |  | -0.059 | 0.094 | 0.529 |  | 0.093 | 0.093 | 0.317 |
| rs7117140-1 | -0.012 | 0.040 | 0.763 |  | -0.040 | 0.040 | 0.312 |  | 0.003 | 0.040 | 0.948 |
| rs7102-2 | 0.019 | 0.054 | 0.718 |  | -0.007 | 0.054 | 0.897 |  | 0.064 | 0.053 | 0.232 |
| rs7102-1 | 0.032 | 0.037 | 0.383 |  | 0.026 | 0.037 | 0.487 |  | 0.006 | 0.037 | 0.863 |
| rs7090615-2 | -0.005 | 0.052 | 0.928 |  | -0.026 | 0.052 | 0.620 |  | -0.025 | 0.051 | 0.634 |
| rs7090615-1 | -0.033 | 0.038 | 0.376 |  | -0.021 | 0.038 | 0.569 |  | -0.031 | 0.037 | 0.408 |
| rs7072380-2 | 0.044 | 0.049 | 0.367 |  | 0.009 | 0.050 | 0.861 |  | 0.001 | 0.049 | 0.987 |
| rs7072380-1 | 0.046 | 0.045 | 0.305 |  | 0.016 | 0.045 | 0.721 |  | 0.029 | 0.045 | 0.521 |
| rs7070175-2 | 0.106 | 0.120 | 0.378 |  | 0.093 | 0.121 | 0.443 |  | 0.040 | 0.120 | 0.737 |
| rs7070175-1 | 0.092 | 0.123 | 0.452 |  | 0.041 | 0.124 | 0.743 |  | -0.020 | 0.123 | 0.870 |
| rs704-2 | 0.040 | 0.048 | 0.403 |  | 0.003 | 0.048 | 0.946 |  | 0.010 | 0.048 | 0.838 |
| rs704-1 | 0.000 | 0.041 | 0.993 |  | -0.048 | 0.042 | 0.245 |  | -0.018 | 0.041 | 0.655 |
| rs7040344-2 | -0.062 | 0.056 | 0.265 |  | -0.053 | 0.056 | 0.343 |  | -0.023 | 0.055 | 0.674 |
| rs7040344-1 | -0.059 | 0.036 | 0.105 |  | -0.037 | 0.036 | 0.313 |  | -0.007 | 0.036 | 0.837 |
| rs7022162-2 | -0.072 | 0.067 | 0.283 |  | -0.007 | 0.067 | 0.923 |  | 0.067 | 0.067 | 0.319 |
| rs7022162-1 | -0.016 | 0.036 | 0.657 |  | 0.040 | 0.036 | 0.267 |  | 0.013 | 0.036 | 0.729 |
| rs7017252-2 | 0.006 | 0.054 | 0.912 |  | -0.006 | 0.054 | 0.909 |  | 0.026 | 0.054 | 0.624 |
| rs7017252-1 | 0.033 | 0.051 | 0.527 |  | 0.035 | 0.052 | 0.499 |  | 0.038 | 0.051 | 0.454 |
| rs7014448-2 | -0.147 | 0.060 | 0.014 |  | -0.120 | 0.060 | 0.047 |  | -0.092 | 0.060 | 0.127 |
| rs7014448-1 | -0.180 | 0.060 | 0.003 |  | -0.169 | 0.061 | 0.005 |  | -0.095 | 0.060 | 0.113 |
| rs7000279-2 | 0.115 | 0.068 | 0.091 |  | 0.099 | 0.068 | 0.146 |  | -0.050 | 0.068 | 0.461 |
| rs7000279-1 | 0.128 | 0.069 | 0.063 |  | 0.115 | 0.069 | 0.096 |  | -0.059 | 0.069 | 0.390 |
| rs698891-2 | -0.034 | 0.163 | 0.835 |  | -0.022 | 0.163 | 0.892 |  | 0.207 | 0.162 | 0.203 |
| rs698891-1 | -0.040 | 0.167 | 0.812 |  | -0.039 | 0.167 | 0.814 |  | 0.213 | 0.166 | 0.200 |
| rs6985616-2 | 0.004 | 0.048 | 0.941 |  | -0.037 | 0.049 | 0.447 |  | 0.001 | 0.048 | 0.987 |
| rs6985616-1 | -0.015 | 0.045 | 0.733 |  | -0.038 | 0.045 | 0.394 |  | -0.014 | 0.045 | 0.753 |
| rs6968704-2 | -0.039 | 0.065 | 0.551 |  | -0.055 | 0.066 | 0.401 |  | 0.008 | 0.065 | 0.905 |
| rs6968704-1 | -0.044 | 0.040 | 0.273 |  | -0.045 | 0.040 | 0.255 |  | 0.018 | 0.040 | 0.654 |
| rs6966540-2 | -0.008 | 0.052 | 0.880 |  | -0.012 | 0.052 | 0.825 |  | 0.013 | 0.052 | 0.805 |
| rs6966540-1 | -0.079 | 0.049 | 0.107 |  | -0.093 | 0.049 | 0.061 |  | -0.009 | 0.049 | 0.851 |
| rs6965122-2 | -0.002 | 0.061 | 0.979 |  | 0.008 | 0.062 | 0.899 |  | -0.044 | 0.061 | 0.477 |
| rs6965122-1 | -0.044 | 0.038 | 0.240 |  | -0.032 | 0.038 | 0.406 |  | -0.003 | 0.038 | 0.934 |
| rs6958059-2 | -0.068 | 0.052 | 0.191 |  | -0.057 | 0.052 | 0.276 |  | -0.012 | 0.052 | 0.813 |
| rs6958059-1 | -0.078 | 0.037 | 0.037 |  | -0.074 | 0.038 | 0.048 |  | -0.007 | 0.037 | 0.842 |
| rs6938070-2 | -0.034 | 0.062 | 0.583 |  | -0.015 | 0.062 | 0.811 |  | -0.092 | 0.062 | 0.135 |
| rs6938070-1 | -0.050 | 0.063 | 0.427 |  | -0.045 | 0.063 | 0.478 |  | -0.100 | 0.063 | 0.113 |
| rs6938004-2 | 0.006 | 0.050 | 0.898 |  | -0.045 | 0.051 | 0.370 |  | 0.037 | 0.050 | 0.460 |
| rs6938004-1 | 0.039 | 0.047 | 0.406 |  | -0.033 | 0.047 | 0.485 |  | 0.030 | 0.047 | 0.520 |
| rs6932260-2 | -0.093 | 0.054 | 0.087 |  | -0.099 | 0.055 | 0.070 |  | -0.044 | 0.054 | 0.423 |
| rs6932260-1 | -0.100 | 0.045 | 0.025 |  | -0.095 | 0.045 | 0.036 |  | -0.030 | 0.045 | 0.497 |
| rs6931664-2 | -0.051 | 0.065 | 0.439 |  | -0.084 | 0.066 | 0.199 |  | -0.051 | 0.065 | 0.431 |
| rs6931664-1 | -0.069 | 0.044 | 0.114 |  | -0.059 | 0.044 | 0.184 |  | -0.043 | 0.044 | 0.328 |
| rs6930181-2 | 0.026 | 0.049 | 0.595 |  | -0.031 | 0.049 | 0.524 |  | 0.002 | 0.049 | 0.974 |
| rs6930181-1 | 0.008 | 0.040 | 0.844 |  | -0.026 | 0.041 | 0.514 |  | -0.008 | 0.040 | 0.835 |
| rs6912283-2 | -0.033 | 0.049 | 0.507 |  | -0.043 | 0.049 | 0.387 |  | -0.027 | 0.049 | 0.582 |
| rs6912283-1 | 0.007 | 0.045 | 0.880 |  | -0.017 | 0.046 | 0.712 |  | 0.018 | 0.045 | 0.685 |
| rs6905582-2 | -0.037 | 0.105 | 0.728 |  | -0.008 | 0.106 | 0.938 |  | -0.179 | 0.105 | 0.090 |
| rs6905582-1 | -0.016 | 0.042 | 0.704 |  | -0.020 | 0.042 | 0.628 |  | -0.025 | 0.042 | 0.555 |
| rs6903443-2 | 0.015 | 0.052 | 0.771 |  | -0.021 | 0.052 | 0.690 |  | 0.082 | 0.052 | 0.115 |
| rs6903443-1 | -0.010 | 0.046 | 0.836 |  | -0.007 | 0.046 | 0.884 |  | 0.002 | 0.046 | 0.962 |
| rs6901631-2 | 0.000 | 0.115 | 1.000 |  | 0.004 | 0.115 | 0.970 |  | 0.117 | 0.115 | 0.307 |
| rs6901631-1 | -0.010 | 0.118 | 0.933 |  | -0.004 | 0.119 | 0.975 |  | 0.074 | 0.118 | 0.528 |
| rs6885822-2 | 0.106 | 0.115 | 0.355 |  | 0.122 | 0.115 | 0.290 |  | 0.076 | 0.115 | 0.506 |
| rs6885822-1 | 0.017 | 0.040 | 0.672 |  | 0.000 | 0.041 | 0.994 |  | -0.035 | 0.040 | 0.378 |
| rs6882422-2 | 0.085 | 0.154 | 0.579 |  | 0.066 | 0.155 | 0.670 |  | -0.079 | 0.154 | 0.607 |
| rs6882422-1 | 0.009 | 0.045 | 0.837 |  | -0.001 | 0.046 | 0.974 |  | 0.004 | 0.045 | 0.931 |
| rs6879220-2 | 0.062 | 0.052 | 0.233 |  | 0.047 | 0.053 | 0.370 |  | 0.021 | 0.052 | 0.681 |
| rs6879220-1 | 0.047 | 0.051 | 0.351 |  | 0.048 | 0.051 | 0.342 |  | 0.024 | 0.050 | 0.635 |
| rs687914-2 | -0.046 | 0.078 | 0.549 |  | -0.034 | 0.078 | 0.666 |  | 0.019 | 0.077 | 0.809 |
| rs687914-1 | -0.072 | 0.036 | 0.049 |  | -0.081 | 0.037 | 0.026 |  | -0.104 | 0.036 | 0.004 |
| rs6875585-2 | -0.015 | 0.056 | 0.787 |  | -0.008 | 0.056 | 0.884 |  | -0.077 | 0.055 | 0.163 |
| rs6875585-1 | -0.004 | 0.037 | 0.915 |  | 0.001 | 0.037 | 0.968 |  | -0.014 | 0.037 | 0.708 |
| rs6870880-2 | 0.011 | 0.053 | 0.834 |  | 0.018 | 0.053 | 0.736 |  | 0.025 | 0.052 | 0.640 |
| rs6870880-1 | -0.002 | 0.037 | 0.954 |  | 0.025 | 0.037 | 0.502 |  | 0.039 | 0.037 | 0.290 |
| rs6870556-2 | 0.032 | 0.051 | 0.529 |  | 0.027 | 0.052 | 0.595 |  | 0.076 | 0.051 | 0.137 |
| rs6870556-1 | 0.048 | 0.050 | 0.340 |  | 0.057 | 0.050 | 0.254 |  | 0.066 | 0.050 | 0.182 |
| rs6864688-2 | 0.008 | 0.050 | 0.879 |  | -0.017 | 0.050 | 0.737 |  | 0.020 | 0.050 | 0.689 |
| rs6864688-1 | 0.021 | 0.044 | 0.634 |  | 0.040 | 0.044 | 0.370 |  | 0.077 | 0.044 | 0.079 |
| rs6861681-2 | -0.006 | 0.062 | 0.929 |  | -0.001 | 0.062 | 0.982 |  | -0.038 | 0.062 | 0.539 |
| rs6861681-1 | 0.038 | 0.062 | 0.538 |  | 0.047 | 0.063 | 0.453 |  | -0.004 | 0.062 | 0.945 |
| rs6839437-2 | 0.124 | 0.092 | 0.176 |  | 0.104 | 0.092 | 0.261 |  | -0.078 | 0.092 | 0.396 |
| rs6839437-1 | 0.071 | 0.038 | 0.059 |  | 0.051 | 0.038 | 0.181 |  | 0.006 | 0.038 | 0.869 |
| rs680386-2 | -0.059 | 0.086 | 0.494 |  | -0.088 | 0.086 | 0.304 |  | -0.087 | 0.085 | 0.308 |
| rs680386-1 | 0.004 | 0.088 | 0.964 |  | -0.028 | 0.089 | 0.749 |  | 0.003 | 0.088 | 0.977 |
| rs6803137-2 | -0.044 | 0.083 | 0.596 |  | 0.013 | 0.083 | 0.878 |  | 0.056 | 0.082 | 0.495 |
| rs6803137-1 | -0.008 | 0.037 | 0.820 |  | 0.006 | 0.037 | 0.865 |  | 0.039 | 0.037 | 0.298 |
| rs6794670-2 | 0.153 | 0.079 | 0.054 |  | 0.165 | 0.080 | 0.038 |  | 0.084 | 0.079 | 0.290 |
| rs6794670-1 | -0.010 | 0.036 | 0.790 |  | -0.013 | 0.036 | 0.715 |  | -0.073 | 0.036 | 0.042 |
| rs6784925-2 | -0.040 | 0.049 | 0.415 |  | -0.022 | 0.049 | 0.654 |  | 0.041 | 0.049 | 0.400 |
| rs6784925-1 | 0.011 | 0.045 | 0.811 |  | -0.016 | 0.045 | 0.727 |  | 0.073 | 0.045 | 0.104 |
| rs6782178-2 | 0.059 | 0.050 | 0.238 |  | 0.063 | 0.050 | 0.207 |  | 0.025 | 0.050 | 0.610 |
| rs6782178-1 | 0.040 | 0.047 | 0.399 |  | 0.009 | 0.047 | 0.851 |  | 0.015 | 0.047 | 0.751 |
| rs67820526-2 | 0.022 | 0.094 | 0.812 |  | 0.050 | 0.095 | 0.595 |  | -0.085 | 0.094 | 0.369 |
| rs67820526-1 | -0.004 | 0.097 | 0.963 |  | 0.020 | 0.097 | 0.837 |  | -0.085 | 0.097 | 0.379 |
| rs67820074-2 | 0.044 | 0.266 | 0.868 |  | 0.188 | 0.267 | 0.481 |  | 0.095 | 0.265 | 0.720 |
| rs67820074-1 | 0.035 | 0.268 | 0.895 |  | 0.138 | 0.270 | 0.609 |  | 0.083 | 0.268 | 0.755 |
| rs67758468-2 | -0.013 | 0.074 | 0.862 |  | -0.034 | 0.074 | 0.650 |  | -0.115 | 0.073 | 0.117 |
| rs67758468-1 | -0.025 | 0.075 | 0.740 |  | -0.080 | 0.076 | 0.291 |  | -0.115 | 0.075 | 0.126 |
| rs6769511-2 | -0.058 | 0.058 | 0.318 |  | -0.013 | 0.058 | 0.820 |  | -0.126 | 0.058 | 0.029 |
| rs6769511-1 | -0.072 | 0.058 | 0.216 |  | -0.017 | 0.058 | 0.766 |  | -0.083 | 0.058 | 0.149 |
| rs6761320-2 | -0.056 | 0.049 | 0.260 |  | -0.024 | 0.050 | 0.622 |  | -0.038 | 0.049 | 0.440 |
| rs6761320-1 | -0.007 | 0.046 | 0.884 |  | 0.007 | 0.046 | 0.874 |  | 0.025 | 0.045 | 0.579 |
| rs67575951-2 | -0.536 | 0.250 | 0.032 |  | -0.662 | 0.252 | 0.009 |  | -0.275 | 0.250 | 0.271 |
| rs67575951-1 | -0.044 | 0.052 | 0.396 |  | -0.048 | 0.052 | 0.357 |  | -0.021 | 0.052 | 0.682 |
| rs672740-2 | 0.015 | 0.052 | 0.772 |  | 0.044 | 0.053 | 0.408 |  | 0.009 | 0.052 | 0.869 |
| rs672740-1 | -0.053 | 0.043 | 0.223 |  | -0.035 | 0.043 | 0.421 |  | -0.024 | 0.043 | 0.583 |
| rs6722557-2 | 0.044 | 0.076 | 0.562 |  | 0.023 | 0.076 | 0.767 |  | -0.124 | 0.076 | 0.100 |
| rs6722557-1 | 0.060 | 0.076 | 0.425 |  | 0.066 | 0.076 | 0.388 |  | -0.089 | 0.076 | 0.237 |
| rs6719806-2 | 0.062 | 0.052 | 0.232 |  | 0.027 | 0.052 | 0.609 |  | -0.037 | 0.052 | 0.475 |
| rs6719806-1 | 0.018 | 0.041 | 0.661 |  | 0.008 | 0.041 | 0.854 |  | 0.023 | 0.041 | 0.563 |
| rs6716216-2 | -0.108 | 0.146 | 0.460 |  | -0.075 | 0.147 | 0.610 |  | -0.176 | 0.146 | 0.227 |
| rs6716216-1 | -0.066 | 0.148 | 0.657 |  | -0.060 | 0.149 | 0.688 |  | -0.143 | 0.148 | 0.334 |
| rs6711976-2 | 0.100 | 0.060 | 0.095 |  | 0.079 | 0.060 | 0.187 |  | 0.004 | 0.060 | 0.942 |
| rs6711976-1 | 0.106 | 0.036 | 0.003 |  | 0.105 | 0.036 | 0.004 |  | 0.004 | 0.036 | 0.912 |
| rs6701977-2 | -0.099 | 0.111 | 0.371 |  | -0.071 | 0.111 | 0.524 |  | -0.036 | 0.110 | 0.745 |
| rs6701977-1 | -0.058 | 0.041 | 0.152 |  | -0.041 | 0.041 | 0.316 |  | 0.021 | 0.041 | 0.600 |
| rs66906321-2 | 0.023 | 0.097 | 0.812 |  | 0.029 | 0.097 | 0.767 |  | -0.053 | 0.097 | 0.581 |
| rs66906321-1 | 0.004 | 0.038 | 0.925 |  | 0.020 | 0.038 | 0.593 |  | 0.012 | 0.038 | 0.741 |
| rs6680369-2 | -0.073 | 0.051 | 0.154 |  | -0.070 | 0.051 | 0.172 |  | -0.070 | 0.051 | 0.167 |
| rs6680369-1 | 0.014 | 0.049 | 0.782 |  | 0.027 | 0.049 | 0.587 |  | 0.033 | 0.049 | 0.504 |
| rs6672925-2 | -0.044 | 0.063 | 0.487 |  | -0.008 | 0.064 | 0.904 |  | 0.069 | 0.063 | 0.273 |
| rs6672925-1 | -0.027 | 0.064 | 0.673 |  | -0.027 | 0.064 | 0.675 |  | 0.038 | 0.064 | 0.555 |
| rs6664489-2 | 0.013 | 0.077 | 0.864 |  | 0.078 | 0.077 | 0.312 |  | 0.056 | 0.076 | 0.465 |
| rs6664489-1 | 0.009 | 0.037 | 0.813 |  | 0.046 | 0.037 | 0.213 |  | -0.017 | 0.037 | 0.635 |
| rs66620549-2 | -0.058 | 0.076 | 0.443 |  | -0.049 | 0.076 | 0.524 |  | -0.169 | 0.076 | 0.026 |
| rs66620549-1 | 0.011 | 0.037 | 0.762 |  | -0.019 | 0.037 | 0.598 |  | -0.002 | 0.037 | 0.964 |
| rs66470941-2 | 0.024 | 0.110 | 0.826 |  | -0.006 | 0.110 | 0.959 |  | -0.135 | 0.109 | 0.218 |
| rs66470941-1 | 0.014 | 0.042 | 0.736 |  | 0.027 | 0.042 | 0.523 |  | 0.005 | 0.042 | 0.904 |
| rs660240-2 | 0.045 | 0.078 | 0.570 |  | 0.096 | 0.079 | 0.222 |  | 0.026 | 0.078 | 0.738 |
| rs660240-1 | 0.013 | 0.080 | 0.871 |  | 0.045 | 0.080 | 0.579 |  | 0.026 | 0.080 | 0.742 |
| rs6583866-2 | -0.079 | 0.049 | 0.108 |  | -0.078 | 0.049 | 0.116 |  | -0.056 | 0.049 | 0.254 |
| rs6583866-1 | -0.031 | 0.046 | 0.502 |  | -0.044 | 0.046 | 0.344 |  | -0.015 | 0.046 | 0.743 |
| rs6566169-2 | -0.033 | 0.048 | 0.483 |  | -0.067 | 0.048 | 0.164 |  | -0.054 | 0.048 | 0.255 |
| rs6566169-1 | -0.048 | 0.041 | 0.243 |  | -0.070 | 0.041 | 0.092 |  | -0.066 | 0.041 | 0.106 |
| rs6564890-2 | 0.021 | 0.049 | 0.666 |  | 0.042 | 0.049 | 0.391 |  | 0.023 | 0.049 | 0.631 |
| rs6564890-1 | 0.001 | 0.042 | 0.990 |  | 0.024 | 0.042 | 0.574 |  | -0.026 | 0.042 | 0.530 |
| rs6546334-2 | 0.088 | 0.055 | 0.109 |  | 0.120 | 0.055 | 0.030 |  | 0.203 | 0.055 | 0.000 |
| rs6546334-1 | -0.058 | 0.037 | 0.114 |  | -0.058 | 0.037 | 0.120 |  | 0.007 | 0.037 | 0.843 |
| rs6544633-2 | -0.106 | 0.061 | 0.085 |  | -0.088 | 0.062 | 0.153 |  | -0.083 | 0.061 | 0.174 |
| rs6544633-1 | -0.132 | 0.061 | 0.032 |  | -0.124 | 0.062 | 0.044 |  | -0.117 | 0.061 | 0.056 |
| rs6542920-2 | -0.075 | 0.054 | 0.170 |  | -0.012 | 0.055 | 0.830 |  | -0.082 | 0.054 | 0.129 |
| rs6542920-1 | -0.026 | 0.036 | 0.469 |  | -0.005 | 0.037 | 0.895 |  | -0.014 | 0.036 | 0.696 |
| rs6485702-2 | -0.147 | 0.055 | 0.008 |  | -0.151 | 0.056 | 0.007 |  | -0.091 | 0.055 | 0.098 |
| rs6485702-1 | -0.149 | 0.055 | 0.007 |  | -0.161 | 0.055 | 0.004 |  | -0.095 | 0.055 | 0.083 |
| rs6476491-2 | 0.059 | 0.077 | 0.442 |  | 0.006 | 0.078 | 0.937 |  | -0.130 | 0.077 | 0.091 |
| rs6476491-1 | 0.065 | 0.037 | 0.081 |  | 0.056 | 0.037 | 0.132 |  | -0.002 | 0.037 | 0.950 |
| rs6471752-2 | -0.003 | 0.097 | 0.976 |  | -0.036 | 0.098 | 0.716 |  | 0.106 | 0.097 | 0.276 |
| rs6471752-1 | -0.022 | 0.039 | 0.571 |  | 0.000 | 0.039 | 0.992 |  | 0.024 | 0.039 | 0.542 |
| rs6467366-2 | 0.200 | 0.091 | 0.029 |  | 0.134 | 0.092 | 0.144 |  | 0.060 | 0.091 | 0.511 |
| rs6467366-1 | 0.200 | 0.093 | 0.032 |  | 0.127 | 0.093 | 0.174 |  | 0.017 | 0.093 | 0.851 |
| rs6449774-2 | 0.017 | 0.050 | 0.737 |  | 0.036 | 0.050 | 0.472 |  | 0.094 | 0.050 | 0.058 |
| rs6449774-1 | -0.016 | 0.039 | 0.688 |  | -0.027 | 0.039 | 0.484 |  | 0.047 | 0.039 | 0.224 |
| rs6421572-2 | 0.062 | 0.134 | 0.643 |  | 0.098 | 0.134 | 0.465 |  | 0.110 | 0.133 | 0.410 |
| rs6421572-1 | 0.010 | 0.041 | 0.817 |  | 0.013 | 0.041 | 0.755 |  | 0.057 | 0.041 | 0.165 |
| rs6415968-2 | -0.063 | 0.048 | 0.189 |  | -0.073 | 0.048 | 0.127 |  | 0.009 | 0.048 | 0.854 |
| rs6415968-1 | -0.085 | 0.042 | 0.045 |  | -0.057 | 0.042 | 0.178 |  | -0.033 | 0.042 | 0.426 |
| rs635634-2 | -0.083 | 0.090 | 0.359 |  | -0.106 | 0.091 | 0.245 |  | -0.195 | 0.090 | 0.030 |
| rs635634-1 | 0.040 | 0.037 | 0.277 |  | 0.025 | 0.037 | 0.504 |  | 0.016 | 0.037 | 0.656 |
| rs630539-2 | -0.317 | 0.284 | 0.264 |  | -0.175 | 0.285 | 0.538 |  | -0.233 | 0.283 | 0.410 |
| rs630539-1 | 0.124 | 0.061 | 0.040 |  | 0.079 | 0.061 | 0.191 |  | -0.057 | 0.060 | 0.347 |
| rs62625030-2 | 0.047 | 0.151 | 0.753 |  | -0.010 | 0.151 | 0.948 |  | 0.116 | 0.150 | 0.442 |
| rs62621812-2 | 0.101 | 0.696 | 0.885 |  | -0.348 | 0.699 | 0.619 |  | -0.174 | 0.694 | 0.802 |
| rs62621812-1 | 0.199 | 0.091 | 0.028 |  | 0.094 | 0.091 | 0.305 |  | 0.015 | 0.091 | 0.864 |
| rs6258-2 | -0.184 | 0.149 | 0.216 |  | -0.245 | 0.150 | 0.102 |  | -0.250 | 0.149 | 0.093 |
| rs62561562-2 | -0.041 | 0.054 | 0.450 |  | -0.049 | 0.055 | 0.371 |  | -0.064 | 0.054 | 0.239 |
| rs62561562-1 | -0.003 | 0.054 | 0.955 |  | -0.002 | 0.054 | 0.973 |  | 0.003 | 0.054 | 0.961 |
| rs62459989-2 | 0.068 | 0.095 | 0.475 |  | -0.008 | 0.095 | 0.934 |  | 0.041 | 0.095 | 0.667 |
| rs62459989-1 | 0.059 | 0.098 | 0.548 |  | -0.011 | 0.098 | 0.911 |  | 0.013 | 0.098 | 0.896 |
| rs62453057-2 | -0.152 | 0.110 | 0.169 |  | -0.090 | 0.111 | 0.415 |  | -0.015 | 0.110 | 0.889 |
| rs62453057-1 | -0.159 | 0.113 | 0.160 |  | -0.110 | 0.114 | 0.335 |  | -0.043 | 0.113 | 0.702 |
| rs62427108-2 | 0.260 | 0.307 | 0.397 |  | 0.415 | 0.308 | 0.179 |  | -0.120 | 0.306 | 0.694 |
| rs62427108-1 | -0.043 | 0.058 | 0.455 |  | -0.034 | 0.058 | 0.564 |  | 0.065 | 0.058 | 0.260 |
| rs62404993-2 | 0.436 | 0.379 | 0.251 |  | 0.572 | 0.381 | 0.133 |  | 0.499 | 0.378 | 0.187 |
| rs62404993-1 | -0.013 | 0.068 | 0.844 |  | -0.012 | 0.068 | 0.864 |  | 0.075 | 0.068 | 0.272 |
| rs62321667-2 | 0.221 | 0.139 | 0.111 |  | 0.107 | 0.139 | 0.444 |  | 0.290 | 0.138 | 0.036 |
| rs62321667-1 | -0.004 | 0.042 | 0.931 |  | -0.043 | 0.042 | 0.308 |  | -0.007 | 0.042 | 0.876 |
| rs62302300-2 | 0.022 | 0.109 | 0.842 |  | -0.005 | 0.110 | 0.964 |  | 0.018 | 0.109 | 0.866 |
| rs62302300-1 | -0.014 | 0.040 | 0.720 |  | -0.046 | 0.040 | 0.251 |  | -0.012 | 0.040 | 0.764 |
| rs62294148-2 | 0.019 | 0.250 | 0.939 |  | -0.403 | 0.251 | 0.108 |  | -0.045 | 0.249 | 0.855 |
| rs62294148-1 | 0.029 | 0.254 | 0.910 |  | -0.334 | 0.255 | 0.190 |  | -0.054 | 0.253 | 0.830 |
| rs62271373-2 | 0.185 | 0.319 | 0.561 |  | 0.094 | 0.320 | 0.769 |  | 0.055 | 0.318 | 0.863 |
| rs62271373-1 | 0.275 | 0.323 | 0.395 |  | 0.170 | 0.324 | 0.600 |  | 0.188 | 0.321 | 0.558 |
| rs62261502-2 | 0.025 | 0.231 | 0.915 |  | 0.087 | 0.232 | 0.707 |  | 0.278 | 0.230 | 0.227 |
| rs62261502-1 | -0.007 | 0.235 | 0.976 |  | 0.081 | 0.236 | 0.732 |  | 0.256 | 0.234 | 0.275 |
| rs62228064-2 | -0.132 | 0.115 | 0.251 |  | -0.116 | 0.115 | 0.314 |  | 0.213 | 0.115 | 0.064 |
| rs62228064-1 | -0.100 | 0.117 | 0.392 |  | -0.072 | 0.118 | 0.539 |  | 0.263 | 0.117 | 0.025 |
| rs62182131-2 | 0.125 | 0.294 | 0.672 |  | 0.040 | 0.295 | 0.893 |  | 0.131 | 0.293 | 0.656 |
| rs62182131-1 | 0.036 | 0.051 | 0.485 |  | 0.030 | 0.051 | 0.556 |  | 0.008 | 0.051 | 0.871 |
| rs62179714-2 | 0.016 | 0.066 | 0.811 |  | 0.010 | 0.066 | 0.881 |  | -0.007 | 0.066 | 0.910 |
| rs62179714-1 | 0.020 | 0.036 | 0.566 |  | 0.028 | 0.036 | 0.433 |  | 0.034 | 0.036 | 0.339 |
| rs62165280-2 | -0.106 | 0.085 | 0.215 |  | -0.126 | 0.086 | 0.142 |  | -0.140 | 0.085 | 0.100 |
| rs62165280-1 | -0.106 | 0.087 | 0.220 |  | -0.091 | 0.087 | 0.295 |  | -0.105 | 0.087 | 0.225 |
| rs62063930-2 | 0.048 | 0.124 | 0.696 |  | 0.083 | 0.125 | 0.508 |  | -0.033 | 0.124 | 0.789 |
| rs62063930-1 | 0.023 | 0.040 | 0.571 |  | 0.031 | 0.040 | 0.433 |  | 0.005 | 0.040 | 0.910 |
| rs62007684-2 | -0.080 | 0.054 | 0.139 |  | -0.078 | 0.054 | 0.153 |  | -0.094 | 0.054 | 0.083 |
| rs62007684-1 | -0.048 | 0.037 | 0.197 |  | -0.058 | 0.038 | 0.125 |  | -0.035 | 0.037 | 0.345 |
| rs61998565-2 | 0.056 | 0.131 | 0.671 |  | 0.000 | 0.132 | 0.999 |  | 0.143 | 0.131 | 0.274 |
| rs61998565-1 | -0.015 | 0.040 | 0.708 |  | -0.004 | 0.041 | 0.915 |  | 0.006 | 0.040 | 0.887 |
| rs61903841-2 | -0.061 | 0.052 | 0.237 |  | -0.044 | 0.052 | 0.403 |  | -0.064 | 0.052 | 0.216 |
| rs61903841-1 | 0.048 | 0.038 | 0.212 |  | 0.050 | 0.038 | 0.190 |  | -0.013 | 0.038 | 0.735 |
| rs61887821-1 | 0.021 | 0.176 | 0.905 |  | -0.043 | 0.177 | 0.809 |  | 0.056 | 0.175 | 0.749 |
| rs6185-2 | -0.073 | 0.070 | 0.301 |  | -0.123 | 0.070 | 0.082 |  | -0.204 | 0.070 | 0.003 |
| rs6185-1 | -0.078 | 0.036 | 0.032 |  | -0.082 | 0.037 | 0.026 |  | -0.090 | 0.036 | 0.013 |
| rs61780429-2 | -0.153 | 0.089 | 0.084 |  | -0.065 | 0.089 | 0.464 |  | -0.019 | 0.088 | 0.826 |
| rs61780429-1 | 0.044 | 0.038 | 0.246 |  | 0.051 | 0.038 | 0.175 |  | -0.003 | 0.038 | 0.939 |
| rs61758378-1 | -0.002 | 0.212 | 0.992 |  | -0.119 | 0.213 | 0.576 |  | 0.087 | 0.212 | 0.680 |
| rs61748861-2 | -0.070 | 0.232 | 0.762 |  | 0.143 | 0.234 | 0.540 |  | -0.089 | 0.232 | 0.701 |
| rs61735998-2 | 0.440 | 0.702 | 0.531 |  | 0.168 | 0.705 | 0.812 |  | -1.023 | 0.700 | 0.144 |
| rs61735998-1 | 0.271 | 0.709 | 0.702 |  | 0.089 | 0.712 | 0.901 |  | -0.918 | 0.707 | 0.194 |
| rs61733768-2 | -0.581 | 0.691 | 0.401 |  | -0.050 | 0.694 | 0.943 |  | -0.121 | 0.689 | 0.860 |
| rs61733768-1 | -0.071 | 0.086 | 0.409 |  | -0.077 | 0.086 | 0.373 |  | -0.017 | 0.086 | 0.838 |
| rs61578783-2 | 0.018 | 0.103 | 0.858 |  | -0.032 | 0.104 | 0.759 |  | 0.107 | 0.103 | 0.296 |
| rs61578783-1 | 0.065 | 0.039 | 0.092 |  | 0.024 | 0.039 | 0.537 |  | 0.058 | 0.039 | 0.131 |
| rs614802-2 | 0.009 | 0.061 | 0.878 |  | 0.075 | 0.062 | 0.225 |  | 0.055 | 0.061 | 0.367 |
| rs614802-1 | 0.037 | 0.062 | 0.546 |  | 0.075 | 0.062 | 0.229 |  | 0.067 | 0.062 | 0.277 |
| rs6142137-2 | 0.024 | 0.056 | 0.668 |  | 0.048 | 0.056 | 0.390 |  | 0.062 | 0.056 | 0.266 |
| rs6142137-1 | 0.033 | 0.051 | 0.519 |  | 0.047 | 0.051 | 0.354 |  | 0.049 | 0.051 | 0.339 |
| rs6134038-2 | -0.225 | 0.090 | 0.012 |  | -0.204 | 0.090 | 0.023 |  | -0.205 | 0.089 | 0.022 |
| rs6134038-1 | -0.025 | 0.039 | 0.518 |  | -0.028 | 0.039 | 0.482 |  | -0.005 | 0.039 | 0.892 |
| rs613223-2 | 0.019 | 0.056 | 0.727 |  | -0.030 | 0.056 | 0.591 |  | -0.027 | 0.055 | 0.626 |
| rs613223-1 | 0.061 | 0.037 | 0.094 |  | 0.022 | 0.037 | 0.543 |  | 0.004 | 0.036 | 0.907 |
| rs6129493-2 | -0.112 | 0.070 | 0.108 |  | -0.103 | 0.070 | 0.143 |  | -0.056 | 0.070 | 0.423 |
| rs6129493-1 | -0.008 | 0.036 | 0.836 |  | 0.011 | 0.036 | 0.771 |  | -0.001 | 0.036 | 0.973 |
| rs6120804-2 | -0.153 | 0.096 | 0.114 |  | -0.135 | 0.097 | 0.163 |  | -0.145 | 0.096 | 0.131 |
| rs6120804-1 | -0.084 | 0.096 | 0.383 |  | -0.077 | 0.097 | 0.428 |  | -0.051 | 0.096 | 0.593 |
| rs6117854-2 | 0.051 | 0.063 | 0.425 |  | -0.009 | 0.064 | 0.886 |  | -0.042 | 0.063 | 0.504 |
| rs6117854-1 | 0.013 | 0.037 | 0.717 |  | -0.029 | 0.037 | 0.428 |  | 0.000 | 0.037 | 0.993 |
| rs6117637-2 | -0.070 | 0.077 | 0.358 |  | -0.078 | 0.077 | 0.313 |  | -0.022 | 0.076 | 0.772 |
| rs6117637-1 | -0.043 | 0.037 | 0.239 |  | -0.038 | 0.037 | 0.306 |  | -0.066 | 0.037 | 0.073 |
| rs6117294-2 | -0.153 | 0.050 | 0.002 |  | -0.131 | 0.050 | 0.010 |  | -0.089 | 0.050 | 0.074 |
| rs6117294-1 | -0.126 | 0.038 | 0.001 |  | -0.135 | 0.038 | 0.000 |  | -0.099 | 0.038 | 0.008 |
| rs61171454-2 | -0.832 | 0.390 | 0.033 |  | -0.576 | 0.392 | 0.142 |  | -0.414 | 0.389 | 0.287 |
| rs61171454-1 | -0.098 | 0.078 | 0.207 |  | -0.111 | 0.078 | 0.154 |  | -0.014 | 0.078 | 0.857 |
| rs6108796-2 | 0.034 | 0.071 | 0.629 |  | 0.006 | 0.071 | 0.936 |  | 0.009 | 0.071 | 0.897 |
| rs6108796-1 | -0.016 | 0.069 | 0.814 |  | -0.018 | 0.069 | 0.798 |  | -0.019 | 0.069 | 0.780 |
| rs60891864-2 | 0.062 | 0.052 | 0.232 |  | 0.007 | 0.052 | 0.894 |  | 0.046 | 0.051 | 0.371 |
| rs60891864-1 | 0.057 | 0.049 | 0.247 |  | 0.054 | 0.049 | 0.274 |  | 0.034 | 0.049 | 0.488 |
| rs6088855-2 | -0.033 | 0.082 | 0.692 |  | -0.045 | 0.083 | 0.586 |  | -0.103 | 0.082 | 0.211 |
| rs6088855-1 | -0.063 | 0.085 | 0.457 |  | -0.078 | 0.085 | 0.359 |  | -0.121 | 0.084 | 0.153 |
| rs6086143-2 | -0.075 | 0.052 | 0.154 |  | -0.046 | 0.053 | 0.378 |  | 0.005 | 0.052 | 0.930 |
| rs6086143-1 | -0.044 | 0.040 | 0.275 |  | -0.030 | 0.040 | 0.449 |  | -0.009 | 0.040 | 0.825 |
| rs6077999-2 | 0.065 | 0.069 | 0.347 |  | 0.069 | 0.070 | 0.326 |  | -0.088 | 0.069 | 0.205 |
| rs6077999-1 | -0.033 | 0.066 | 0.622 |  | -0.043 | 0.067 | 0.520 |  | -0.168 | 0.066 | 0.011 |
| rs6063051-2 | -0.019 | 0.066 | 0.771 |  | -0.065 | 0.066 | 0.327 |  | 0.002 | 0.066 | 0.977 |
| rs6063051-1 | -0.028 | 0.036 | 0.440 |  | -0.091 | 0.036 | 0.011 |  | -0.042 | 0.036 | 0.239 |
| rs6059412-2 | 0.012 | 0.061 | 0.850 |  | -0.019 | 0.062 | 0.755 |  | -0.040 | 0.061 | 0.511 |
| rs6059412-1 | 0.006 | 0.061 | 0.926 |  | -0.003 | 0.061 | 0.959 |  | -0.054 | 0.061 | 0.372 |
| rs60574493-2 | 0.048 | 0.111 | 0.663 |  | 0.007 | 0.111 | 0.951 |  | -0.069 | 0.110 | 0.529 |
| rs60574493-1 | 0.061 | 0.113 | 0.592 |  | 0.028 | 0.114 | 0.803 |  | -0.065 | 0.113 | 0.565 |
| rs605228-2 | -0.058 | 0.060 | 0.331 |  | -0.074 | 0.060 | 0.214 |  | 0.029 | 0.059 | 0.621 |
| rs605228-1 | -0.067 | 0.060 | 0.265 |  | -0.089 | 0.061 | 0.145 |  | -0.029 | 0.060 | 0.628 |
| rs603424-2 | -0.146 | 0.084 | 0.082 |  | -0.079 | 0.084 | 0.348 |  | -0.032 | 0.084 | 0.698 |
| rs603424-1 | -0.019 | 0.038 | 0.612 |  | -0.009 | 0.038 | 0.820 |  | 0.010 | 0.038 | 0.786 |
| rs6032946-2 | 0.057 | 0.076 | 0.456 |  | 0.020 | 0.076 | 0.790 |  | 0.037 | 0.076 | 0.624 |
| rs6032946-1 | -0.050 | 0.037 | 0.178 |  | -0.050 | 0.037 | 0.177 |  | -0.006 | 0.037 | 0.863 |
| rs6006426-2 | 0.011 | 0.048 | 0.822 |  | 0.016 | 0.048 | 0.735 |  | -0.023 | 0.048 | 0.638 |
| rs6006426-1 | 0.061 | 0.044 | 0.165 |  | 0.047 | 0.044 | 0.288 |  | 0.060 | 0.044 | 0.170 |
| rs59900779-2 | -0.050 | 0.109 | 0.644 |  | -0.004 | 0.110 | 0.972 |  | -0.086 | 0.109 | 0.432 |
| rs59900779-1 | -0.026 | 0.038 | 0.500 |  | 0.010 | 0.039 | 0.793 |  | -0.026 | 0.038 | 0.494 |
| rs59813731-2 | -0.049 | 0.081 | 0.545 |  | -0.008 | 0.082 | 0.919 |  | -0.009 | 0.081 | 0.912 |
| rs59813731-1 | -0.053 | 0.084 | 0.532 |  | 0.007 | 0.085 | 0.933 |  | -0.015 | 0.084 | 0.857 |
| rs5952416-2 | -0.207 | 1.185 | 0.861 |  | 0.843 | 1.191 | 0.479 |  | -0.825 | 1.181 | 0.485 |
| rs5952416-1 | -0.040 | 0.044 | 0.365 |  | -0.036 | 0.045 | 0.413 |  | -0.007 | 0.044 | 0.870 |
| rs59455052-2 | -0.067 | 0.062 | 0.279 |  | -0.035 | 0.062 | 0.573 |  | 0.035 | 0.062 | 0.571 |
| rs59455052-1 | 0.007 | 0.036 | 0.839 |  | -0.010 | 0.036 | 0.778 |  | -0.021 | 0.036 | 0.561 |
| rs5933080-1 | 0.014 | 0.040 | 0.735 |  | -0.008 | 0.040 | 0.841 |  | -0.019 | 0.040 | 0.627 |
| rs5914035-1 | -0.096 | 0.038 | 0.011 |  | -0.103 | 0.038 | 0.007 |  | -0.094 | 0.038 | 0.013 |
| rs5906426-1 | 0.003 | 0.041 | 0.948 |  | 0.026 | 0.041 | 0.522 |  | -0.007 | 0.041 | 0.857 |
| rs59035991-2 | 0.224 | 0.260 | 0.390 |  | 0.166 | 0.261 | 0.526 |  | -0.107 | 0.259 | 0.679 |
| rs59035991-1 | 0.172 | 0.262 | 0.513 |  | 0.082 | 0.263 | 0.757 |  | -0.136 | 0.261 | 0.602 |
| rs58621819-2 | -0.108 | 0.087 | 0.214 |  | -0.102 | 0.087 | 0.240 |  | 0.006 | 0.086 | 0.944 |
| rs58621819-1 | -0.066 | 0.088 | 0.452 |  | -0.078 | 0.088 | 0.373 |  | 0.049 | 0.087 | 0.574 |
| rs58397787-2 | 0.066 | 0.078 | 0.403 |  | 0.019 | 0.079 | 0.807 |  | -0.087 | 0.078 | 0.268 |
| rs58397787-1 | 0.008 | 0.037 | 0.823 |  | 0.018 | 0.037 | 0.634 |  | -0.043 | 0.037 | 0.236 |
| rs58383597-2 | 0.012 | 0.063 | 0.852 |  | -0.023 | 0.063 | 0.715 |  | 0.000 | 0.063 | 0.995 |
| rs58383597-1 | 0.049 | 0.064 | 0.445 |  | -0.062 | 0.064 | 0.329 |  | -0.018 | 0.064 | 0.779 |
| rs57960590-2 | -0.033 | 0.163 | 0.841 |  | -0.122 | 0.163 | 0.454 |  | -0.149 | 0.162 | 0.358 |
| rs57960590-1 | -0.047 | 0.050 | 0.345 |  | -0.017 | 0.050 | 0.732 |  | -0.055 | 0.050 | 0.265 |
| rs5770908-2 | 0.031 | 0.059 | 0.598 |  | 0.023 | 0.060 | 0.703 |  | 0.085 | 0.059 | 0.151 |
| rs5770908-1 | 0.002 | 0.037 | 0.958 |  | 0.021 | 0.037 | 0.566 |  | 0.037 | 0.037 | 0.316 |
| rs57696383-2 | -0.023 | 0.048 | 0.626 |  | 0.004 | 0.048 | 0.938 |  | -0.023 | 0.048 | 0.633 |
| rs57696383-1 | -0.026 | 0.041 | 0.527 |  | -0.036 | 0.041 | 0.387 |  | -0.067 | 0.041 | 0.101 |
| rs5754387-2 | -0.016 | 0.082 | 0.844 |  | -0.048 | 0.083 | 0.565 |  | -0.020 | 0.082 | 0.804 |
| rs5754387-1 | 0.012 | 0.037 | 0.741 |  | 0.008 | 0.037 | 0.836 |  | 0.025 | 0.037 | 0.504 |
| rs5746816-2 | 0.032 | 0.069 | 0.643 |  | 0.033 | 0.070 | 0.640 |  | -0.044 | 0.069 | 0.528 |
| rs5746816-1 | 0.004 | 0.070 | 0.959 |  | -0.016 | 0.071 | 0.818 |  | -0.003 | 0.070 | 0.967 |
| rs5735-2 | -0.088 | 0.062 | 0.152 |  | -0.102 | 0.062 | 0.099 |  | -0.090 | 0.062 | 0.146 |
| rs5735-1 | -0.058 | 0.036 | 0.106 |  | -0.030 | 0.036 | 0.402 |  | -0.035 | 0.036 | 0.334 |
| rs573335-2 | -0.048 | 0.212 | 0.819 |  | -0.030 | 0.213 | 0.889 |  | -0.003 | 0.211 | 0.987 |
| rs573335-1 | 0.081 | 0.217 | 0.711 |  | 0.040 | 0.218 | 0.854 |  | 0.069 | 0.216 | 0.749 |
| rs571356-2 | 0.012 | 0.057 | 0.831 |  | -0.014 | 0.057 | 0.810 |  | 0.035 | 0.057 | 0.541 |
| rs571356-1 | -0.038 | 0.036 | 0.284 |  | -0.001 | 0.036 | 0.972 |  | -0.015 | 0.036 | 0.685 |
| rs570639864-1 | -0.031 | 0.232 | 0.895 |  | -0.084 | 0.234 | 0.720 |  | -0.159 | 0.236 | 0.502 |
| rs57043009-2 | 0.083 | 0.123 | 0.500 |  | 0.193 | 0.123 | 0.118 |  | 0.065 | 0.122 | 0.594 |
| rs57043009-1 | 0.035 | 0.123 | 0.774 |  | 0.133 | 0.124 | 0.282 |  | 0.055 | 0.123 | 0.656 |
| rs56940811-2 | -0.072 | 0.172 | 0.674 |  | -0.128 | 0.173 | 0.457 |  | 0.193 | 0.172 | 0.262 |
| rs56940811-1 | -0.069 | 0.175 | 0.694 |  | -0.085 | 0.176 | 0.629 |  | 0.169 | 0.175 | 0.333 |
| rs56921221-2 | 0.187 | 0.136 | 0.169 |  | 0.208 | 0.136 | 0.126 |  | 0.116 | 0.135 | 0.392 |
| rs56921221-1 | 0.145 | 0.139 | 0.298 |  | 0.164 | 0.140 | 0.240 |  | 0.070 | 0.139 | 0.616 |
| rs568396270-2 | -0.619 | 0.407 | 0.128 |  | -0.584 | 0.408 | 0.153 |  | 0.044 | 0.405 | 0.913 |
| rs56782495-2 | 0.144 | 0.220 | 0.513 |  | 0.006 | 0.221 | 0.978 |  | -0.168 | 0.220 | 0.445 |
| rs56782495-1 | -0.076 | 0.049 | 0.121 |  | -0.068 | 0.049 | 0.167 |  | -0.083 | 0.049 | 0.088 |
| rs567068501-2 | 1.484 | 1.395 | 0.287 |  | 2.000 | 1.401 | 0.154 |  | 1.441 | 1.390 | 0.300 |
| rs567068501-1 | 0.138 | 0.176 | 0.436 |  | -0.039 | 0.177 | 0.827 |  | -0.082 | 0.176 | 0.640 |
| rs56682471-2 | 0.008 | 0.089 | 0.932 |  | -0.026 | 0.089 | 0.775 |  | 0.005 | 0.089 | 0.958 |
| rs56682471-1 | -0.004 | 0.088 | 0.961 |  | -0.020 | 0.088 | 0.825 |  | 0.049 | 0.088 | 0.580 |
| rs564979-2 | 0.039 | 0.093 | 0.677 |  | 0.093 | 0.093 | 0.320 |  | 0.064 | 0.093 | 0.491 |
| rs564979-1 | 0.055 | 0.095 | 0.563 |  | 0.082 | 0.095 | 0.387 |  | 0.020 | 0.094 | 0.830 |
| rs56389497-2 | -0.024 | 0.119 | 0.841 |  | 0.005 | 0.119 | 0.968 |  | 0.120 | 0.118 | 0.310 |
| rs56389497-1 | 0.037 | 0.039 | 0.340 |  | 0.033 | 0.039 | 0.398 |  | 0.008 | 0.038 | 0.832 |
| rs56363908-2 | 0.143 | 0.349 | 0.682 |  | -0.119 | 0.350 | 0.733 |  | 0.075 | 0.347 | 0.829 |
| rs56363908-1 | 0.040 | 0.352 | 0.909 |  | -0.167 | 0.354 | 0.637 |  | -0.001 | 0.351 | 0.997 |
| rs563406543-1 | 0.024 | 0.133 | 0.856 |  | 0.080 | 0.133 | 0.549 |  | -0.147 | 0.133 | 0.269 |
| rs56320441-2 | -0.040 | 0.171 | 0.815 |  | 0.098 | 0.172 | 0.569 |  | 0.401 | 0.171 | 0.019 |
| rs56320441-1 | 0.011 | 0.175 | 0.949 |  | 0.161 | 0.176 | 0.360 |  | 0.380 | 0.174 | 0.029 |
| rs56283745-2 | 0.008 | 0.075 | 0.911 |  | 0.036 | 0.075 | 0.636 |  | 0.027 | 0.075 | 0.718 |
| rs56283745-1 | 0.040 | 0.076 | 0.602 |  | 0.043 | 0.077 | 0.570 |  | 0.040 | 0.076 | 0.602 |
| rs56259139-2 | -0.221 | 0.194 | 0.255 |  | -0.121 | 0.194 | 0.533 |  | -0.327 | 0.193 | 0.091 |
| rs56259139-1 | -0.045 | 0.047 | 0.341 |  | -0.072 | 0.047 | 0.126 |  | -0.011 | 0.047 | 0.817 |
| rs56253836-2 | 0.131 | 0.049 | 0.008 |  | 0.136 | 0.050 | 0.006 |  | 0.096 | 0.049 | 0.050 |
| rs56253836-1 | 0.089 | 0.038 | 0.020 |  | 0.079 | 0.038 | 0.041 |  | 0.028 | 0.038 | 0.459 |
| rs56240884-2 | 0.061 | 0.065 | 0.346 |  | 0.030 | 0.065 | 0.647 |  | -0.004 | 0.064 | 0.950 |
| rs56240884-1 | 0.069 | 0.065 | 0.288 |  | 0.032 | 0.065 | 0.620 |  | -0.012 | 0.065 | 0.851 |
| rs56235417-2 | 0.129 | 0.123 | 0.294 |  | 0.110 | 0.123 | 0.372 |  | -0.076 | 0.122 | 0.536 |
| rs56235417-1 | 0.002 | 0.039 | 0.950 |  | -0.007 | 0.039 | 0.862 |  | 0.048 | 0.039 | 0.212 |
| rs56225285-2 | 0.020 | 0.069 | 0.769 |  | -0.021 | 0.069 | 0.757 |  | -0.002 | 0.069 | 0.975 |
| rs56225285-1 | -0.061 | 0.071 | 0.391 |  | -0.072 | 0.071 | 0.311 |  | -0.107 | 0.070 | 0.129 |
| rs56087288-2 | 0.014 | 0.063 | 0.820 |  | -0.015 | 0.063 | 0.810 |  | 0.036 | 0.063 | 0.568 |
| rs56087288-1 | -0.003 | 0.036 | 0.924 |  | 0.027 | 0.037 | 0.466 |  | 0.007 | 0.036 | 0.857 |
| rs56074687-2 | -0.032 | 0.070 | 0.643 |  | 0.070 | 0.070 | 0.319 |  | -0.010 | 0.069 | 0.881 |
| rs56074687-1 | 0.038 | 0.069 | 0.581 |  | 0.091 | 0.069 | 0.191 |  | 0.122 | 0.069 | 0.076 |
| rs56031625-2 | 0.005 | 0.087 | 0.952 |  | 0.032 | 0.087 | 0.715 |  | 0.091 | 0.087 | 0.297 |
| rs56031625-1 | -0.041 | 0.037 | 0.265 |  | -0.036 | 0.037 | 0.326 |  | -0.064 | 0.037 | 0.083 |
| rs560152536-2 | -0.275 | 0.154 | 0.075 |  | -0.185 | 0.155 | 0.232 |  | -0.129 | 0.154 | 0.404 |
| rs55983207-2 | 0.166 | 0.460 | 0.718 |  | 0.259 | 0.462 | 0.576 |  | 0.192 | 0.459 | 0.675 |
| rs55983207-1 | 0.481 | 0.465 | 0.301 |  | 0.484 | 0.467 | 0.300 |  | 0.384 | 0.464 | 0.408 |
| rs559693545-2 | 0.020 | 0.239 | 0.934 |  | -0.142 | 0.240 | 0.554 |  | -0.125 | 0.238 | 0.600 |
| rs55787537-2 | 0.086 | 0.102 | 0.400 |  | 0.111 | 0.102 | 0.277 |  | 0.130 | 0.102 | 0.202 |
| rs55787537-1 | -0.062 | 0.039 | 0.114 |  | -0.048 | 0.039 | 0.219 |  | 0.003 | 0.039 | 0.929 |
| rs557311052-1 | -0.347 | 0.165 | 0.036 |  | -0.446 | 0.166 | 0.007 |  | -0.061 | 0.165 | 0.711 |
| rs547251-2 | -0.087 | 0.057 | 0.125 |  | -0.100 | 0.057 | 0.082 |  | -0.081 | 0.057 | 0.152 |
| rs547251-1 | -0.041 | 0.036 | 0.254 |  | -0.006 | 0.036 | 0.859 |  | -0.060 | 0.036 | 0.099 |
| rs542247589-1 | 0.327 | 0.220 | 0.137 |  | 0.207 | 0.221 | 0.349 |  | -0.045 | 0.219 | 0.838 |
| rs536574218-1 | -0.339 | 0.269 | 0.207 |  | -0.401 | 0.270 | 0.137 |  | -0.167 | 0.268 | 0.533 |
| rs531968440-2 | 0.155 | 0.244 | 0.527 |  | 0.121 | 0.245 | 0.622 |  | 0.132 | 0.244 | 0.589 |
| rs4988321-2 | 0.316 | 0.355 | 0.374 |  | -0.047 | 0.357 | 0.896 |  | -0.402 | 0.354 | 0.257 |
| rs4988321-1 | -0.053 | 0.063 | 0.400 |  | -0.041 | 0.063 | 0.517 |  | -0.082 | 0.063 | 0.195 |
| rs4979905-2 | -0.124 | 0.086 | 0.150 |  | -0.085 | 0.087 | 0.326 |  | 0.115 | 0.086 | 0.181 |
| rs4979905-1 | -0.127 | 0.036 | 0.000 |  | -0.070 | 0.037 | 0.057 |  | 0.014 | 0.036 | 0.692 |
| rs4974186-2 | -0.074 | 0.049 | 0.132 |  | -0.079 | 0.049 | 0.111 |  | -0.047 | 0.049 | 0.341 |
| rs4974186-1 | -0.053 | 0.041 | 0.193 |  | -0.045 | 0.041 | 0.272 |  | 0.002 | 0.041 | 0.957 |
| rs4964511-2 | 0.069 | 0.049 | 0.159 |  | 0.040 | 0.049 | 0.416 |  | 0.041 | 0.049 | 0.404 |
| rs4964511-1 | 0.007 | 0.045 | 0.882 |  | 0.049 | 0.045 | 0.282 |  | -0.006 | 0.045 | 0.900 |
| rs4961733-2 | -0.014 | 0.072 | 0.842 |  | 0.011 | 0.072 | 0.881 |  | 0.061 | 0.072 | 0.398 |
| rs4961733-1 | -0.013 | 0.037 | 0.724 |  | -0.035 | 0.037 | 0.340 |  | -0.082 | 0.037 | 0.026 |
| rs4945829-2 | 0.026 | 0.056 | 0.640 |  | 0.016 | 0.056 | 0.778 |  | 0.020 | 0.056 | 0.715 |
| rs4945829-1 | 0.042 | 0.055 | 0.445 |  | 0.035 | 0.055 | 0.524 |  | 0.081 | 0.055 | 0.142 |
| rs4942901-2 | -0.005 | 0.072 | 0.943 |  | 0.019 | 0.072 | 0.792 |  | 0.001 | 0.072 | 0.983 |
| rs4942901-1 | 0.014 | 0.036 | 0.691 |  | 0.020 | 0.036 | 0.583 |  | 0.029 | 0.036 | 0.429 |
| rs4934253-2 | -0.351 | 0.303 | 0.247 |  | -0.409 | 0.305 | 0.180 |  | -0.437 | 0.302 | 0.149 |
| rs4934253-1 | -0.315 | 0.307 | 0.305 |  | -0.358 | 0.308 | 0.245 |  | -0.360 | 0.306 | 0.239 |
| rs4918760-2 | 0.004 | 0.052 | 0.939 |  | 0.083 | 0.052 | 0.113 |  | 0.017 | 0.052 | 0.737 |
| rs4918760-1 | 0.044 | 0.036 | 0.229 |  | 0.057 | 0.037 | 0.122 |  | 0.034 | 0.036 | 0.350 |
| rs4912085-2 | 0.013 | 0.051 | 0.801 |  | 0.038 | 0.051 | 0.456 |  | 0.004 | 0.051 | 0.942 |
| rs4912085-1 | -0.038 | 0.049 | 0.433 |  | -0.036 | 0.049 | 0.467 |  | -0.036 | 0.049 | 0.459 |
| rs4909323-2 | -0.019 | 0.051 | 0.711 |  | 0.045 | 0.052 | 0.386 |  | -0.030 | 0.051 | 0.552 |
| rs4909323-1 | -0.004 | 0.050 | 0.931 |  | 0.015 | 0.050 | 0.766 |  | 0.037 | 0.050 | 0.456 |
| rs4908776-2 | 0.100 | 0.085 | 0.241 |  | 0.095 | 0.086 | 0.269 |  | 0.037 | 0.086 | 0.665 |
| rs4908776-1 | 0.117 | 0.087 | 0.177 |  | 0.109 | 0.087 | 0.212 |  | 0.069 | 0.087 | 0.428 |
| rs4895901-2 | 0.012 | 0.059 | 0.835 |  | -0.006 | 0.059 | 0.917 |  | -0.095 | 0.059 | 0.107 |
| rs4895901-1 | -0.057 | 0.039 | 0.147 |  | -0.061 | 0.039 | 0.119 |  | -0.096 | 0.039 | 0.014 |
| rs4886659-2 | 0.022 | 0.066 | 0.742 |  | -0.045 | 0.067 | 0.498 |  | 0.030 | 0.066 | 0.649 |
| rs4886659-1 | 0.002 | 0.035 | 0.946 |  | 0.025 | 0.036 | 0.484 |  | 0.001 | 0.035 | 0.987 |
| rs4880735-2 | 0.106 | 0.090 | 0.237 |  | 0.048 | 0.090 | 0.592 |  | 0.057 | 0.090 | 0.523 |
| rs4880735-1 | 0.100 | 0.092 | 0.281 |  | 0.072 | 0.093 | 0.437 |  | 0.050 | 0.092 | 0.587 |
| rs4878008-2 | 0.020 | 0.054 | 0.704 |  | 0.054 | 0.054 | 0.324 |  | -0.047 | 0.054 | 0.387 |
| rs4878008-1 | -0.028 | 0.037 | 0.456 |  | -0.010 | 0.037 | 0.794 |  | -0.011 | 0.037 | 0.769 |
| rs4876858-2 | 0.097 | 0.076 | 0.205 |  | 0.070 | 0.077 | 0.358 |  | 0.051 | 0.076 | 0.500 |
| rs4876858-1 | 0.078 | 0.077 | 0.309 |  | 0.059 | 0.077 | 0.445 |  | 0.048 | 0.077 | 0.536 |
| rs4871827-2 | -0.104 | 0.057 | 0.069 |  | -0.130 | 0.057 | 0.023 |  | -0.049 | 0.057 | 0.385 |
| rs4871827-1 | -0.109 | 0.056 | 0.051 |  | -0.140 | 0.056 | 0.012 |  | -0.046 | 0.056 | 0.410 |
| rs4868145-2 | 0.004 | 0.052 | 0.942 |  | 0.015 | 0.053 | 0.778 |  | 0.033 | 0.052 | 0.525 |
| rs4868145-1 | -0.025 | 0.051 | 0.618 |  | 0.005 | 0.051 | 0.929 |  | 0.011 | 0.051 | 0.824 |
| rs4865610-2 | 0.102 | 0.139 | 0.464 |  | 0.052 | 0.139 | 0.708 |  | 0.034 | 0.138 | 0.803 |
| rs4865610-1 | -0.118 | 0.043 | 0.006 |  | -0.085 | 0.043 | 0.048 |  | -0.097 | 0.043 | 0.023 |
| rs4849701-2 | 0.040 | 0.051 | 0.428 |  | 0.053 | 0.051 | 0.299 |  | -0.002 | 0.051 | 0.961 |
| rs4849701-1 | 0.029 | 0.042 | 0.487 |  | 0.032 | 0.042 | 0.447 |  | 0.017 | 0.042 | 0.685 |
| rs4842700-2 | -0.134 | 0.096 | 0.163 |  | -0.150 | 0.097 | 0.119 |  | -0.035 | 0.096 | 0.716 |
| rs4842700-1 | -0.079 | 0.040 | 0.045 |  | -0.079 | 0.040 | 0.047 |  | -0.028 | 0.040 | 0.474 |
| rs482339-2 | -0.101 | 0.059 | 0.089 |  | -0.113 | 0.060 | 0.059 |  | -0.086 | 0.059 | 0.149 |
| rs482339-1 | -0.056 | 0.037 | 0.130 |  | -0.062 | 0.037 | 0.096 |  | -0.074 | 0.037 | 0.046 |
| rs4821797-2 | 0.067 | 0.056 | 0.231 |  | 0.029 | 0.056 | 0.599 |  | 0.088 | 0.055 | 0.110 |
| rs4821797-1 | -0.002 | 0.036 | 0.945 |  | 0.002 | 0.036 | 0.946 |  | 0.025 | 0.036 | 0.487 |
| rs4806862-2 | -0.041 | 0.055 | 0.455 |  | -0.018 | 0.055 | 0.741 |  | 0.030 | 0.055 | 0.589 |
| rs4806862-1 | -0.060 | 0.055 | 0.276 |  | -0.005 | 0.055 | 0.922 |  | 0.020 | 0.055 | 0.715 |
| rs4802111-2 | -0.132 | 0.049 | 0.007 |  | -0.107 | 0.049 | 0.029 |  | -0.109 | 0.049 | 0.026 |
| rs4802111-1 | -0.066 | 0.045 | 0.140 |  | -0.059 | 0.045 | 0.184 |  | -0.044 | 0.044 | 0.318 |
| rs478438-2 | -0.139 | 0.146 | 0.339 |  | -0.149 | 0.146 | 0.310 |  | -0.042 | 0.145 | 0.773 |
| rs478438-1 | -0.048 | 0.045 | 0.283 |  | -0.055 | 0.045 | 0.224 |  | 0.042 | 0.045 | 0.351 |
| rs4782351-2 | 0.018 | 0.051 | 0.723 |  | 0.064 | 0.051 | 0.205 |  | 0.057 | 0.050 | 0.257 |
| rs4782351-1 | 0.040 | 0.048 | 0.403 |  | 0.086 | 0.048 | 0.073 |  | 0.063 | 0.048 | 0.191 |
| rs4778075-2 | -0.037 | 0.078 | 0.635 |  | -0.060 | 0.078 | 0.446 |  | -0.032 | 0.078 | 0.684 |
| rs4778075-1 | -0.031 | 0.080 | 0.693 |  | -0.090 | 0.080 | 0.262 |  | 0.021 | 0.079 | 0.796 |
| rs4752678-2 | -0.081 | 0.049 | 0.099 |  | -0.116 | 0.049 | 0.019 |  | -0.169 | 0.049 | 0.001 |
| rs4752678-1 | -0.058 | 0.045 | 0.190 |  | -0.082 | 0.045 | 0.068 |  | -0.078 | 0.044 | 0.080 |
| rs4744295-2 | 0.007 | 0.052 | 0.888 |  | 0.043 | 0.052 | 0.415 |  | 0.009 | 0.052 | 0.862 |
| rs4744295-1 | -0.027 | 0.048 | 0.573 |  | -0.002 | 0.048 | 0.971 |  | -0.014 | 0.048 | 0.770 |
| rs4743930-2 | -0.099 | 0.067 | 0.136 |  | -0.071 | 0.067 | 0.289 |  | 0.002 | 0.066 | 0.972 |
| rs4743930-1 | -0.045 | 0.067 | 0.505 |  | -0.030 | 0.067 | 0.658 |  | 0.085 | 0.067 | 0.203 |
| rs4739697-2 | -0.134 | 0.058 | 0.020 |  | -0.092 | 0.058 | 0.113 |  | -0.027 | 0.058 | 0.635 |
| rs4739697-1 | -0.121 | 0.059 | 0.039 |  | -0.075 | 0.059 | 0.200 |  | -0.034 | 0.058 | 0.564 |
| rs4711750-2 | -0.033 | 0.049 | 0.496 |  | -0.016 | 0.049 | 0.742 |  | -0.021 | 0.049 | 0.672 |
| rs4711750-1 | -0.064 | 0.043 | 0.133 |  | -0.014 | 0.043 | 0.741 |  | -0.051 | 0.042 | 0.233 |
| rs4686419-2 | 0.037 | 0.060 | 0.537 |  | -0.028 | 0.061 | 0.646 |  | -0.009 | 0.060 | 0.876 |
| rs4686419-1 | 0.014 | 0.060 | 0.815 |  | -0.035 | 0.060 | 0.563 |  | -0.086 | 0.060 | 0.150 |
| rs4683184-2 | -0.079 | 0.054 | 0.148 |  | -0.105 | 0.055 | 0.055 |  | -0.071 | 0.054 | 0.191 |
| rs4683184-1 | -0.015 | 0.036 | 0.675 |  | -0.033 | 0.036 | 0.368 |  | -0.025 | 0.036 | 0.481 |
| rs4676276-2 | -0.067 | 0.049 | 0.171 |  | -0.045 | 0.049 | 0.367 |  | -0.037 | 0.049 | 0.457 |
| rs4676276-1 | 0.005 | 0.044 | 0.903 |  | 0.021 | 0.044 | 0.626 |  | -0.002 | 0.044 | 0.965 |
| rs4669522-2 | 0.119 | 0.095 | 0.212 |  | 0.153 | 0.096 | 0.110 |  | 0.034 | 0.095 | 0.722 |
| rs4669522-1 | -0.048 | 0.037 | 0.195 |  | -0.034 | 0.037 | 0.370 |  | -0.011 | 0.037 | 0.764 |
| rs4666343-2 | 0.202 | 0.070 | 0.004 |  | 0.246 | 0.071 | 0.001 |  | 0.105 | 0.070 | 0.136 |
| rs4666343-1 | 0.018 | 0.036 | 0.614 |  | 0.023 | 0.036 | 0.515 |  | -0.021 | 0.036 | 0.563 |
| rs4664604-2 | -0.052 | 0.089 | 0.555 |  | -0.127 | 0.089 | 0.156 |  | -0.090 | 0.089 | 0.308 |
| rs4664604-1 | 0.051 | 0.038 | 0.181 |  | 0.029 | 0.038 | 0.455 |  | 0.080 | 0.038 | 0.035 |
| rs4635400-2 | -0.015 | 0.055 | 0.785 |  | -0.018 | 0.055 | 0.752 |  | 0.004 | 0.055 | 0.936 |
| rs4635400-1 | 0.000 | 0.038 | 0.998 |  | 0.011 | 0.038 | 0.763 |  | -0.006 | 0.038 | 0.879 |
| rs4589253-2 | -0.074 | 0.051 | 0.143 |  | -0.066 | 0.051 | 0.191 |  | -0.120 | 0.050 | 0.017 |
| rs4589253-1 | -0.022 | 0.037 | 0.557 |  | -0.026 | 0.037 | 0.484 |  | -0.052 | 0.037 | 0.161 |
| rs4589135-2 | 0.077 | 0.052 | 0.143 |  | 0.001 | 0.053 | 0.981 |  | 0.053 | 0.052 | 0.308 |
| rs4589135-1 | 0.048 | 0.038 | 0.205 |  | -0.001 | 0.038 | 0.982 |  | -0.035 | 0.037 | 0.344 |
| rs45595933-2 | 0.002 | 0.108 | 0.983 |  | 0.081 | 0.108 | 0.454 |  | 0.017 | 0.108 | 0.873 |
| rs45595933-1 | 0.054 | 0.111 | 0.625 |  | 0.124 | 0.111 | 0.266 |  | 0.052 | 0.110 | 0.635 |
| rs45573936-2 | -0.508 | 1.206 | 0.674 |  | -0.785 | 1.211 | 0.517 |  | -0.142 | 1.202 | 0.906 |
| rs45573936-1 | -0.096 | 0.086 | 0.264 |  | -0.100 | 0.087 | 0.246 |  | -0.072 | 0.086 | 0.399 |
| rs45446698-2 | -0.945 | 0.544 | 0.082 |  | -0.666 | 0.547 | 0.223 |  | -0.258 | 0.543 | 0.635 |
| rs45446698-1 | -0.094 | 0.074 | 0.201 |  | -0.089 | 0.074 | 0.232 |  | -0.110 | 0.074 | 0.136 |
| rs4541111-2 | 0.018 | 0.049 | 0.720 |  | 0.001 | 0.049 | 0.989 |  | 0.014 | 0.049 | 0.772 |
| rs4541111-1 | -0.044 | 0.042 | 0.298 |  | -0.047 | 0.042 | 0.265 |  | -0.035 | 0.042 | 0.395 |
| rs4535826-2 | -0.075 | 0.075 | 0.319 |  | -0.115 | 0.075 | 0.125 |  | -0.081 | 0.075 | 0.277 |
| rs4535826-1 | -0.045 | 0.037 | 0.221 |  | -0.057 | 0.037 | 0.125 |  | 0.007 | 0.037 | 0.843 |
| rs4532510-2 | -0.100 | 0.049 | 0.041 |  | -0.051 | 0.049 | 0.299 |  | -0.050 | 0.049 | 0.304 |
| rs4532510-1 | -0.031 | 0.041 | 0.448 |  | -0.005 | 0.041 | 0.901 |  | 0.009 | 0.041 | 0.825 |
| rs4505759-2 | -0.056 | 0.071 | 0.435 |  | -0.087 | 0.072 | 0.225 |  | -0.032 | 0.071 | 0.657 |
| rs4505759-1 | -0.024 | 0.066 | 0.710 |  | -0.055 | 0.066 | 0.401 |  | 0.006 | 0.065 | 0.922 |
| rs4496284-2 | -0.038 | 0.050 | 0.445 |  | -0.061 | 0.051 | 0.225 |  | -0.051 | 0.050 | 0.309 |
| rs4496284-1 | 0.005 | 0.047 | 0.921 |  | -0.014 | 0.047 | 0.772 |  | -0.025 | 0.047 | 0.592 |
| rs4491596-2 | 0.048 | 0.075 | 0.520 |  | 0.028 | 0.075 | 0.709 |  | -0.097 | 0.075 | 0.194 |
| rs4491596-1 | -0.030 | 0.037 | 0.425 |  | -0.035 | 0.037 | 0.346 |  | -0.024 | 0.037 | 0.522 |
| rs4470366-2 | -0.055 | 0.049 | 0.270 |  | -0.035 | 0.050 | 0.484 |  | 0.005 | 0.049 | 0.914 |
| rs4470366-1 | -0.100 | 0.038 | 0.009 |  | -0.063 | 0.039 | 0.099 |  | -0.039 | 0.038 | 0.303 |
| rs4456244-2 | 0.018 | 0.049 | 0.707 |  | 0.015 | 0.049 | 0.760 |  | 0.074 | 0.049 | 0.132 |
| rs4456244-1 | -0.052 | 0.040 | 0.188 |  | -0.077 | 0.040 | 0.056 |  | 0.030 | 0.040 | 0.450 |
| rs4450871-2 | -0.019 | 0.049 | 0.699 |  | 0.010 | 0.049 | 0.843 |  | -0.026 | 0.049 | 0.600 |
| rs4450871-1 | -0.063 | 0.044 | 0.155 |  | -0.034 | 0.044 | 0.445 |  | -0.106 | 0.044 | 0.015 |
| rs4418639-2 | 0.006 | 0.052 | 0.913 |  | -0.014 | 0.052 | 0.790 |  | 0.034 | 0.052 | 0.512 |
| rs4418639-1 | -0.002 | 0.049 | 0.961 |  | -0.029 | 0.049 | 0.560 |  | 0.035 | 0.049 | 0.473 |
| rs4395467-2 | -0.053 | 0.049 | 0.274 |  | -0.064 | 0.049 | 0.190 |  | -0.047 | 0.049 | 0.330 |
| rs4395467-1 | -0.091 | 0.043 | 0.033 |  | -0.108 | 0.043 | 0.012 |  | -0.047 | 0.043 | 0.269 |
| rs437112-2 | -0.027 | 0.051 | 0.603 |  | 0.023 | 0.051 | 0.659 |  | -0.080 | 0.051 | 0.115 |
| rs437112-1 | -0.028 | 0.039 | 0.467 |  | -0.016 | 0.039 | 0.674 |  | -0.058 | 0.039 | 0.135 |
| rs4360494-2 | -0.045 | 0.049 | 0.357 |  | -0.019 | 0.049 | 0.701 |  | -0.016 | 0.049 | 0.748 |
| rs4360494-1 | 0.043 | 0.040 | 0.293 |  | 0.039 | 0.041 | 0.333 |  | 0.018 | 0.040 | 0.659 |
| rs4341955-2 | 0.141 | 0.078 | 0.072 |  | 0.130 | 0.079 | 0.098 |  | 0.029 | 0.078 | 0.712 |
| rs4341955-1 | 0.090 | 0.038 | 0.017 |  | 0.093 | 0.038 | 0.015 |  | 0.024 | 0.038 | 0.523 |
| rs42916-2 | -0.009 | 0.068 | 0.899 |  | 0.027 | 0.068 | 0.696 |  | -0.068 | 0.068 | 0.313 |
| rs42916-1 | 0.034 | 0.037 | 0.355 |  | 0.019 | 0.037 | 0.598 |  | -0.029 | 0.036 | 0.434 |
| rs4288197-2 | -0.397 | 0.281 | 0.159 |  | -0.418 | 0.283 | 0.139 |  | -0.524 | 0.281 | 0.062 |
| rs4288197-1 | -0.114 | 0.061 | 0.062 |  | -0.148 | 0.061 | 0.016 |  | -0.079 | 0.061 | 0.196 |
| rs4264571-2 | -0.111 | 0.065 | 0.089 |  | -0.140 | 0.066 | 0.033 |  | 0.032 | 0.065 | 0.620 |
| rs4264571-1 | -0.042 | 0.036 | 0.244 |  | -0.059 | 0.036 | 0.102 |  | 0.005 | 0.036 | 0.896 |
| rs4254591-2 | -0.037 | 0.056 | 0.509 |  | -0.031 | 0.056 | 0.577 |  | -0.061 | 0.056 | 0.270 |
| rs4254591-1 | -0.027 | 0.056 | 0.634 |  | -0.014 | 0.056 | 0.806 |  | -0.071 | 0.056 | 0.200 |
| rs4238686-2 | 0.079 | 0.051 | 0.119 |  | 0.047 | 0.051 | 0.359 |  | 0.102 | 0.051 | 0.044 |
| rs4238686-1 | 0.022 | 0.041 | 0.583 |  | 0.020 | 0.041 | 0.626 |  | 0.022 | 0.041 | 0.590 |
| rs4238428-2 | 0.005 | 0.049 | 0.925 |  | 0.020 | 0.049 | 0.686 |  | -0.052 | 0.048 | 0.282 |
| rs4238428-1 | 0.005 | 0.042 | 0.901 |  | 0.031 | 0.043 | 0.465 |  | 0.048 | 0.042 | 0.261 |
| rs4233949-2 | 0.077 | 0.054 | 0.151 |  | 0.046 | 0.054 | 0.388 |  | -0.037 | 0.053 | 0.485 |
| rs4233949-1 | 0.013 | 0.051 | 0.803 |  | -0.030 | 0.051 | 0.550 |  | 0.004 | 0.051 | 0.942 |
| rs42235-2 | -0.120 | 0.059 | 0.042 |  | -0.119 | 0.059 | 0.045 |  | -0.107 | 0.059 | 0.070 |
| rs42235-1 | -0.015 | 0.037 | 0.682 |  | -0.016 | 0.037 | 0.674 |  | 0.008 | 0.037 | 0.821 |
| rs4149083-2 | -0.029 | 0.111 | 0.794 |  | 0.028 | 0.112 | 0.803 |  | -0.038 | 0.111 | 0.734 |
| rs4149083-1 | -0.022 | 0.115 | 0.852 |  | 0.048 | 0.116 | 0.677 |  | -0.030 | 0.115 | 0.797 |
| rs4143691-2 | -0.118 | 0.056 | 0.034 |  | -0.075 | 0.056 | 0.181 |  | 0.005 | 0.056 | 0.934 |
| rs4143691-1 | -0.060 | 0.036 | 0.090 |  | -0.027 | 0.036 | 0.448 |  | -0.018 | 0.036 | 0.616 |
| rs41377546-2 | -0.065 | 0.102 | 0.527 |  | -0.141 | 0.103 | 0.168 |  | 0.008 | 0.102 | 0.934 |
| rs41377546-1 | -0.086 | 0.104 | 0.407 |  | -0.207 | 0.104 | 0.048 |  | -0.052 | 0.104 | 0.619 |
| rs41281637-1 | 0.214 | 0.133 | 0.107 |  | 0.166 | 0.134 | 0.216 |  | 0.281 | 0.133 | 0.034 |
| rs4081747-2 | 0.076 | 0.051 | 0.138 |  | 0.077 | 0.051 | 0.137 |  | 0.037 | 0.051 | 0.466 |
| rs4081747-1 | 0.033 | 0.049 | 0.504 |  | 0.043 | 0.049 | 0.376 |  | -0.028 | 0.049 | 0.561 |
| rs4075018-2 | -0.042 | 0.078 | 0.592 |  | 0.015 | 0.078 | 0.844 |  | 0.032 | 0.078 | 0.681 |
| rs4075018-1 | -0.015 | 0.080 | 0.847 |  | 0.030 | 0.080 | 0.704 |  | 0.028 | 0.080 | 0.728 |
| rs4074793-2 | 0.089 | 0.189 | 0.640 |  | -0.027 | 0.190 | 0.886 |  | -0.084 | 0.189 | 0.656 |
| rs4074793-1 | 0.098 | 0.194 | 0.613 |  | -0.048 | 0.195 | 0.807 |  | -0.007 | 0.193 | 0.970 |
| rs3971300-2 | -0.063 | 0.067 | 0.353 |  | -0.158 | 0.068 | 0.020 |  | -0.085 | 0.067 | 0.206 |
| rs3971300-1 | 0.011 | 0.038 | 0.784 |  | 0.025 | 0.038 | 0.514 |  | 0.018 | 0.038 | 0.633 |
| rs3966800-2 | -0.004 | 0.049 | 0.927 |  | -0.035 | 0.049 | 0.475 |  | -0.001 | 0.048 | 0.980 |
| rs3966800-1 | 0.009 | 0.041 | 0.833 |  | -0.011 | 0.042 | 0.794 |  | 0.035 | 0.041 | 0.394 |
| rs3951468-2 | 0.038 | 0.048 | 0.428 |  | 0.039 | 0.049 | 0.425 |  | -0.010 | 0.048 | 0.831 |
| rs3951468-1 | 0.058 | 0.041 | 0.156 |  | 0.030 | 0.041 | 0.462 |  | 0.056 | 0.041 | 0.175 |
| rs3901638-2 | 0.094 | 0.058 | 0.103 |  | 0.105 | 0.058 | 0.070 |  | 0.149 | 0.057 | 0.010 |
| rs3901638-1 | 0.017 | 0.036 | 0.633 |  | 0.032 | 0.036 | 0.376 |  | -0.015 | 0.036 | 0.674 |
| rs3858202-2 | 0.007 | 0.052 | 0.888 |  | 0.003 | 0.052 | 0.947 |  | 0.052 | 0.052 | 0.321 |
| rs3858202-1 | -0.001 | 0.038 | 0.973 |  | 0.002 | 0.038 | 0.953 |  | 0.030 | 0.038 | 0.419 |
| rs3848474-2 | 0.052 | 0.049 | 0.292 |  | 0.054 | 0.049 | 0.275 |  | 0.073 | 0.049 | 0.136 |
| rs3848474-1 | 0.106 | 0.045 | 0.020 |  | 0.109 | 0.046 | 0.017 |  | 0.064 | 0.045 | 0.155 |
| rs3847063-2 | -0.060 | 0.049 | 0.219 |  | -0.007 | 0.049 | 0.884 |  | -0.109 | 0.049 | 0.025 |
| rs3847063-1 | -0.012 | 0.040 | 0.772 |  | 0.021 | 0.040 | 0.610 |  | -0.077 | 0.040 | 0.056 |
| rs3829849-2 | -0.026 | 0.056 | 0.646 |  | -0.021 | 0.056 | 0.707 |  | 0.043 | 0.056 | 0.438 |
| rs3829849-1 | -0.011 | 0.055 | 0.835 |  | -0.010 | 0.055 | 0.856 |  | 0.019 | 0.055 | 0.729 |
| rs3829241-2 | 0.087 | 0.054 | 0.104 |  | 0.108 | 0.054 | 0.045 |  | 0.096 | 0.053 | 0.072 |
| rs3829241-1 | 0.061 | 0.052 | 0.237 |  | 0.078 | 0.052 | 0.133 |  | 0.036 | 0.052 | 0.491 |
| rs3819331-2 | 0.007 | 0.123 | 0.958 |  | -0.023 | 0.124 | 0.852 |  | 0.001 | 0.123 | 0.995 |
| rs3819331-1 | -0.070 | 0.041 | 0.087 |  | -0.102 | 0.041 | 0.013 |  | -0.069 | 0.041 | 0.090 |
| rs3815970-2 | 0.017 | 0.048 | 0.725 |  | 0.051 | 0.049 | 0.289 |  | 0.007 | 0.048 | 0.887 |
| rs3815970-1 | -0.043 | 0.044 | 0.319 |  | 0.022 | 0.044 | 0.610 |  | -0.013 | 0.044 | 0.767 |
| rs381349-2 | -0.006 | 0.140 | 0.966 |  | 0.052 | 0.141 | 0.714 |  | -0.127 | 0.140 | 0.363 |
| rs381349-1 | -0.009 | 0.043 | 0.841 |  | 0.022 | 0.043 | 0.610 |  | 0.002 | 0.043 | 0.964 |
| rs3801427-2 | -0.112 | 0.077 | 0.149 |  | -0.061 | 0.078 | 0.434 |  | -0.032 | 0.077 | 0.676 |
| rs3801427-1 | 0.004 | 0.038 | 0.921 |  | 0.003 | 0.039 | 0.939 |  | 0.067 | 0.038 | 0.081 |
| rs3790608-2 | -0.172 | 0.117 | 0.144 |  | -0.036 | 0.118 | 0.758 |  | -0.022 | 0.117 | 0.850 |
| rs3790608-1 | -0.141 | 0.121 | 0.244 |  | 0.004 | 0.121 | 0.976 |  | 0.032 | 0.120 | 0.789 |
| rs3783449-2 | 0.123 | 0.148 | 0.405 |  | 0.218 | 0.149 | 0.143 |  | -0.002 | 0.148 | 0.991 |
| rs3783449-1 | 0.001 | 0.043 | 0.972 |  | 0.015 | 0.043 | 0.729 |  | -0.035 | 0.042 | 0.411 |
| rs3779456-2 | 0.097 | 0.051 | 0.056 |  | 0.067 | 0.051 | 0.185 |  | 0.050 | 0.051 | 0.318 |
| rs3779456-1 | 0.007 | 0.039 | 0.862 |  | -0.042 | 0.039 | 0.283 |  | -0.007 | 0.039 | 0.850 |
| rs3779381-2 | -0.159 | 0.093 | 0.089 |  | -0.127 | 0.094 | 0.176 |  | -0.302 | 0.093 | 0.001 |
| rs3779381-1 | -0.070 | 0.084 | 0.401 |  | -0.027 | 0.084 | 0.751 |  | -0.169 | 0.083 | 0.043 |
| rs3775770-2 | -0.096 | 0.066 | 0.147 |  | -0.075 | 0.066 | 0.261 |  | -0.072 | 0.066 | 0.277 |
| rs3775770-1 | 0.000 | 0.036 | 0.994 |  | 0.014 | 0.036 | 0.690 |  | -0.021 | 0.036 | 0.561 |
| rs3765971-2 | 0.048 | 0.060 | 0.424 |  | 0.053 | 0.061 | 0.382 |  | 0.052 | 0.060 | 0.386 |
| rs3765971-1 | -0.096 | 0.036 | 0.009 |  | -0.077 | 0.037 | 0.037 |  | -0.048 | 0.036 | 0.191 |
| rs3763745-2 | -0.018 | 0.075 | 0.814 |  | 0.055 | 0.075 | 0.462 |  | 0.000 | 0.075 | 0.995 |
| rs3763745-1 | 0.033 | 0.077 | 0.670 |  | 0.084 | 0.078 | 0.277 |  | 0.037 | 0.077 | 0.627 |
| rs3760456-2 | 0.037 | 0.049 | 0.454 |  | 0.040 | 0.050 | 0.425 |  | -0.049 | 0.049 | 0.315 |
| rs3760456-1 | 0.032 | 0.038 | 0.402 |  | 0.001 | 0.038 | 0.983 |  | -0.009 | 0.038 | 0.809 |
| rs3759549-2 | -0.056 | 0.052 | 0.279 |  | -0.076 | 0.052 | 0.145 |  | -0.058 | 0.052 | 0.265 |
| rs3759549-1 | -0.006 | 0.044 | 0.899 |  | -0.056 | 0.044 | 0.207 |  | 0.029 | 0.044 | 0.507 |
| rs3754855-2 | -0.101 | 0.054 | 0.060 |  | -0.117 | 0.054 | 0.030 |  | -0.094 | 0.054 | 0.078 |
| rs3754855-1 | -0.065 | 0.049 | 0.190 |  | -0.084 | 0.050 | 0.090 |  | -0.081 | 0.049 | 0.101 |
| rs3751745-2 | 0.037 | 0.160 | 0.819 |  | 0.093 | 0.160 | 0.563 |  | -0.069 | 0.159 | 0.663 |
| rs3751745-1 | -0.014 | 0.165 | 0.932 |  | 0.011 | 0.166 | 0.949 |  | -0.151 | 0.165 | 0.360 |
| rs3740861-2 | -0.075 | 0.063 | 0.231 |  | -0.049 | 0.063 | 0.437 |  | -0.091 | 0.063 | 0.145 |
| rs3740861-1 | -0.057 | 0.064 | 0.374 |  | -0.058 | 0.065 | 0.373 |  | -0.075 | 0.064 | 0.246 |
| rs370387-2 | -0.115 | 0.049 | 0.018 |  | -0.110 | 0.049 | 0.024 |  | -0.122 | 0.049 | 0.012 |
| rs370387-1 | -0.066 | 0.040 | 0.098 |  | -0.071 | 0.040 | 0.077 |  | -0.076 | 0.040 | 0.058 |
| rs368510-2 | -0.025 | 0.057 | 0.663 |  | -0.053 | 0.058 | 0.362 |  | -0.029 | 0.057 | 0.613 |
| rs368510-1 | 0.012 | 0.057 | 0.826 |  | -0.047 | 0.057 | 0.410 |  | -0.054 | 0.057 | 0.339 |
| rs368222845-1 | 0.130 | 0.226 | 0.567 |  | -0.104 | 0.227 | 0.648 |  | -0.011 | 0.226 | 0.961 |
| rs367166-2 | -0.046 | 0.140 | 0.743 |  | -0.008 | 0.141 | 0.953 |  | -0.103 | 0.140 | 0.460 |
| rs367166-1 | -0.033 | 0.043 | 0.437 |  | -0.007 | 0.043 | 0.871 |  | 0.024 | 0.043 | 0.570 |
| rs36066545-2 | -0.035 | 0.114 | 0.757 |  | 0.009 | 0.115 | 0.937 |  | 0.227 | 0.114 | 0.046 |
| rs36066545-1 | -0.022 | 0.116 | 0.849 |  | 0.034 | 0.116 | 0.771 |  | 0.213 | 0.116 | 0.066 |
| rs36027301-2 | 0.214 | 0.271 | 0.431 |  | 0.163 | 0.273 | 0.550 |  | 0.044 | 0.270 | 0.872 |
| rs36027301-1 | 0.131 | 0.275 | 0.635 |  | 0.036 | 0.276 | 0.897 |  | 0.172 | 0.274 | 0.531 |
| rs36016056-2 | -0.012 | 0.076 | 0.871 |  | -0.064 | 0.077 | 0.405 |  | 0.060 | 0.076 | 0.427 |
| rs36016056-1 | -0.010 | 0.076 | 0.894 |  | -0.090 | 0.077 | 0.243 |  | 0.023 | 0.076 | 0.759 |
| rs36010930-2 | -0.140 | 0.062 | 0.024 |  | -0.148 | 0.062 | 0.018 |  | -0.042 | 0.062 | 0.497 |
| rs36010930-1 | -0.081 | 0.062 | 0.195 |  | -0.091 | 0.063 | 0.147 |  | -0.007 | 0.062 | 0.909 |
| rs35988618-2 | -0.038 | 0.064 | 0.553 |  | -0.044 | 0.064 | 0.497 |  | -0.038 | 0.064 | 0.550 |
| rs35988618-1 | 0.020 | 0.036 | 0.585 |  | 0.041 | 0.036 | 0.256 |  | 0.005 | 0.036 | 0.887 |
| rs35966827-2 | -0.180 | 0.913 | 0.844 |  | -0.679 | 0.917 | 0.459 |  | -1.427 | 0.910 | 0.117 |
| rs35966827-1 | -0.184 | 0.916 | 0.841 |  | -0.698 | 0.921 | 0.448 |  | -1.344 | 0.914 | 0.141 |
| rs35881190-2 | -0.020 | 0.054 | 0.714 |  | 0.009 | 0.054 | 0.866 |  | 0.006 | 0.054 | 0.913 |
| rs35881190-1 | -0.040 | 0.036 | 0.274 |  | -0.048 | 0.036 | 0.187 |  | -0.031 | 0.036 | 0.389 |
| rs35717986-2 | 0.372 | 0.230 | 0.106 |  | 0.352 | 0.231 | 0.128 |  | 0.337 | 0.229 | 0.142 |
| rs35717986-1 | 0.316 | 0.234 | 0.177 |  | 0.315 | 0.235 | 0.180 |  | 0.345 | 0.233 | 0.139 |
| rs35531047-2 | 0.003 | 0.072 | 0.972 |  | 0.080 | 0.073 | 0.269 |  | 0.039 | 0.072 | 0.588 |
| rs35531047-1 | -0.042 | 0.073 | 0.565 |  | 0.064 | 0.073 | 0.379 |  | -0.022 | 0.073 | 0.763 |
| rs35392886-2 | -0.039 | 0.052 | 0.458 |  | -0.050 | 0.052 | 0.334 |  | -0.083 | 0.052 | 0.111 |
| rs35392886-1 | 0.031 | 0.050 | 0.539 |  | 0.016 | 0.050 | 0.750 |  | -0.018 | 0.050 | 0.725 |
| rs35329209-2 | 0.149 | 0.172 | 0.385 |  | 0.173 | 0.172 | 0.315 |  | 0.112 | 0.171 | 0.514 |
| rs35329209-1 | 0.102 | 0.176 | 0.560 |  | 0.143 | 0.177 | 0.419 |  | 0.079 | 0.176 | 0.653 |
| rs35308216-2 | 0.031 | 0.174 | 0.859 |  | -0.022 | 0.174 | 0.899 |  | -0.015 | 0.173 | 0.929 |
| rs35308216-1 | 0.028 | 0.051 | 0.582 |  | 0.017 | 0.051 | 0.737 |  | 0.025 | 0.051 | 0.620 |
| rs35264941-2 | 0.097 | 0.613 | 0.874 |  | 0.032 | 0.616 | 0.958 |  | 0.690 | 0.611 | 0.259 |
| rs35264941-1 | 0.030 | 0.091 | 0.737 |  | -0.031 | 0.091 | 0.734 |  | -0.133 | 0.090 | 0.142 |
| rs35223785-2 | 0.079 | 0.076 | 0.298 |  | 0.044 | 0.076 | 0.560 |  | -0.151 | 0.075 | 0.045 |
| rs35223785-1 | 0.097 | 0.077 | 0.207 |  | 0.045 | 0.077 | 0.564 |  | -0.139 | 0.076 | 0.070 |
| rs34811474-2 | -0.043 | 0.084 | 0.607 |  | 0.043 | 0.084 | 0.609 |  | -0.055 | 0.084 | 0.509 |
| rs34811474-1 | -0.019 | 0.037 | 0.599 |  | -0.004 | 0.037 | 0.924 |  | -0.018 | 0.036 | 0.612 |
| rs34804482-2 | -0.581 | 0.552 | 0.293 |  | -0.986 | 0.555 | 0.076 |  | -0.430 | 0.551 | 0.435 |
| rs34804482-1 | -0.086 | 0.090 | 0.341 |  | -0.126 | 0.091 | 0.163 |  | -0.212 | 0.090 | 0.019 |
| rs34780912-2 | -0.047 | 0.053 | 0.375 |  | -0.038 | 0.053 | 0.467 |  | -0.049 | 0.053 | 0.349 |
| rs34780912-1 | -0.065 | 0.051 | 0.208 |  | -0.063 | 0.052 | 0.219 |  | -0.091 | 0.051 | 0.075 |
| rs34778574-2 | 0.317 | 0.852 | 0.710 |  | 0.025 | 0.856 | 0.977 |  | -0.093 | 0.849 | 0.913 |
| rs34778574-1 | -0.139 | 0.076 | 0.066 |  | -0.164 | 0.076 | 0.032 |  | -0.175 | 0.076 | 0.021 |
| rs346588-2 | -0.150 | 0.099 | 0.130 |  | -0.145 | 0.099 | 0.146 |  | -0.190 | 0.099 | 0.054 |
| rs346588-1 | -0.058 | 0.039 | 0.135 |  | -0.029 | 0.039 | 0.448 |  | -0.039 | 0.039 | 0.314 |
| rs34587622-2 | -0.221 | 0.156 | 0.158 |  | -0.284 | 0.157 | 0.070 |  | -0.131 | 0.156 | 0.400 |
| rs34587622-1 | -0.040 | 0.044 | 0.353 |  | -0.012 | 0.044 | 0.787 |  | 0.031 | 0.043 | 0.479 |
| rs34583478-2 | -0.363 | 0.262 | 0.165 |  | -0.443 | 0.263 | 0.092 |  | -0.460 | 0.261 | 0.078 |
| rs34583478-1 | -0.052 | 0.054 | 0.331 |  | -0.066 | 0.054 | 0.226 |  | -0.032 | 0.054 | 0.552 |
| rs34553872-2 | -0.226 | 0.098 | 0.021 |  | -0.234 | 0.098 | 0.018 |  | -0.374 | 0.098 | 0.000 |
| rs34553872-1 | -0.115 | 0.100 | 0.254 |  | -0.132 | 0.101 | 0.190 |  | -0.219 | 0.100 | 0.029 |
| rs344078-2 | -0.184 | 0.088 | 0.038 |  | -0.143 | 0.089 | 0.108 |  | -0.197 | 0.088 | 0.025 |
| rs344078-1 | -0.003 | 0.038 | 0.934 |  | 0.030 | 0.038 | 0.427 |  | -0.028 | 0.037 | 0.451 |
| rs343994-2 | -0.045 | 0.051 | 0.370 |  | -0.099 | 0.051 | 0.053 |  | 0.003 | 0.051 | 0.955 |
| rs343994-1 | 0.008 | 0.038 | 0.842 |  | -0.013 | 0.038 | 0.737 |  | 0.023 | 0.038 | 0.555 |
| rs34396633-2 | -0.447 | 0.841 | 0.595 |  | -0.364 | 0.844 | 0.666 |  | 0.661 | 0.838 | 0.431 |
| rs34396633-1 | -0.508 | 0.847 | 0.549 |  | -0.496 | 0.851 | 0.560 |  | 0.560 | 0.845 | 0.508 |
| rs34365165-2 | -0.045 | 0.097 | 0.641 |  | 0.015 | 0.097 | 0.874 |  | 0.039 | 0.096 | 0.689 |
| rs34365165-1 | -0.028 | 0.038 | 0.455 |  | -0.024 | 0.038 | 0.519 |  | 0.003 | 0.037 | 0.941 |
| rs34324915-2 | -0.136 | 0.058 | 0.019 |  | -0.136 | 0.058 | 0.019 |  | -0.041 | 0.058 | 0.481 |
| rs34324915-1 | -0.105 | 0.057 | 0.068 |  | -0.104 | 0.058 | 0.072 |  | -0.074 | 0.057 | 0.197 |
| rs34320498-2 | -0.129 | 0.106 | 0.224 |  | -0.103 | 0.106 | 0.331 |  | 0.085 | 0.105 | 0.419 |
| rs34320498-1 | 0.033 | 0.039 | 0.392 |  | 0.036 | 0.039 | 0.347 |  | 0.054 | 0.038 | 0.158 |
| rs34308190-2 | -0.063 | 0.215 | 0.769 |  | -0.137 | 0.216 | 0.524 |  | 0.232 | 0.214 | 0.279 |
| rs34308190-1 | -0.077 | 0.218 | 0.725 |  | -0.190 | 0.219 | 0.387 |  | 0.301 | 0.218 | 0.167 |
| rs34233878-2 | -0.014 | 0.170 | 0.936 |  | -0.076 | 0.170 | 0.654 |  | -0.055 | 0.169 | 0.744 |
| rs34233878-1 | -0.019 | 0.045 | 0.669 |  | 0.017 | 0.045 | 0.701 |  | 0.024 | 0.044 | 0.585 |
| rs34123233-2 | -0.144 | 0.091 | 0.112 |  | -0.127 | 0.091 | 0.166 |  | -0.034 | 0.091 | 0.707 |
| rs34123233-1 | -0.125 | 0.093 | 0.179 |  | -0.125 | 0.093 | 0.180 |  | 0.003 | 0.092 | 0.978 |
| rs33949355-2 | -0.040 | 0.047 | 0.395 |  | -0.049 | 0.048 | 0.306 |  | 0.005 | 0.047 | 0.922 |
| rs33949355-1 | -0.080 | 0.039 | 0.043 |  | -0.081 | 0.040 | 0.040 |  | -0.035 | 0.039 | 0.371 |
| rs327102-2 | -0.023 | 0.095 | 0.805 |  | -0.085 | 0.095 | 0.369 |  | -0.079 | 0.094 | 0.405 |
| rs327102-1 | -0.054 | 0.095 | 0.571 |  | -0.104 | 0.096 | 0.278 |  | -0.044 | 0.095 | 0.642 |
| rs3212240-2 | -0.005 | 0.049 | 0.926 |  | -0.038 | 0.049 | 0.442 |  | 0.032 | 0.049 | 0.517 |
| rs3212240-1 | 0.012 | 0.040 | 0.758 |  | 0.025 | 0.040 | 0.537 |  | -0.049 | 0.040 | 0.222 |
| rs314675-2 | 0.004 | 0.156 | 0.978 |  | -0.015 | 0.157 | 0.925 |  | -0.080 | 0.156 | 0.609 |
| rs314675-1 | 0.044 | 0.042 | 0.292 |  | 0.048 | 0.042 | 0.253 |  | 0.035 | 0.041 | 0.400 |
| rs3136302-2 | -0.074 | 0.050 | 0.144 |  | -0.063 | 0.051 | 0.211 |  | -0.123 | 0.050 | 0.015 |
| rs3136302-1 | -0.002 | 0.040 | 0.955 |  | 0.012 | 0.040 | 0.761 |  | -0.033 | 0.040 | 0.401 |
| rs3127084-2 | -0.057 | 0.049 | 0.246 |  | -0.041 | 0.049 | 0.407 |  | -0.086 | 0.049 | 0.081 |
| rs3127084-1 | 0.007 | 0.043 | 0.869 |  | 0.015 | 0.044 | 0.733 |  | -0.006 | 0.043 | 0.890 |
| rs3118906-2 | 0.144 | 0.069 | 0.037 |  | 0.099 | 0.069 | 0.155 |  | 0.101 | 0.069 | 0.143 |
| rs3118906-1 | 0.094 | 0.070 | 0.178 |  | 0.064 | 0.070 | 0.360 |  | 0.107 | 0.069 | 0.124 |
| rs3012465-2 | -0.070 | 0.055 | 0.207 |  | -0.056 | 0.056 | 0.313 |  | -0.094 | 0.055 | 0.090 |
| rs3012465-1 | -0.117 | 0.053 | 0.028 |  | -0.114 | 0.053 | 0.032 |  | -0.144 | 0.053 | 0.006 |
| rs299371-2 | 0.114 | 0.057 | 0.046 |  | 0.100 | 0.057 | 0.080 |  | 0.103 | 0.057 | 0.069 |
| rs299371-1 | 0.100 | 0.057 | 0.077 |  | 0.091 | 0.057 | 0.110 |  | 0.090 | 0.057 | 0.113 |
| rs2982573-2 | -0.031 | 0.055 | 0.566 |  | -0.028 | 0.055 | 0.611 |  | -0.101 | 0.055 | 0.064 |
| rs2982573-1 | 0.016 | 0.049 | 0.742 |  | 0.009 | 0.050 | 0.852 |  | -0.073 | 0.049 | 0.137 |
| rs2971879-2 | -0.151 | 0.074 | 0.043 |  | -0.164 | 0.075 | 0.028 |  | -0.105 | 0.074 | 0.155 |
| rs2971879-1 | 0.054 | 0.040 | 0.174 |  | 0.007 | 0.040 | 0.868 |  | -0.037 | 0.040 | 0.358 |
| rs2944590-2 | 0.007 | 0.049 | 0.890 |  | -0.025 | 0.050 | 0.620 |  | -0.012 | 0.049 | 0.800 |
| rs2944590-1 | 0.040 | 0.046 | 0.388 |  | 0.010 | 0.046 | 0.834 |  | -0.010 | 0.046 | 0.833 |
| rs2938697-2 | 0.014 | 0.060 | 0.809 |  | 0.016 | 0.060 | 0.791 |  | -0.028 | 0.060 | 0.644 |
| rs2938697-1 | -0.009 | 0.060 | 0.876 |  | 0.013 | 0.061 | 0.830 |  | -0.059 | 0.060 | 0.327 |
| rs2929308-2 | -0.068 | 0.051 | 0.189 |  | -0.081 | 0.052 | 0.115 |  | -0.076 | 0.051 | 0.137 |
| rs2929308-1 | -0.022 | 0.043 | 0.611 |  | -0.030 | 0.044 | 0.492 |  | -0.055 | 0.043 | 0.204 |
| rs2925049-2 | -0.008 | 0.049 | 0.875 |  | 0.004 | 0.049 | 0.941 |  | -0.054 | 0.049 | 0.270 |
| rs2925049-1 | -0.011 | 0.040 | 0.790 |  | -0.014 | 0.040 | 0.725 |  | -0.041 | 0.040 | 0.303 |
| rs2908574-2 | -0.267 | 0.174 | 0.125 |  | -0.370 | 0.175 | 0.034 |  | -0.116 | 0.173 | 0.504 |
| rs2908574-1 | 0.003 | 0.046 | 0.941 |  | 0.032 | 0.047 | 0.492 |  | -0.060 | 0.046 | 0.193 |
| rs2899472-2 | -0.065 | 0.076 | 0.393 |  | -0.070 | 0.076 | 0.360 |  | -0.067 | 0.076 | 0.380 |
| rs2899472-1 | -0.059 | 0.075 | 0.434 |  | -0.104 | 0.076 | 0.170 |  | -0.021 | 0.075 | 0.782 |
| rs2888814-2 | -0.049 | 0.053 | 0.355 |  | -0.009 | 0.054 | 0.860 |  | 0.041 | 0.053 | 0.443 |
| rs2888814-1 | -0.002 | 0.050 | 0.972 |  | 0.049 | 0.051 | 0.331 |  | 0.040 | 0.050 | 0.430 |
| rs28840973-2 | -0.236 | 0.187 | 0.207 |  | -0.256 | 0.188 | 0.173 |  | -0.232 | 0.186 | 0.214 |
| rs28840973-1 | -0.251 | 0.190 | 0.186 |  | -0.277 | 0.191 | 0.146 |  | -0.220 | 0.189 | 0.244 |
| rs28732148-2 | 0.139 | 0.202 | 0.492 |  | 0.129 | 0.203 | 0.526 |  | 0.007 | 0.201 | 0.974 |
| rs28732148-1 | 0.138 | 0.205 | 0.503 |  | 0.140 | 0.206 | 0.497 |  | 0.023 | 0.205 | 0.910 |
| rs28729182-2 | -0.228 | 0.156 | 0.144 |  | -0.102 | 0.157 | 0.514 |  | 0.003 | 0.155 | 0.986 |
| rs28729182-1 | -0.267 | 0.159 | 0.094 |  | -0.120 | 0.160 | 0.453 |  | -0.070 | 0.159 | 0.660 |
| rs28665840-2 | 0.027 | 0.083 | 0.743 |  | 0.108 | 0.084 | 0.198 |  | -0.029 | 0.083 | 0.731 |
| rs28665840-1 | -0.023 | 0.037 | 0.529 |  | -0.012 | 0.037 | 0.738 |  | 0.012 | 0.037 | 0.745 |
| rs28626308-2 | -0.096 | 0.311 | 0.759 |  | -0.207 | 0.312 | 0.508 |  | -0.076 | 0.310 | 0.805 |
| rs28626308-1 | 0.021 | 0.309 | 0.947 |  | -0.125 | 0.311 | 0.687 |  | 0.007 | 0.308 | 0.981 |
| rs28498618-2 | 0.174 | 0.085 | 0.041 |  | 0.145 | 0.086 | 0.092 |  | 0.160 | 0.085 | 0.060 |
| rs28498618-1 | 0.014 | 0.036 | 0.706 |  | -0.002 | 0.037 | 0.950 |  | 0.000 | 0.036 | 0.998 |
| rs2846894-2 | 0.128 | 0.104 | 0.218 |  | 0.073 | 0.104 | 0.487 |  | 0.171 | 0.104 | 0.100 |
| rs2846894-1 | 0.229 | 0.106 | 0.032 |  | 0.181 | 0.107 | 0.091 |  | 0.309 | 0.106 | 0.004 |
| rs2846444-2 | -0.071 | 0.052 | 0.172 |  | -0.102 | 0.052 | 0.050 |  | -0.111 | 0.052 | 0.031 |
| rs2846444-1 | -0.023 | 0.040 | 0.559 |  | -0.049 | 0.040 | 0.216 |  | -0.033 | 0.040 | 0.408 |
| rs28451064-2 | 0.213 | 0.146 | 0.145 |  | 0.275 | 0.147 | 0.061 |  | 0.070 | 0.146 | 0.631 |
| rs28451064-1 | -0.021 | 0.042 | 0.622 |  | -0.033 | 0.042 | 0.427 |  | -0.046 | 0.042 | 0.266 |
| rs284200-2 | -0.111 | 0.053 | 0.036 |  | -0.085 | 0.053 | 0.110 |  | 0.003 | 0.053 | 0.950 |
| rs284200-1 | 0.002 | 0.037 | 0.958 |  | -0.001 | 0.037 | 0.982 |  | 0.000 | 0.037 | 0.998 |
| rs28405309-2 | 0.027 | 0.049 | 0.579 |  | -0.005 | 0.049 | 0.920 |  | -0.009 | 0.049 | 0.859 |
| rs28405309-1 | 0.069 | 0.041 | 0.094 |  | 0.047 | 0.041 | 0.260 |  | -0.010 | 0.041 | 0.815 |
| rs28373428-2 | -0.026 | 0.105 | 0.805 |  | -0.012 | 0.106 | 0.911 |  | -0.145 | 0.105 | 0.168 |
| rs28373428-1 | 0.019 | 0.108 | 0.859 |  | 0.004 | 0.108 | 0.974 |  | -0.069 | 0.108 | 0.521 |
| rs2836613-2 | 0.237 | 0.076 | 0.002 |  | 0.249 | 0.076 | 0.001 |  | 0.213 | 0.076 | 0.005 |
| rs2836613-1 | 0.062 | 0.036 | 0.085 |  | 0.043 | 0.036 | 0.232 |  | 0.012 | 0.036 | 0.742 |
| rs28364580-2 | -0.139 | 0.071 | 0.052 |  | -0.101 | 0.072 | 0.161 |  | -0.137 | 0.071 | 0.055 |
| rs28364580-1 | -0.038 | 0.036 | 0.293 |  | -0.006 | 0.036 | 0.864 |  | 0.006 | 0.036 | 0.875 |
| rs28362709-2 | -0.058 | 0.080 | 0.471 |  | -0.057 | 0.081 | 0.479 |  | -0.261 | 0.080 | 0.001 |
| rs28362709-1 | -0.031 | 0.037 | 0.406 |  | -0.012 | 0.037 | 0.751 |  | -0.035 | 0.037 | 0.345 |
| rs283338-2 | 0.043 | 0.084 | 0.607 |  | -0.010 | 0.084 | 0.909 |  | -0.023 | 0.084 | 0.789 |
| rs283338-1 | 0.018 | 0.038 | 0.625 |  | 0.033 | 0.038 | 0.385 |  | -0.039 | 0.037 | 0.299 |
| rs2830913-2 | -0.124 | 0.049 | 0.012 |  | -0.099 | 0.050 | 0.045 |  | -0.126 | 0.049 | 0.011 |
| rs2830913-1 | -0.114 | 0.047 | 0.015 |  | -0.117 | 0.047 | 0.013 |  | -0.113 | 0.046 | 0.015 |
| rs2820501-2 | 0.487 | 0.279 | 0.081 |  | 0.541 | 0.280 | 0.053 |  | 0.375 | 0.278 | 0.176 |
| rs2820501-1 | 0.481 | 0.283 | 0.089 |  | 0.515 | 0.284 | 0.070 |  | 0.387 | 0.282 | 0.170 |
| rs2813495-2 | -0.063 | 0.084 | 0.452 |  | -0.007 | 0.085 | 0.930 |  | 0.025 | 0.084 | 0.766 |
| rs2813495-1 | -0.030 | 0.037 | 0.422 |  | -0.003 | 0.037 | 0.945 |  | 0.002 | 0.037 | 0.960 |
| rs2799098-2 | -0.129 | 0.092 | 0.163 |  | -0.109 | 0.093 | 0.241 |  | -0.143 | 0.092 | 0.122 |
| rs2799098-1 | 0.002 | 0.037 | 0.961 |  | -0.006 | 0.037 | 0.863 |  | 0.002 | 0.037 | 0.958 |
| rs2791655-2 | -0.049 | 0.063 | 0.430 |  | -0.073 | 0.063 | 0.249 |  | -0.080 | 0.063 | 0.204 |
| rs2791655-1 | -0.026 | 0.036 | 0.461 |  | -0.045 | 0.036 | 0.210 |  | -0.075 | 0.036 | 0.034 |
| rs2791559-2 | -0.022 | 0.051 | 0.668 |  | -0.028 | 0.051 | 0.593 |  | -0.078 | 0.051 | 0.129 |
| rs2791559-1 | -0.023 | 0.047 | 0.626 |  | -0.008 | 0.047 | 0.864 |  | -0.066 | 0.047 | 0.158 |
| rs2761884-2 | 0.101 | 0.064 | 0.116 |  | 0.125 | 0.064 | 0.052 |  | 0.176 | 0.064 | 0.006 |
| rs2761884-1 | 0.072 | 0.045 | 0.109 |  | 0.075 | 0.045 | 0.096 |  | 0.117 | 0.045 | 0.010 |
| rs2745599-2 | 0.084 | 0.049 | 0.089 |  | 0.039 | 0.050 | 0.438 |  | 0.068 | 0.049 | 0.168 |
| rs2745599-1 | 0.041 | 0.044 | 0.355 |  | -0.001 | 0.045 | 0.986 |  | 0.017 | 0.044 | 0.707 |
| rs2741856-2 | 0.125 | 0.230 | 0.586 |  | 0.055 | 0.231 | 0.810 |  | -0.192 | 0.229 | 0.402 |
| rs2741856-1 | 0.195 | 0.233 | 0.403 |  | 0.136 | 0.234 | 0.562 |  | -0.062 | 0.232 | 0.789 |
| rs2737252-2 | 0.019 | 0.067 | 0.774 |  | 0.008 | 0.067 | 0.911 |  | 0.045 | 0.067 | 0.499 |
| rs2737252-1 | 0.053 | 0.068 | 0.432 |  | 0.087 | 0.068 | 0.203 |  | 0.036 | 0.068 | 0.599 |
| rs2737207-2 | -0.067 | 0.050 | 0.185 |  | -0.064 | 0.051 | 0.203 |  | -0.001 | 0.050 | 0.986 |
| rs2737207-1 | -0.007 | 0.047 | 0.891 |  | 0.006 | 0.048 | 0.900 |  | 0.035 | 0.047 | 0.464 |
| rs273594-2 | -0.018 | 0.058 | 0.753 |  | -0.011 | 0.058 | 0.845 |  | 0.025 | 0.058 | 0.669 |
| rs273594-1 | -0.025 | 0.058 | 0.670 |  | 0.016 | 0.058 | 0.778 |  | -0.010 | 0.057 | 0.866 |
| rs2722176-2 | -0.059 | 0.056 | 0.295 |  | -0.053 | 0.057 | 0.351 |  | -0.045 | 0.056 | 0.418 |
| rs2722176-1 | -0.005 | 0.036 | 0.883 |  | 0.000 | 0.036 | 0.993 |  | -0.022 | 0.036 | 0.542 |
| rs2707518-2 | 0.031 | 0.072 | 0.664 |  | 0.016 | 0.072 | 0.823 |  | 0.100 | 0.072 | 0.164 |
| rs2707518-1 | 0.000 | 0.058 | 0.994 |  | 0.020 | 0.059 | 0.736 |  | 0.035 | 0.058 | 0.548 |
| rs2653559-2 | -0.098 | 0.094 | 0.296 |  | -0.015 | 0.094 | 0.876 |  | -0.060 | 0.094 | 0.519 |
| rs2653559-1 | -0.060 | 0.038 | 0.115 |  | -0.069 | 0.038 | 0.069 |  | -0.059 | 0.038 | 0.122 |
| rs2647978-2 | -0.045 | 0.060 | 0.453 |  | -0.103 | 0.060 | 0.087 |  | -0.065 | 0.060 | 0.275 |
| rs2647978-1 | -0.040 | 0.037 | 0.273 |  | -0.034 | 0.037 | 0.358 |  | -0.015 | 0.037 | 0.678 |
| rs2647462-2 | -0.009 | 0.092 | 0.919 |  | 0.021 | 0.092 | 0.821 |  | 0.070 | 0.092 | 0.448 |
| rs2647462-1 | 0.055 | 0.094 | 0.560 |  | 0.077 | 0.094 | 0.413 |  | 0.110 | 0.094 | 0.242 |
| rs2639953-2 | -0.072 | 0.048 | 0.137 |  | -0.084 | 0.048 | 0.081 |  | 0.035 | 0.048 | 0.466 |
| rs2639953-1 | -0.082 | 0.042 | 0.051 |  | -0.060 | 0.042 | 0.151 |  | 0.006 | 0.042 | 0.878 |
| rs2637317-2 | -0.026 | 0.197 | 0.896 |  | -0.026 | 0.198 | 0.894 |  | 0.041 | 0.196 | 0.836 |
| rs2637317-1 | -0.038 | 0.048 | 0.423 |  | -0.054 | 0.048 | 0.257 |  | -0.034 | 0.048 | 0.475 |
| rs2624834-2 | -0.059 | 0.102 | 0.567 |  | 0.013 | 0.103 | 0.902 |  | -0.010 | 0.102 | 0.922 |
| rs2624834-1 | -0.039 | 0.105 | 0.709 |  | 0.040 | 0.106 | 0.708 |  | 0.003 | 0.105 | 0.975 |
| rs2609352-2 | -0.132 | 0.060 | 0.027 |  | -0.088 | 0.060 | 0.143 |  | -0.046 | 0.059 | 0.440 |
| rs2609352-1 | -0.030 | 0.036 | 0.403 |  | 0.004 | 0.036 | 0.907 |  | 0.030 | 0.036 | 0.412 |
| rs2586457-2 | -0.046 | 0.082 | 0.572 |  | -0.017 | 0.082 | 0.836 |  | -0.069 | 0.082 | 0.399 |
| rs2586457-1 | -0.032 | 0.083 | 0.697 |  | -0.048 | 0.084 | 0.569 |  | -0.049 | 0.083 | 0.558 |
| rs2566774-2 | -0.060 | 0.089 | 0.503 |  | -0.073 | 0.089 | 0.416 |  | -0.043 | 0.089 | 0.632 |
| rs2566774-1 | -0.075 | 0.091 | 0.408 |  | -0.052 | 0.091 | 0.568 |  | -0.044 | 0.091 | 0.626 |
| rs2566752-2 | -0.100 | 0.055 | 0.071 |  | -0.097 | 0.055 | 0.080 |  | -0.198 | 0.055 | 0.000 |
| rs2566752-1 | -0.076 | 0.051 | 0.135 |  | -0.057 | 0.051 | 0.262 |  | -0.085 | 0.051 | 0.095 |
| rs2553772-2 | 0.005 | 0.050 | 0.917 |  | -0.006 | 0.050 | 0.903 |  | -0.049 | 0.049 | 0.318 |
| rs2553772-1 | -0.035 | 0.039 | 0.370 |  | -0.032 | 0.039 | 0.418 |  | -0.050 | 0.039 | 0.195 |
| rs2551769-2 | 0.021 | 0.062 | 0.735 |  | -0.052 | 0.062 | 0.405 |  | 0.017 | 0.062 | 0.787 |
| rs2551769-1 | -0.008 | 0.063 | 0.895 |  | -0.046 | 0.063 | 0.469 |  | -0.020 | 0.063 | 0.753 |
| rs2549722-2 | 0.079 | 0.195 | 0.686 |  | 0.079 | 0.196 | 0.687 |  | -0.283 | 0.195 | 0.147 |
| rs2549722-1 | -0.040 | 0.049 | 0.417 |  | -0.051 | 0.049 | 0.302 |  | 0.055 | 0.049 | 0.264 |
| rs2546984-2 | 0.231 | 0.079 | 0.004 |  | 0.219 | 0.080 | 0.006 |  | 0.110 | 0.080 | 0.168 |
| rs2546984-1 | 0.216 | 0.082 | 0.009 |  | 0.210 | 0.083 | 0.011 |  | 0.117 | 0.082 | 0.155 |
| rs2531992-2 | -0.011 | 0.112 | 0.924 |  | 0.024 | 0.113 | 0.832 |  | -0.022 | 0.112 | 0.844 |
| rs2531992-1 | -0.032 | 0.038 | 0.411 |  | -0.015 | 0.039 | 0.695 |  | -0.005 | 0.038 | 0.892 |
| rs2530459-2 | -0.017 | 0.050 | 0.725 |  | 0.001 | 0.050 | 0.980 |  | 0.022 | 0.049 | 0.658 |
| rs2530459-1 | -0.025 | 0.047 | 0.598 |  | -0.005 | 0.047 | 0.918 |  | 0.036 | 0.047 | 0.447 |
| rs2530394-2 | -0.069 | 0.055 | 0.215 |  | -0.025 | 0.056 | 0.654 |  | -0.068 | 0.055 | 0.221 |
| rs2530394-1 | -0.004 | 0.037 | 0.913 |  | 0.014 | 0.037 | 0.708 |  | -0.039 | 0.037 | 0.289 |
| rs2504101-2 | 0.004 | 0.052 | 0.939 |  | -0.054 | 0.052 | 0.297 |  | 0.023 | 0.051 | 0.650 |
| rs2504101-1 | 0.011 | 0.049 | 0.829 |  | -0.028 | 0.050 | 0.574 |  | 0.046 | 0.049 | 0.347 |
| rs2504069-2 | -0.081 | 0.068 | 0.230 |  | -0.075 | 0.068 | 0.271 |  | -0.096 | 0.067 | 0.153 |
| rs2504069-1 | 0.007 | 0.037 | 0.854 |  | 0.021 | 0.037 | 0.561 |  | -0.055 | 0.037 | 0.130 |
| rs2491105-2 | 0.035 | 0.078 | 0.654 |  | 0.004 | 0.078 | 0.964 |  | 0.050 | 0.078 | 0.516 |
| rs2491105-1 | 0.059 | 0.080 | 0.460 |  | 0.040 | 0.080 | 0.616 |  | 0.058 | 0.080 | 0.464 |
| rs2478777-2 | 0.106 | 0.091 | 0.243 |  | 0.115 | 0.091 | 0.208 |  | 0.172 | 0.090 | 0.058 |
| rs2478777-1 | -0.041 | 0.038 | 0.275 |  | 0.000 | 0.038 | 0.997 |  | -0.021 | 0.038 | 0.574 |
| rs2471032-2 | 0.012 | 0.101 | 0.907 |  | -0.092 | 0.101 | 0.366 |  | 0.067 | 0.101 | 0.504 |
| rs2471032-1 | -0.109 | 0.102 | 0.285 |  | -0.207 | 0.103 | 0.044 |  | -0.006 | 0.102 | 0.953 |
| rs2442599-2 | 0.020 | 0.064 | 0.759 |  | 0.077 | 0.065 | 0.235 |  | -0.020 | 0.064 | 0.753 |
| rs2442599-1 | -0.004 | 0.036 | 0.904 |  | -0.006 | 0.037 | 0.876 |  | -0.061 | 0.036 | 0.092 |
| rs243866-2 | 0.009 | 0.073 | 0.906 |  | 0.005 | 0.073 | 0.950 |  | 0.007 | 0.072 | 0.926 |
| rs243866-1 | -0.007 | 0.075 | 0.923 |  | -0.023 | 0.075 | 0.760 |  | 0.027 | 0.075 | 0.721 |
| rs2432236-2 | 0.030 | 0.049 | 0.542 |  | 0.000 | 0.049 | 0.993 |  | 0.047 | 0.049 | 0.338 |
| rs2432236-1 | 0.055 | 0.044 | 0.205 |  | 0.054 | 0.044 | 0.216 |  | 0.047 | 0.044 | 0.285 |
| rs2430689-2 | 0.040 | 0.053 | 0.455 |  | 0.050 | 0.053 | 0.345 |  | 0.059 | 0.053 | 0.264 |
| rs2430689-1 | 0.030 | 0.037 | 0.420 |  | -0.001 | 0.037 | 0.989 |  | 0.025 | 0.037 | 0.497 |
| rs2423151-2 | 0.057 | 0.057 | 0.318 |  | 0.079 | 0.057 | 0.169 |  | -0.011 | 0.057 | 0.849 |
| rs2423151-1 | 0.057 | 0.057 | 0.317 |  | 0.070 | 0.057 | 0.219 |  | 0.010 | 0.057 | 0.856 |
| rs2406255-2 | 0.114 | 0.089 | 0.199 |  | 0.118 | 0.089 | 0.186 |  | -0.005 | 0.089 | 0.952 |
| rs2406255-1 | 0.004 | 0.039 | 0.916 |  | 0.003 | 0.039 | 0.935 |  | -0.039 | 0.038 | 0.306 |
| rs2388854-2 | -0.076 | 0.062 | 0.218 |  | -0.051 | 0.062 | 0.407 |  | -0.022 | 0.062 | 0.724 |
| rs2388854-1 | -0.011 | 0.037 | 0.768 |  | -0.011 | 0.037 | 0.759 |  | -0.023 | 0.037 | 0.537 |
| rs2385450-2 | -0.150 | 0.054 | 0.006 |  | -0.110 | 0.054 | 0.043 |  | -0.137 | 0.054 | 0.011 |
| rs2385450-1 | -0.023 | 0.037 | 0.531 |  | -0.017 | 0.037 | 0.651 |  | -0.037 | 0.037 | 0.318 |
| rs238073-2 | 0.023 | 0.054 | 0.672 |  | 0.043 | 0.055 | 0.435 |  | -0.030 | 0.054 | 0.586 |
| rs238073-1 | 0.015 | 0.054 | 0.783 |  | -0.012 | 0.054 | 0.832 |  | -0.014 | 0.054 | 0.801 |
| rs2376600-2 | -0.067 | 0.059 | 0.260 |  | -0.032 | 0.059 | 0.588 |  | -0.020 | 0.059 | 0.736 |
| rs2376600-1 | -0.003 | 0.036 | 0.935 |  | 0.042 | 0.036 | 0.242 |  | 0.000 | 0.036 | 0.993 |
| rs2375683-2 | 0.060 | 0.070 | 0.390 |  | 0.050 | 0.070 | 0.478 |  | 0.013 | 0.070 | 0.850 |
| rs2375683-1 | -0.033 | 0.037 | 0.365 |  | -0.044 | 0.037 | 0.237 |  | 0.005 | 0.037 | 0.888 |
| rs2354413-2 | 0.011 | 0.051 | 0.823 |  | 0.005 | 0.051 | 0.919 |  | 0.054 | 0.051 | 0.289 |
| rs2354413-1 | 0.027 | 0.048 | 0.572 |  | 0.017 | 0.048 | 0.723 |  | 0.054 | 0.048 | 0.256 |
| rs2324873-2 | 0.158 | 0.078 | 0.043 |  | 0.126 | 0.078 | 0.110 |  | 0.022 | 0.078 | 0.782 |
| rs2324873-1 | 0.014 | 0.039 | 0.721 |  | 0.001 | 0.039 | 0.987 |  | -0.039 | 0.039 | 0.313 |
| rs231793-2 | -0.069 | 0.261 | 0.792 |  | -0.071 | 0.262 | 0.787 |  | 0.026 | 0.260 | 0.921 |
| rs231793-1 | -0.006 | 0.053 | 0.917 |  | -0.018 | 0.054 | 0.740 |  | -0.016 | 0.053 | 0.758 |
| rs2305489-2 | 0.338 | 0.264 | 0.200 |  | 0.297 | 0.265 | 0.262 |  | 0.299 | 0.263 | 0.255 |
| rs2305489-1 | 0.389 | 0.263 | 0.139 |  | 0.396 | 0.264 | 0.134 |  | 0.383 | 0.262 | 0.145 |
| rs2303696-2 | 0.026 | 0.052 | 0.615 |  | 0.030 | 0.053 | 0.571 |  | -0.018 | 0.052 | 0.735 |
| rs2303696-1 | 0.024 | 0.051 | 0.637 |  | 0.037 | 0.051 | 0.473 |  | -0.005 | 0.051 | 0.929 |
| rs2296028-2 | 0.061 | 0.107 | 0.568 |  | 0.100 | 0.108 | 0.353 |  | 0.007 | 0.107 | 0.944 |
| rs2296028-1 | -0.041 | 0.040 | 0.300 |  | -0.026 | 0.040 | 0.518 |  | -0.011 | 0.040 | 0.779 |
| rs2294358-2 | -0.196 | 0.475 | 0.680 |  | 0.217 | 0.477 | 0.649 |  | -0.323 | 0.474 | 0.495 |
| rs2294358-1 | -0.191 | 0.477 | 0.689 |  | 0.225 | 0.479 | 0.639 |  | -0.267 | 0.476 | 0.576 |
| rs2287644-2 | -0.085 | 0.067 | 0.203 |  | -0.091 | 0.067 | 0.175 |  | -0.008 | 0.067 | 0.908 |
| rs2287644-1 | -0.037 | 0.036 | 0.298 |  | -0.006 | 0.036 | 0.877 |  | -0.007 | 0.036 | 0.837 |
| rs2272443-2 | 0.027 | 0.073 | 0.715 |  | -0.058 | 0.074 | 0.433 |  | 0.028 | 0.073 | 0.698 |
| rs2272443-1 | 0.039 | 0.037 | 0.290 |  | 0.004 | 0.037 | 0.918 |  | 0.031 | 0.037 | 0.403 |
| rs2272224-2 | -0.124 | 0.067 | 0.065 |  | -0.102 | 0.068 | 0.132 |  | -0.058 | 0.067 | 0.386 |
| rs2272224-1 | -0.075 | 0.065 | 0.251 |  | -0.034 | 0.065 | 0.605 |  | -0.030 | 0.065 | 0.639 |
| rs2271483-2 | -0.039 | 0.051 | 0.438 |  | -0.023 | 0.051 | 0.646 |  | -0.063 | 0.051 | 0.211 |
| rs2271483-1 | -0.030 | 0.048 | 0.527 |  | -0.056 | 0.048 | 0.239 |  | -0.063 | 0.047 | 0.181 |
| rs2271329-2 | -0.058 | 0.100 | 0.562 |  | -0.080 | 0.100 | 0.427 |  | -0.032 | 0.100 | 0.749 |
| rs2271329-1 | -0.044 | 0.102 | 0.666 |  | -0.034 | 0.103 | 0.738 |  | -0.083 | 0.103 | 0.418 |
| rs2246221-2 | -0.096 | 0.048 | 0.047 |  | -0.080 | 0.049 | 0.101 |  | -0.142 | 0.048 | 0.003 |
| rs2246221-1 | 0.004 | 0.042 | 0.921 |  | 0.002 | 0.042 | 0.962 |  | 0.000 | 0.041 | 0.994 |
| rs2239200-2 | -0.016 | 0.136 | 0.904 |  | 0.002 | 0.136 | 0.986 |  | -0.055 | 0.135 | 0.684 |
| rs2239200-1 | -0.031 | 0.042 | 0.463 |  | -0.014 | 0.042 | 0.732 |  | 0.002 | 0.042 | 0.965 |
| rs2238686-2 | -0.090 | 0.128 | 0.481 |  | -0.143 | 0.128 | 0.266 |  | 0.082 | 0.127 | 0.519 |
| rs2238686-1 | -0.024 | 0.042 | 0.573 |  | -0.022 | 0.042 | 0.593 |  | -0.053 | 0.042 | 0.202 |
| rs2236407-2 | 0.004 | 0.054 | 0.938 |  | 0.077 | 0.054 | 0.153 |  | 0.121 | 0.053 | 0.023 |
| rs2236407-1 | -0.068 | 0.038 | 0.071 |  | -0.053 | 0.038 | 0.159 |  | 0.032 | 0.037 | 0.395 |
| rs2235485-2 | 0.136 | 0.118 | 0.250 |  | 0.131 | 0.118 | 0.269 |  | 0.122 | 0.118 | 0.298 |
| rs2235485-1 | 0.059 | 0.040 | 0.140 |  | 0.060 | 0.040 | 0.138 |  | 0.016 | 0.040 | 0.697 |
| rs2234693-2 | -0.022 | 0.050 | 0.662 |  | -0.041 | 0.050 | 0.407 |  | -0.116 | 0.050 | 0.019 |
| rs2234693-1 | -0.001 | 0.045 | 0.987 |  | -0.020 | 0.045 | 0.653 |  | -0.084 | 0.044 | 0.060 |
| rs2229768-2 | 0.047 | 0.079 | 0.550 |  | 0.050 | 0.080 | 0.527 |  | -0.013 | 0.079 | 0.869 |
| rs2229768-1 | -0.017 | 0.037 | 0.645 |  | -0.020 | 0.037 | 0.596 |  | -0.057 | 0.037 | 0.122 |
| rs2227607-2 | -0.081 | 0.161 | 0.616 |  | -0.029 | 0.162 | 0.858 |  | -0.052 | 0.160 | 0.747 |
| rs2227607-1 | -0.011 | 0.043 | 0.791 |  | 0.004 | 0.043 | 0.930 |  | 0.051 | 0.043 | 0.236 |
| rs2223853-2 | 0.002 | 0.054 | 0.968 |  | 0.031 | 0.055 | 0.575 |  | 0.104 | 0.054 | 0.055 |
| rs2223853-1 | 0.044 | 0.053 | 0.402 |  | 0.072 | 0.053 | 0.173 |  | 0.118 | 0.053 | 0.024 |
| rs2218069-2 | -0.200 | 0.056 | 0.000 |  | -0.178 | 0.056 | 0.002 |  | -0.111 | 0.056 | 0.049 |
| rs2218069-1 | -0.156 | 0.056 | 0.006 |  | -0.144 | 0.057 | 0.011 |  | -0.076 | 0.056 | 0.176 |
| rs2216949-2 | -0.270 | 0.117 | 0.021 |  | -0.221 | 0.118 | 0.061 |  | -0.111 | 0.118 | 0.346 |
| rs2216949-1 | -0.170 | 0.120 | 0.157 |  | -0.138 | 0.121 | 0.252 |  | -0.038 | 0.120 | 0.755 |
| rs2204015-2 | 0.003 | 0.076 | 0.964 |  | -0.003 | 0.076 | 0.965 |  | 0.039 | 0.076 | 0.604 |
| rs2204015-1 | 0.019 | 0.078 | 0.811 |  | -0.017 | 0.079 | 0.831 |  | 0.016 | 0.078 | 0.837 |
| rs2174633-2 | 0.014 | 0.066 | 0.832 |  | 0.032 | 0.067 | 0.629 |  | 0.014 | 0.066 | 0.836 |
| rs2174633-1 | 0.071 | 0.036 | 0.049 |  | 0.108 | 0.036 | 0.003 |  | 0.038 | 0.036 | 0.287 |
| rs2160525-2 | -0.015 | 0.091 | 0.872 |  | 0.025 | 0.092 | 0.788 |  | -0.074 | 0.091 | 0.421 |
| rs2160525-1 | -0.012 | 0.095 | 0.899 |  | 0.023 | 0.095 | 0.812 |  | -0.078 | 0.095 | 0.414 |
| rs2155572-2 | -0.025 | 0.049 | 0.605 |  | -0.016 | 0.049 | 0.739 |  | -0.003 | 0.049 | 0.957 |
| rs2155572-1 | -0.023 | 0.047 | 0.623 |  | -0.001 | 0.047 | 0.990 |  | -0.018 | 0.047 | 0.696 |
| rs2153672-2 | -0.275 | 0.182 | 0.132 |  | -0.120 | 0.183 | 0.512 |  | 0.078 | 0.182 | 0.668 |
| rs2153672-1 | -0.259 | 0.185 | 0.161 |  | -0.120 | 0.186 | 0.520 |  | 0.074 | 0.184 | 0.688 |
| rs215226-2 | 0.061 | 0.051 | 0.232 |  | 0.044 | 0.051 | 0.387 |  | 0.031 | 0.051 | 0.546 |
| rs215226-1 | 0.094 | 0.048 | 0.048 |  | 0.059 | 0.048 | 0.216 |  | 0.060 | 0.048 | 0.207 |
| rs2135963-2 | 0.075 | 0.050 | 0.134 |  | 0.054 | 0.050 | 0.283 |  | 0.012 | 0.050 | 0.810 |
| rs2135963-1 | -0.015 | 0.039 | 0.695 |  | 0.011 | 0.039 | 0.779 |  | -0.003 | 0.039 | 0.930 |
| rs212772-2 | 0.005 | 0.050 | 0.927 |  | -0.046 | 0.050 | 0.362 |  | -0.099 | 0.050 | 0.047 |
| rs212772-1 | 0.036 | 0.045 | 0.427 |  | 0.004 | 0.046 | 0.935 |  | -0.063 | 0.045 | 0.165 |
| rs2126264-2 | -0.378 | 0.194 | 0.051 |  | -0.345 | 0.195 | 0.076 |  | -0.410 | 0.193 | 0.034 |
| rs2126264-1 | -0.409 | 0.196 | 0.037 |  | -0.357 | 0.197 | 0.070 |  | -0.398 | 0.195 | 0.042 |
| rs212417-2 | -0.053 | 0.055 | 0.330 |  | -0.090 | 0.055 | 0.102 |  | -0.107 | 0.055 | 0.050 |
| rs212417-1 | -0.033 | 0.054 | 0.539 |  | -0.004 | 0.054 | 0.935 |  | -0.092 | 0.054 | 0.090 |
| rs2107801-2 | 0.008 | 0.051 | 0.881 |  | 0.009 | 0.052 | 0.861 |  | -0.037 | 0.051 | 0.472 |
| rs2107801-1 | -0.045 | 0.049 | 0.360 |  | -0.015 | 0.049 | 0.754 |  | -0.024 | 0.049 | 0.628 |
| rs210374-2 | -0.043 | 0.064 | 0.502 |  | -0.049 | 0.064 | 0.446 |  | 0.082 | 0.063 | 0.196 |
| rs210374-1 | -0.075 | 0.036 | 0.038 |  | -0.073 | 0.036 | 0.044 |  | -0.034 | 0.036 | 0.348 |
| rs2097719-2 | -0.008 | 0.053 | 0.884 |  | -0.009 | 0.053 | 0.868 |  | 0.007 | 0.053 | 0.892 |
| rs2097719-1 | 0.028 | 0.051 | 0.578 |  | 0.036 | 0.051 | 0.473 |  | 0.092 | 0.051 | 0.068 |
| rs2095931-2 | 0.030 | 0.122 | 0.807 |  | -0.025 | 0.122 | 0.836 |  | 0.174 | 0.121 | 0.152 |
| rs2095931-1 | 0.035 | 0.040 | 0.380 |  | 0.065 | 0.040 | 0.105 |  | -0.018 | 0.040 | 0.648 |
| rs2091624-2 | 0.076 | 0.114 | 0.504 |  | 0.024 | 0.115 | 0.831 |  | 0.026 | 0.114 | 0.819 |
| rs2091624-1 | -0.086 | 0.043 | 0.047 |  | -0.065 | 0.043 | 0.137 |  | -0.033 | 0.043 | 0.451 |
| rs2069442-2 | -0.138 | 0.065 | 0.033 |  | -0.077 | 0.065 | 0.238 |  | -0.025 | 0.065 | 0.701 |
| rs2069442-1 | 0.011 | 0.036 | 0.768 |  | 0.019 | 0.036 | 0.598 |  | 0.018 | 0.036 | 0.621 |
| rs2052480-2 | -0.002 | 0.066 | 0.974 |  | -0.018 | 0.066 | 0.781 |  | -0.042 | 0.066 | 0.524 |
| rs2052480-1 | 0.034 | 0.068 | 0.619 |  | 0.000 | 0.068 | 0.997 |  | 0.012 | 0.068 | 0.864 |
| rs2038726-2 | -0.186 | 0.263 | 0.479 |  | -0.151 | 0.264 | 0.566 |  | -0.063 | 0.262 | 0.811 |
| rs2038726-1 | -0.114 | 0.266 | 0.667 |  | -0.124 | 0.267 | 0.641 |  | -0.026 | 0.265 | 0.920 |
| rs2038158-2 | 0.023 | 0.049 | 0.634 |  | 0.018 | 0.050 | 0.710 |  | 0.009 | 0.049 | 0.851 |
| rs2038158-1 | 0.089 | 0.043 | 0.038 |  | 0.106 | 0.043 | 0.013 |  | 0.029 | 0.043 | 0.497 |
| rs200662204-2 | -1.823 | 0.690 | 0.008 |  | -2.314 | 0.693 | 0.001 |  | -0.920 | 0.688 | 0.181 |
| rs199281-2 | -0.041 | 0.104 | 0.690 |  | -0.067 | 0.104 | 0.521 |  | -0.121 | 0.103 | 0.244 |
| rs199281-1 | 0.034 | 0.039 | 0.388 |  | 0.004 | 0.039 | 0.917 |  | 0.019 | 0.039 | 0.623 |
| rs1991431-2 | 0.010 | 0.050 | 0.848 |  | 0.033 | 0.051 | 0.513 |  | 0.087 | 0.050 | 0.084 |
| rs1991431-1 | -0.020 | 0.038 | 0.602 |  | -0.003 | 0.038 | 0.929 |  | 0.014 | 0.038 | 0.711 |
| rs1983853-2 | -0.030 | 0.136 | 0.825 |  | 0.060 | 0.137 | 0.660 |  | -0.275 | 0.136 | 0.043 |
| rs1983853-1 | 0.005 | 0.140 | 0.971 |  | 0.089 | 0.141 | 0.527 |  | -0.276 | 0.140 | 0.049 |
| rs194608-2 | -0.093 | 0.416 | 0.822 |  | -0.236 | 0.418 | 0.573 |  | -0.492 | 0.415 | 0.236 |
| rs194608-1 | 0.078 | 0.422 | 0.853 |  | -0.070 | 0.424 | 0.870 |  | -0.338 | 0.420 | 0.422 |
| rs191927852-2 | 0.500 | 0.684 | 0.465 |  | 0.775 | 0.687 | 0.259 |  | 1.342 | 0.681 | 0.049 |
| rs191927852-1 | 0.112 | 0.104 | 0.280 |  | 0.091 | 0.104 | 0.380 |  | 0.214 | 0.103 | 0.038 |
| rs1907310-2 | -0.073 | 0.088 | 0.405 |  | 0.004 | 0.089 | 0.962 |  | -0.036 | 0.088 | 0.678 |
| rs1907310-1 | -0.076 | 0.090 | 0.398 |  | -0.003 | 0.090 | 0.978 |  | 0.011 | 0.090 | 0.899 |
| rs1904398-2 | 0.041 | 0.048 | 0.390 |  | 0.052 | 0.048 | 0.274 |  | 0.035 | 0.047 | 0.467 |
| rs1904398-1 | 0.004 | 0.041 | 0.918 |  | 0.005 | 0.041 | 0.909 |  | 0.041 | 0.041 | 0.316 |
| rs1902177-2 | -0.071 | 0.050 | 0.161 |  | -0.034 | 0.051 | 0.496 |  | -0.018 | 0.050 | 0.714 |
| rs1902177-1 | 0.001 | 0.039 | 0.985 |  | 0.009 | 0.040 | 0.828 |  | 0.022 | 0.039 | 0.569 |
| rs190144-2 | -0.065 | 0.069 | 0.348 |  | -0.121 | 0.069 | 0.080 |  | 0.029 | 0.069 | 0.678 |
| rs190144-1 | 0.021 | 0.070 | 0.763 |  | -0.014 | 0.070 | 0.842 |  | 0.066 | 0.070 | 0.345 |
| rs1897468-2 | 0.012 | 0.063 | 0.851 |  | -0.022 | 0.063 | 0.723 |  | -0.060 | 0.063 | 0.341 |
| rs1897468-1 | 0.029 | 0.064 | 0.654 |  | -0.010 | 0.065 | 0.879 |  | -0.073 | 0.064 | 0.254 |
| rs189448695-2 | -0.399 | 0.243 | 0.101 |  | -0.265 | 0.245 | 0.278 |  | -0.397 | 0.243 | 0.102 |
| rs1883282-2 | -0.031 | 0.086 | 0.719 |  | -0.046 | 0.087 | 0.595 |  | 0.078 | 0.086 | 0.362 |
| rs1883282-1 | -0.016 | 0.037 | 0.661 |  | -0.025 | 0.038 | 0.504 |  | -0.004 | 0.037 | 0.909 |
| rs188137993-2 | 0.113 | 0.231 | 0.624 |  | 0.134 | 0.232 | 0.562 |  | 0.030 | 0.230 | 0.897 |
| rs1880842-2 | 0.167 | 0.065 | 0.010 |  | 0.118 | 0.065 | 0.070 |  | 0.104 | 0.065 | 0.108 |
| rs1880842-1 | 0.141 | 0.065 | 0.031 |  | 0.099 | 0.066 | 0.134 |  | 0.063 | 0.065 | 0.337 |
| rs1878526-2 | -0.108 | 0.085 | 0.204 |  | -0.140 | 0.085 | 0.099 |  | -0.182 | 0.084 | 0.031 |
| rs1878526-1 | -0.093 | 0.084 | 0.269 |  | -0.154 | 0.084 | 0.068 |  | -0.162 | 0.084 | 0.053 |
| rs1871859-2 | 0.245 | 0.116 | 0.035 |  | 0.241 | 0.117 | 0.039 |  | -0.092 | 0.116 | 0.427 |
| rs1871859-1 | -0.010 | 0.046 | 0.833 |  | 0.015 | 0.047 | 0.755 |  | -0.070 | 0.046 | 0.132 |
| rs1871674-2 | -0.101 | 0.086 | 0.242 |  | -0.078 | 0.086 | 0.368 |  | -0.113 | 0.086 | 0.186 |
| rs1871674-1 | -0.127 | 0.089 | 0.152 |  | -0.101 | 0.089 | 0.256 |  | -0.060 | 0.088 | 0.499 |
| rs186692714-2 | 3.394 | 1.426 | 0.017 |  | 2.351 | 1.433 | 0.101 |  | 2.266 | 1.421 | 0.111 |
| rs186692714-1 | -0.061 | 0.124 | 0.622 |  | -0.035 | 0.125 | 0.777 |  | 0.060 | 0.124 | 0.627 |
| rs1844776-2 | 0.013 | 0.050 | 0.795 |  | -0.029 | 0.050 | 0.567 |  | 0.089 | 0.050 | 0.076 |
| rs1844776-1 | -0.010 | 0.038 | 0.786 |  | -0.045 | 0.038 | 0.244 |  | 0.042 | 0.038 | 0.265 |
| rs184081583-2 | -0.074 | 0.230 | 0.748 |  | -0.094 | 0.231 | 0.684 |  | -0.323 | 0.229 | 0.160 |
| rs183522757-2 | 0.304 | 0.288 | 0.292 |  | 0.360 | 0.289 | 0.213 |  | -0.177 | 0.295 | 0.548 |
| rs183522757-1 | -0.068 | 0.052 | 0.196 |  | -0.062 | 0.053 | 0.240 |  | -0.061 | 0.052 | 0.243 |
| rs183424609-1 | -0.728 | 0.283 | 0.010 |  | -0.648 | 0.284 | 0.023 |  | -0.475 | 0.282 | 0.092 |
| rs183312687-2 | 0.136 | 0.191 | 0.475 |  | 0.198 | 0.192 | 0.302 |  | 0.363 | 0.190 | 0.057 |
| rs182975328-2 | -0.176 | 0.139 | 0.204 |  | -0.273 | 0.139 | 0.050 |  | -0.235 | 0.138 | 0.089 |
| rs182103652-1 | -0.203 | 0.286 | 0.478 |  | -0.095 | 0.287 | 0.740 |  | -0.348 | 0.285 | 0.222 |
| rs181832503-1 | 0.052 | 0.312 | 0.868 |  | -0.104 | 0.313 | 0.741 |  | 0.074 | 0.311 | 0.813 |
| rs181043390-2 | -0.081 | 0.152 | 0.595 |  | 0.035 | 0.153 | 0.820 |  | -0.106 | 0.152 | 0.487 |
| rs180077-2 | 0.095 | 0.059 | 0.105 |  | 0.163 | 0.059 | 0.006 |  | -0.014 | 0.059 | 0.816 |
| rs180077-1 | 0.006 | 0.037 | 0.861 |  | 0.035 | 0.037 | 0.342 |  | -0.037 | 0.036 | 0.305 |
| rs1779431-2 | -0.034 | 0.061 | 0.579 |  | 0.004 | 0.062 | 0.947 |  | 0.162 | 0.061 | 0.008 |
| rs1779431-1 | -0.097 | 0.062 | 0.119 |  | -0.052 | 0.062 | 0.405 |  | 0.077 | 0.062 | 0.214 |
| rs17773169-2 | 0.063 | 0.054 | 0.241 |  | 0.044 | 0.054 | 0.412 |  | -0.003 | 0.054 | 0.960 |
| rs17773169-1 | 0.038 | 0.038 | 0.314 |  | 0.061 | 0.038 | 0.106 |  | 0.052 | 0.037 | 0.164 |
| rs1777277-2 | 0.008 | 0.048 | 0.868 |  | -0.005 | 0.048 | 0.912 |  | -0.029 | 0.048 | 0.543 |
| rs1777277-1 | -0.028 | 0.039 | 0.473 |  | -0.001 | 0.039 | 0.983 |  | -0.043 | 0.039 | 0.277 |
| rs17733713-2 | -0.029 | 0.164 | 0.861 |  | 0.041 | 0.165 | 0.804 |  | -0.101 | 0.163 | 0.537 |
| rs17733713-1 | 0.028 | 0.169 | 0.869 |  | 0.081 | 0.169 | 0.632 |  | -0.060 | 0.168 | 0.719 |
| rs1767447-2 | 0.264 | 0.328 | 0.420 |  | -0.026 | 0.329 | 0.936 |  | 0.644 | 0.337 | 0.056 |
| rs1767447-1 | 0.373 | 0.331 | 0.260 |  | 0.088 | 0.333 | 0.792 |  | 0.664 | 0.341 | 0.051 |
| rs17662822-2 | 0.057 | 0.063 | 0.360 |  | 0.061 | 0.063 | 0.334 |  | -0.132 | 0.063 | 0.035 |
| rs17662822-1 | 0.000 | 0.039 | 0.991 |  | -0.010 | 0.039 | 0.794 |  | -0.041 | 0.039 | 0.292 |
| rs17650301-2 | 0.000 | 0.060 | 0.995 |  | -0.020 | 0.060 | 0.738 |  | -0.045 | 0.059 | 0.445 |
| rs17650301-1 | 0.003 | 0.036 | 0.939 |  | -0.009 | 0.036 | 0.806 |  | -0.024 | 0.036 | 0.510 |
| rs17612444-2 | -0.181 | 0.254 | 0.475 |  | -0.102 | 0.255 | 0.690 |  | -0.115 | 0.258 | 0.656 |
| rs17612444-1 | -0.235 | 0.259 | 0.363 |  | -0.131 | 0.260 | 0.616 |  | -0.108 | 0.263 | 0.683 |
| rs17602572-2 | 0.020 | 0.052 | 0.700 |  | 0.035 | 0.053 | 0.505 |  | -0.015 | 0.052 | 0.780 |
| rs17602572-1 | 0.014 | 0.037 | 0.708 |  | -0.001 | 0.037 | 0.974 |  | 0.021 | 0.037 | 0.569 |
| rs17598579-2 | -0.110 | 0.133 | 0.408 |  | -0.185 | 0.134 | 0.167 |  | 0.043 | 0.133 | 0.747 |
| rs17598579-1 | -0.023 | 0.041 | 0.584 |  | -0.003 | 0.041 | 0.949 |  | -0.016 | 0.041 | 0.692 |
| rs17563-2 | -0.090 | 0.063 | 0.154 |  | -0.105 | 0.063 | 0.097 |  | -0.151 | 0.063 | 0.017 |
| rs17563-1 | -0.052 | 0.050 | 0.296 |  | -0.070 | 0.050 | 0.165 |  | -0.130 | 0.050 | 0.009 |
| rs17537246-2 | -0.061 | 0.377 | 0.872 |  | -0.592 | 0.378 | 0.118 |  | -0.178 | 0.375 | 0.636 |
| rs17537246-1 | -0.042 | 0.380 | 0.913 |  | -0.602 | 0.382 | 0.115 |  | -0.094 | 0.379 | 0.804 |
| rs17514738-2 | 0.061 | 0.050 | 0.229 |  | 0.074 | 0.051 | 0.144 |  | 0.043 | 0.050 | 0.393 |
| rs17514738-1 | -0.065 | 0.037 | 0.081 |  | -0.054 | 0.038 | 0.148 |  | -0.024 | 0.037 | 0.513 |
| rs17510563-2 | -0.223 | 0.181 | 0.218 |  | -0.102 | 0.182 | 0.574 |  | -0.077 | 0.181 | 0.669 |
| rs17510563-1 | -0.122 | 0.185 | 0.510 |  | -0.031 | 0.186 | 0.868 |  | -0.002 | 0.184 | 0.991 |
| rs17507577-2 | -0.088 | 0.241 | 0.715 |  | 0.041 | 0.242 | 0.867 |  | -0.096 | 0.240 | 0.690 |
| rs17507577-1 | -0.015 | 0.244 | 0.951 |  | 0.113 | 0.245 | 0.645 |  | -0.075 | 0.243 | 0.757 |
| rs17501090-2 | 0.353 | 1.243 | 0.776 |  | -0.369 | 1.249 | 0.768 |  | 0.032 | 1.240 | 0.980 |
| rs17501090-1 | 0.080 | 0.088 | 0.360 |  | 0.054 | 0.088 | 0.538 |  | 0.114 | 0.087 | 0.191 |
| rs17498550-2 | 0.043 | 0.076 | 0.574 |  | 0.012 | 0.076 | 0.878 |  | 0.017 | 0.076 | 0.821 |
| rs17498550-1 | 0.042 | 0.036 | 0.242 |  | -0.002 | 0.036 | 0.959 |  | 0.072 | 0.036 | 0.046 |
| rs17479840-1 | -0.157 | 0.113 | 0.163 |  | -0.086 | 0.113 | 0.449 |  | 0.156 | 0.112 | 0.165 |
| rs174534-2 | -0.113 | 0.055 | 0.041 |  | -0.098 | 0.056 | 0.079 |  | -0.032 | 0.055 | 0.563 |
| rs174534-1 | -0.096 | 0.055 | 0.084 |  | -0.093 | 0.056 | 0.095 |  | 0.031 | 0.055 | 0.573 |
| rs17429745-2 | 0.160 | 0.059 | 0.006 |  | 0.163 | 0.059 | 0.006 |  | 0.117 | 0.058 | 0.046 |
| rs17429745-1 | 0.109 | 0.056 | 0.052 |  | 0.118 | 0.056 | 0.036 |  | 0.076 | 0.056 | 0.175 |
| rs1736213-2 | -0.108 | 0.049 | 0.027 |  | -0.082 | 0.049 | 0.095 |  | -0.063 | 0.049 | 0.198 |
| rs1736213-1 | -0.063 | 0.046 | 0.168 |  | -0.026 | 0.046 | 0.578 |  | -0.024 | 0.046 | 0.601 |
| rs17351518-2 | 0.036 | 0.058 | 0.528 |  | 0.024 | 0.058 | 0.676 |  | -0.030 | 0.057 | 0.598 |
| rs17351518-1 | 0.071 | 0.057 | 0.216 |  | 0.070 | 0.057 | 0.226 |  | 0.060 | 0.057 | 0.296 |
| rs17307280-2 | -0.118 | 0.038 | 0.002 |  | -0.100 | 0.038 | 0.009 |  | -0.177 | 0.038 | 0.000 |
| rs17302855-1 | 0.010 | 0.035 | 0.773 |  | 0.001 | 0.035 | 0.978 |  | 0.027 | 0.035 | 0.435 |
| rs17288588-2 | -0.014 | 0.085 | 0.870 |  | 0.036 | 0.085 | 0.669 |  | -0.026 | 0.085 | 0.757 |
| rs17288588-1 | 0.039 | 0.087 | 0.652 |  | 0.064 | 0.087 | 0.466 |  | 0.036 | 0.087 | 0.675 |
| rs17278798-2 | -0.101 | 0.054 | 0.061 |  | -0.095 | 0.054 | 0.081 |  | 0.016 | 0.054 | 0.774 |
| rs17278798-1 | -0.157 | 0.053 | 0.003 |  | -0.149 | 0.053 | 0.005 |  | -0.090 | 0.053 | 0.088 |
| rs17265513-2 | 0.189 | 0.094 | 0.045 |  | 0.143 | 0.095 | 0.132 |  | 0.083 | 0.094 | 0.375 |
| rs17265513-1 | 0.023 | 0.039 | 0.546 |  | -0.004 | 0.039 | 0.924 |  | -0.027 | 0.039 | 0.484 |
| rs17249128-2 | 0.008 | 0.048 | 0.870 |  | 0.031 | 0.048 | 0.523 |  | 0.061 | 0.048 | 0.207 |
| rs17249128-1 | -0.007 | 0.042 | 0.868 |  | 0.009 | 0.042 | 0.826 |  | 0.029 | 0.041 | 0.482 |
| rs1724298-2 | -0.175 | 0.215 | 0.414 |  | -0.067 | 0.216 | 0.756 |  | -0.157 | 0.214 | 0.463 |
| rs1724298-1 | -0.154 | 0.217 | 0.478 |  | -0.098 | 0.218 | 0.654 |  | -0.196 | 0.216 | 0.364 |
| rs17184382-2 | -0.027 | 0.050 | 0.590 |  | -0.021 | 0.050 | 0.678 |  | -0.011 | 0.050 | 0.822 |
| rs17184382-1 | -0.033 | 0.038 | 0.390 |  | -0.041 | 0.038 | 0.287 |  | -0.054 | 0.038 | 0.153 |
| rs171835-2 | 0.130 | 0.071 | 0.067 |  | 0.098 | 0.071 | 0.167 |  | 0.128 | 0.070 | 0.068 |
| rs171835-1 | 0.147 | 0.072 | 0.042 |  | 0.080 | 0.072 | 0.268 |  | 0.160 | 0.072 | 0.026 |
| rs1717720-2 | 0.028 | 0.058 | 0.622 |  | 0.006 | 0.058 | 0.921 |  | -0.090 | 0.058 | 0.120 |
| rs1717720-1 | -0.046 | 0.038 | 0.230 |  | -0.070 | 0.038 | 0.067 |  | -0.080 | 0.038 | 0.033 |
| rs17173698-2 | 1.228 | 1.205 | 0.308 |  | 0.191 | 1.210 | 0.874 |  | -0.753 | 1.201 | 0.531 |
| rs17173698-1 | 1.405 | 1.207 | 0.244 |  | 0.310 | 1.213 | 0.798 |  | -0.633 | 1.203 | 0.599 |
| rs17166182-2 | 0.052 | 0.054 | 0.338 |  | 0.019 | 0.055 | 0.725 |  | 0.032 | 0.054 | 0.558 |
| rs17166182-1 | -0.026 | 0.038 | 0.484 |  | -0.025 | 0.038 | 0.516 |  | -0.045 | 0.038 | 0.229 |
| rs17115088-2 | 0.227 | 0.436 | 0.603 |  | 0.231 | 0.438 | 0.598 |  | 0.025 | 0.435 | 0.953 |
| rs17115088-1 | 0.023 | 0.081 | 0.774 |  | 0.024 | 0.082 | 0.766 |  | -0.037 | 0.081 | 0.647 |
| rs1706708-2 | -0.004 | 0.065 | 0.954 |  | -0.020 | 0.065 | 0.755 |  | -0.006 | 0.065 | 0.931 |
| rs1706708-1 | -0.020 | 0.036 | 0.578 |  | -0.018 | 0.036 | 0.621 |  | -0.018 | 0.036 | 0.616 |
| rs17061975-2 | 0.286 | 0.233 | 0.220 |  | 0.187 | 0.234 | 0.424 |  | 0.200 | 0.232 | 0.389 |
| rs17061975-1 | 0.287 | 0.237 | 0.226 |  | 0.164 | 0.238 | 0.492 |  | 0.156 | 0.236 | 0.510 |
| rs17035323-2 | -0.164 | 0.114 | 0.153 |  | -0.257 | 0.115 | 0.025 |  | -0.205 | 0.114 | 0.072 |
| rs17035323-1 | -0.026 | 0.041 | 0.522 |  | -0.021 | 0.041 | 0.604 |  | -0.008 | 0.041 | 0.836 |
| rs1703492-2 | 0.033 | 0.059 | 0.577 |  | 0.046 | 0.059 | 0.435 |  | -0.003 | 0.059 | 0.955 |
| rs1703492-1 | -0.003 | 0.036 | 0.934 |  | -0.004 | 0.036 | 0.911 |  | -0.013 | 0.036 | 0.723 |
| rs17034046-2 | 0.021 | 0.090 | 0.820 |  | 0.098 | 0.091 | 0.282 |  | 0.113 | 0.090 | 0.211 |
| rs17034046-1 | 0.057 | 0.039 | 0.145 |  | 0.067 | 0.039 | 0.086 |  | 0.029 | 0.039 | 0.460 |
| rs17033059-2 | -0.043 | 0.098 | 0.661 |  | -0.110 | 0.098 | 0.265 |  | -0.105 | 0.097 | 0.281 |
| rs17033059-1 | -0.062 | 0.038 | 0.100 |  | -0.053 | 0.038 | 0.163 |  | -0.054 | 0.038 | 0.154 |
| rs17030818-2 | -0.089 | 0.159 | 0.574 |  | -0.267 | 0.159 | 0.094 |  | 0.008 | 0.158 | 0.961 |
| rs17030818-1 | -0.077 | 0.162 | 0.634 |  | -0.272 | 0.163 | 0.096 |  | 0.035 | 0.162 | 0.830 |
| rs17013212-2 | -0.142 | 0.106 | 0.182 |  | -0.051 | 0.107 | 0.633 |  | -0.084 | 0.106 | 0.428 |
| rs17013212-1 | 0.045 | 0.041 | 0.280 |  | -0.004 | 0.041 | 0.919 |  | -0.016 | 0.041 | 0.691 |
| rs17010957-2 | 0.132 | 0.114 | 0.246 |  | 0.163 | 0.114 | 0.155 |  | 0.127 | 0.114 | 0.264 |
| rs17010957-1 | 0.138 | 0.117 | 0.238 |  | 0.150 | 0.117 | 0.201 |  | 0.090 | 0.116 | 0.440 |
| rs16976954-2 | -0.026 | 0.054 | 0.631 |  | 0.004 | 0.054 | 0.945 |  | -0.041 | 0.054 | 0.446 |
| rs16976954-1 | -0.033 | 0.041 | 0.417 |  | -0.039 | 0.041 | 0.345 |  | -0.051 | 0.041 | 0.207 |
| rs16948744-2 | -0.012 | 0.055 | 0.822 |  | -0.023 | 0.055 | 0.670 |  | 0.050 | 0.055 | 0.362 |
| rs16948744-1 | -0.029 | 0.037 | 0.438 |  | -0.034 | 0.038 | 0.367 |  | 0.009 | 0.037 | 0.800 |
| rs16908520-2 | 0.033 | 0.093 | 0.725 |  | -0.097 | 0.093 | 0.297 |  | -0.052 | 0.093 | 0.572 |
| rs16908520-1 | 0.035 | 0.038 | 0.356 |  | -0.013 | 0.038 | 0.741 |  | 0.056 | 0.038 | 0.137 |
| rs1689583-2 | 0.004 | 0.107 | 0.970 |  | 0.005 | 0.107 | 0.962 |  | -0.018 | 0.107 | 0.864 |
| rs1689583-1 | 0.011 | 0.038 | 0.774 |  | -0.024 | 0.038 | 0.521 |  | -0.019 | 0.038 | 0.613 |
| rs16878921-2 | -0.090 | 0.141 | 0.524 |  | -0.084 | 0.141 | 0.554 |  | -0.109 | 0.140 | 0.438 |
| rs16878921-1 | -0.004 | 0.143 | 0.980 |  | -0.026 | 0.144 | 0.858 |  | -0.031 | 0.143 | 0.831 |
| rs16838527-2 | -0.375 | 0.188 | 0.046 |  | -0.307 | 0.189 | 0.104 |  | -0.331 | 0.187 | 0.078 |
| rs16838527-1 | -0.039 | 0.046 | 0.394 |  | -0.043 | 0.046 | 0.353 |  | -0.055 | 0.046 | 0.231 |
| rs167365-2 | -0.050 | 0.053 | 0.342 |  | -0.096 | 0.053 | 0.070 |  | 0.044 | 0.053 | 0.402 |
| rs167365-1 | -0.007 | 0.052 | 0.893 |  | -0.020 | 0.052 | 0.699 |  | 0.078 | 0.052 | 0.133 |
| rs167024-2 | 0.016 | 0.049 | 0.749 |  | 0.001 | 0.049 | 0.980 |  | 0.003 | 0.049 | 0.944 |
| rs167024-1 | 0.005 | 0.039 | 0.902 |  | 0.003 | 0.039 | 0.938 |  | -0.014 | 0.039 | 0.720 |
| rs1667747-2 | 0.042 | 0.079 | 0.592 |  | 0.045 | 0.080 | 0.572 |  | -0.025 | 0.079 | 0.749 |
| rs1667747-1 | 0.082 | 0.082 | 0.313 |  | 0.094 | 0.082 | 0.252 |  | -0.010 | 0.081 | 0.901 |
| rs1661725-2 | -0.100 | 0.053 | 0.057 |  | -0.066 | 0.053 | 0.211 |  | -0.015 | 0.053 | 0.779 |
| rs1661725-1 | -0.109 | 0.050 | 0.030 |  | -0.097 | 0.051 | 0.055 |  | -0.064 | 0.050 | 0.201 |
| rs1658469-2 | 0.106 | 0.048 | 0.029 |  | 0.058 | 0.049 | 0.229 |  | 0.032 | 0.048 | 0.508 |
| rs1658469-1 | 0.019 | 0.042 | 0.651 |  | 0.001 | 0.043 | 0.975 |  | -0.001 | 0.042 | 0.981 |
| rs163433-2 | 0.083 | 0.049 | 0.093 |  | 0.095 | 0.049 | 0.053 |  | 0.048 | 0.049 | 0.331 |
| rs163433-1 | 0.017 | 0.041 | 0.686 |  | 0.040 | 0.042 | 0.335 |  | 0.029 | 0.041 | 0.487 |
| rs1622638-2 | 0.000 | 0.052 | 1.000 |  | 0.054 | 0.052 | 0.297 |  | 0.039 | 0.051 | 0.446 |
| rs1622638-1 | 0.015 | 0.049 | 0.758 |  | 0.044 | 0.049 | 0.374 |  | 0.057 | 0.049 | 0.245 |
| rs1609829-2 | -0.041 | 0.060 | 0.494 |  | -0.087 | 0.060 | 0.147 |  | -0.005 | 0.059 | 0.933 |
| rs1609829-1 | 0.002 | 0.060 | 0.975 |  | -0.033 | 0.060 | 0.587 |  | 0.021 | 0.060 | 0.725 |
| rs1581630-2 | -0.251 | 0.070 | 0.000 |  | -0.214 | 0.070 | 0.002 |  | -0.036 | 0.069 | 0.605 |
| rs1581630-1 | -0.115 | 0.071 | 0.106 |  | -0.085 | 0.071 | 0.235 |  | 0.045 | 0.071 | 0.524 |
| rs1575667-2 | -0.147 | 0.143 | 0.304 |  | -0.151 | 0.144 | 0.295 |  | -0.165 | 0.143 | 0.249 |
| rs1575667-1 | -0.140 | 0.146 | 0.340 |  | -0.138 | 0.147 | 0.348 |  | -0.144 | 0.146 | 0.324 |
| rs1571911-2 | 0.040 | 0.065 | 0.537 |  | -0.004 | 0.066 | 0.950 |  | -0.003 | 0.065 | 0.962 |
| rs1571911-1 | 0.009 | 0.039 | 0.814 |  | -0.011 | 0.039 | 0.770 |  | -0.032 | 0.039 | 0.407 |
| rs1569414-2 | -0.133 | 0.063 | 0.036 |  | -0.070 | 0.064 | 0.269 |  | -0.007 | 0.063 | 0.913 |
| rs1569414-1 | 0.003 | 0.036 | 0.934 |  | 0.027 | 0.036 | 0.461 |  | -0.008 | 0.036 | 0.825 |
| rs1555136-2 | -0.056 | 0.052 | 0.279 |  | -0.055 | 0.052 | 0.290 |  | 0.066 | 0.052 | 0.205 |
| rs1555136-1 | -0.048 | 0.050 | 0.336 |  | -0.051 | 0.050 | 0.315 |  | 0.086 | 0.050 | 0.084 |
| rs1550270-2 | -0.044 | 0.060 | 0.462 |  | 0.018 | 0.060 | 0.763 |  | 0.038 | 0.060 | 0.525 |
| rs1550270-1 | -0.054 | 0.060 | 0.366 |  | 0.001 | 0.060 | 0.989 |  | -0.004 | 0.060 | 0.951 |
| rs1548607-2 | -0.003 | 0.059 | 0.965 |  | -0.033 | 0.059 | 0.578 |  | -0.043 | 0.059 | 0.471 |
| rs1548607-1 | 0.032 | 0.036 | 0.375 |  | 0.031 | 0.036 | 0.390 |  | -0.031 | 0.036 | 0.384 |
| rs1545161-2 | -0.107 | 0.050 | 0.032 |  | -0.123 | 0.050 | 0.015 |  | -0.041 | 0.050 | 0.413 |
| rs1545161-1 | -0.016 | 0.039 | 0.684 |  | 0.007 | 0.039 | 0.857 |  | 0.005 | 0.039 | 0.905 |
| rs1538658-2 | 0.015 | 0.093 | 0.873 |  | 0.017 | 0.093 | 0.859 |  | -0.085 | 0.092 | 0.357 |
| rs1538658-1 | 0.040 | 0.095 | 0.677 |  | 0.026 | 0.096 | 0.788 |  | -0.037 | 0.095 | 0.701 |
| rs1512450-2 | 0.096 | 0.055 | 0.081 |  | 0.089 | 0.055 | 0.107 |  | 0.024 | 0.055 | 0.668 |
| rs1512450-1 | -0.007 | 0.036 | 0.843 |  | -0.012 | 0.037 | 0.741 |  | 0.008 | 0.036 | 0.826 |
| rs150445982-2 | -0.003 | 0.721 | 0.997 |  | -0.100 | 0.724 | 0.890 |  | 0.491 | 0.719 | 0.495 |
| rs150445982-1 | -0.108 | 0.726 | 0.882 |  | -0.245 | 0.729 | 0.737 |  | 0.325 | 0.724 | 0.654 |
| rs1502199-2 | 0.042 | 0.065 | 0.515 |  | 0.073 | 0.065 | 0.266 |  | 0.102 | 0.065 | 0.114 |
| rs1502199-1 | 0.059 | 0.066 | 0.370 |  | 0.105 | 0.066 | 0.113 |  | 0.085 | 0.066 | 0.196 |
| rs150187724-2 | 0.049 | 0.685 | 0.943 |  | 0.209 | 0.689 | 0.761 |  | 0.558 | 0.683 | 0.414 |
| rs150187724-1 | 0.030 | 0.692 | 0.965 |  | 0.132 | 0.695 | 0.850 |  | 0.556 | 0.690 | 0.420 |
| rs1498507-2 | 0.015 | 0.048 | 0.751 |  | -0.009 | 0.048 | 0.851 |  | -0.026 | 0.048 | 0.585 |
| rs1498507-1 | 0.039 | 0.040 | 0.335 |  | 0.012 | 0.040 | 0.765 |  | 0.062 | 0.040 | 0.120 |
| rs149685227-2 | -0.644 | 0.881 | 0.465 |  | -0.595 | 0.885 | 0.501 |  | 0.091 | 0.878 | 0.918 |
| rs149685227-1 | -0.062 | 0.088 | 0.482 |  | -0.085 | 0.088 | 0.335 |  | 0.107 | 0.088 | 0.224 |
| rs149504726-2 | -0.016 | 0.144 | 0.910 |  | -0.026 | 0.145 | 0.856 |  | -0.017 | 0.144 | 0.906 |
| rs149082597-1 | -0.503 | 0.307 | 0.101 |  | -0.423 | 0.308 | 0.171 |  | -0.255 | 0.306 | 0.404 |
| rs149053406-2 | 0.188 | 0.459 | 0.682 |  | -0.033 | 0.461 | 0.942 |  | -0.388 | 0.458 | 0.397 |
| rs149053406-1 | 0.076 | 0.465 | 0.871 |  | -0.128 | 0.467 | 0.783 |  | -0.471 | 0.464 | 0.310 |
| rs148092711-1 | 0.228 | 0.239 | 0.341 |  | 0.273 | 0.240 | 0.256 |  | -0.215 | 0.239 | 0.368 |
| rs1475120-2 | -0.004 | 0.048 | 0.935 |  | 0.011 | 0.049 | 0.822 |  | -0.060 | 0.048 | 0.213 |
| rs1475120-1 | 0.001 | 0.039 | 0.981 |  | -0.002 | 0.039 | 0.957 |  | 0.031 | 0.039 | 0.419 |
| rs147176253-2 | 0.362 | 0.288 | 0.209 |  | 0.183 | 0.289 | 0.526 |  | 0.462 | 0.287 | 0.108 |
| rs147176253-1 | -0.032 | 0.057 | 0.576 |  | -0.075 | 0.057 | 0.189 |  | -0.038 | 0.057 | 0.506 |
| rs1471251-2 | -0.018 | 0.051 | 0.726 |  | 0.034 | 0.051 | 0.504 |  | 0.044 | 0.051 | 0.390 |
| rs1471251-1 | 0.021 | 0.049 | 0.662 |  | 0.071 | 0.049 | 0.149 |  | 0.039 | 0.049 | 0.427 |
| rs146447986-1 | -0.200 | 0.132 | 0.130 |  | -0.128 | 0.133 | 0.333 |  | -0.118 | 0.132 | 0.370 |
| rs1463598-2 | -0.025 | 0.051 | 0.619 |  | -0.028 | 0.051 | 0.576 |  | 0.006 | 0.051 | 0.911 |
| rs1463598-1 | 0.016 | 0.049 | 0.743 |  | -0.001 | 0.049 | 0.991 |  | 0.054 | 0.049 | 0.273 |
| rs146317210-2 | -1.818 | 1.318 | 0.168 |  | -1.799 | 1.324 | 0.174 |  | 0.773 | 1.314 | 0.556 |
| rs146317210-1 | -1.948 | 1.323 | 0.141 |  | -1.972 | 1.329 | 0.138 |  | 0.816 | 1.319 | 0.536 |
| rs1462151-2 | -0.068 | 0.122 | 0.573 |  | -0.109 | 0.122 | 0.373 |  | -0.362 | 0.122 | 0.003 |
| rs1462151-1 | 0.010 | 0.040 | 0.793 |  | -0.001 | 0.040 | 0.973 |  | 0.000 | 0.040 | 0.998 |
| rs146030699-2 | -0.438 | 0.841 | 0.603 |  | -0.137 | 0.845 | 0.871 |  | -0.447 | 0.838 | 0.594 |
| rs146030699-1 | -0.598 | 0.852 | 0.483 |  | -0.368 | 0.855 | 0.667 |  | -0.369 | 0.849 | 0.664 |
| rs1450851-2 | -0.088 | 0.056 | 0.115 |  | -0.130 | 0.056 | 0.021 |  | -0.081 | 0.056 | 0.145 |
| rs1450851-1 | -0.021 | 0.054 | 0.694 |  | -0.060 | 0.054 | 0.271 |  | -0.004 | 0.054 | 0.940 |
| rs144996521-1 | -0.041 | 0.232 | 0.860 |  | -0.140 | 0.233 | 0.549 |  | 0.058 | 0.232 | 0.803 |
| rs144832051-2 | -0.437 | 0.850 | 0.607 |  | -0.423 | 0.854 | 0.621 |  | 0.021 | 0.848 | 0.980 |
| rs144832051-1 | -0.250 | 0.853 | 0.770 |  | -0.216 | 0.857 | 0.801 |  | 0.073 | 0.851 | 0.931 |
| rs144488974-1 | 0.071 | 0.149 | 0.631 |  | -0.046 | 0.150 | 0.760 |  | 0.075 | 0.148 | 0.615 |
| rs144412371-2 | 0.411 | 0.534 | 0.442 |  | 0.375 | 0.536 | 0.484 |  | -0.703 | 0.532 | 0.187 |
| rs144412371-1 | 0.006 | 0.068 | 0.929 |  | 0.001 | 0.068 | 0.990 |  | 0.114 | 0.068 | 0.093 |
| rs144136543-2 | 0.394 | 0.533 | 0.459 |  | -0.136 | 0.535 | 0.800 |  | 0.099 | 0.531 | 0.853 |
| rs144136543-1 | 0.096 | 0.083 | 0.247 |  | 0.137 | 0.083 | 0.099 |  | -0.102 | 0.082 | 0.216 |
| rs1441165-2 | 0.011 | 0.050 | 0.830 |  | 0.059 | 0.050 | 0.238 |  | -0.061 | 0.050 | 0.223 |
| rs1441165-1 | -0.013 | 0.041 | 0.759 |  | 0.011 | 0.041 | 0.780 |  | -0.102 | 0.041 | 0.013 |
| rs1436138-2 | 0.027 | 0.053 | 0.609 |  | 0.002 | 0.053 | 0.973 |  | 0.073 | 0.052 | 0.163 |
| rs1436138-1 | -0.009 | 0.037 | 0.812 |  | 0.004 | 0.037 | 0.920 |  | 0.052 | 0.037 | 0.162 |
| rs143290532-2 | -1.204 | 1.183 | 0.309 |  | -1.038 | 1.188 | 0.382 |  | 0.567 | 1.179 | 0.631 |
| rs143290532-1 | -0.889 | 1.193 | 0.456 |  | -0.723 | 1.198 | 0.546 |  | 0.669 | 1.189 | 0.574 |
| rs143043662-2 | -0.068 | 0.137 | 0.619 |  | -0.133 | 0.138 | 0.336 |  | -0.101 | 0.137 | 0.462 |
| rs142971131-2 | 1.438 | 1.192 | 0.228 |  | 1.840 | 1.197 | 0.124 |  | 1.204 | 1.188 | 0.311 |
| rs142971131-1 | 0.106 | 0.147 | 0.470 |  | 0.149 | 0.148 | 0.313 |  | 0.013 | 0.147 | 0.927 |
| rs1428968-2 | -0.163 | 0.099 | 0.097 |  | -0.178 | 0.099 | 0.072 |  | -0.234 | 0.099 | 0.019 |
| rs1428968-1 | -0.096 | 0.102 | 0.347 |  | -0.100 | 0.102 | 0.329 |  | -0.162 | 0.102 | 0.115 |
| rs142343894-1 | 0.597 | 0.596 | 0.317 |  | 0.266 | 0.599 | 0.657 |  | 0.708 | 0.594 | 0.233 |
| rs142186557-2 | -0.987 | 0.690 | 0.153 |  | -0.371 | 0.693 | 0.593 |  | 0.238 | 0.688 | 0.729 |
| rs142186557-1 | -1.111 | 0.697 | 0.111 |  | -0.435 | 0.700 | 0.535 |  | 0.232 | 0.695 | 0.739 |
| rs1420996-2 | 0.020 | 0.071 | 0.782 |  | -0.020 | 0.072 | 0.779 |  | 0.018 | 0.071 | 0.797 |
| rs1420996-1 | -0.068 | 0.036 | 0.058 |  | -0.099 | 0.036 | 0.006 |  | -0.010 | 0.036 | 0.772 |
| rs142097791-1 | 0.174 | 0.698 | 0.804 |  | 0.111 | 0.701 | 0.874 |  | 0.077 | 0.696 | 0.912 |
| rs142072330-2 | -0.135 | 0.226 | 0.550 |  | -0.174 | 0.227 | 0.443 |  | -0.246 | 0.225 | 0.275 |
| rs142072330-1 | -0.172 | 0.229 | 0.454 |  | -0.188 | 0.230 | 0.414 |  | -0.241 | 0.228 | 0.291 |
| rs141892754-1 | -0.610 | 0.349 | 0.081 |  | -0.684 | 0.351 | 0.051 |  | -0.693 | 0.348 | 0.047 |
| rs141889567-1 | 0.342 | 0.544 | 0.530 |  | 0.508 | 0.547 | 0.353 |  | 0.291 | 0.543 | 0.592 |
| rs1415701-2 | -0.087 | 0.069 | 0.209 |  | 0.006 | 0.070 | 0.931 |  | -0.021 | 0.069 | 0.764 |
| rs1415701-1 | -0.086 | 0.069 | 0.215 |  | -0.033 | 0.069 | 0.630 |  | -0.029 | 0.069 | 0.674 |
| rs1414660-2 | -0.073 | 0.089 | 0.412 |  | -0.052 | 0.090 | 0.565 |  | -0.129 | 0.089 | 0.149 |
| rs1414660-1 | -0.032 | 0.091 | 0.725 |  | -0.016 | 0.092 | 0.865 |  | -0.081 | 0.091 | 0.370 |
| rs140760578-2 | -0.224 | 1.169 | 0.848 |  | -1.061 | 1.174 | 0.366 |  | 0.053 | 1.166 | 0.964 |
| rs140760578-1 | 0.085 | 0.123 | 0.490 |  | 0.201 | 0.123 | 0.103 |  | 0.230 | 0.122 | 0.060 |
| rs140191063-2 | -0.245 | 0.384 | 0.523 |  | -0.276 | 0.386 | 0.475 |  | -0.192 | 0.383 | 0.616 |
| rs140144628-2 | -0.143 | 0.162 | 0.379 |  | -0.064 | 0.163 | 0.694 |  | -0.163 | 0.162 | 0.315 |
| rs139603701-1 | 0.156 | 0.114 | 0.170 |  | 0.131 | 0.115 | 0.251 |  | 0.027 | 0.114 | 0.814 |
| rs139497-2 | -0.057 | 0.058 | 0.326 |  | -0.040 | 0.058 | 0.491 |  | -0.101 | 0.058 | 0.082 |
| rs139497-1 | -0.025 | 0.036 | 0.492 |  | 0.003 | 0.036 | 0.929 |  | 0.027 | 0.036 | 0.458 |
| rs139427417-1 | 0.110 | 0.124 | 0.373 |  | 0.099 | 0.125 | 0.426 |  | -0.155 | 0.124 | 0.210 |
| rs139326808-2 | -2.209 | 1.223 | 0.071 |  | -1.419 | 1.229 | 0.248 |  | -1.407 | 1.220 | 0.249 |
| rs139326808-1 | 0.089 | 0.161 | 0.581 |  | 0.009 | 0.161 | 0.956 |  | 0.011 | 0.160 | 0.944 |
| rs138852655-2 | -0.284 | 0.398 | 0.475 |  | -0.115 | 0.399 | 0.773 |  | -0.106 | 0.396 | 0.789 |
| rs138852655-1 | -0.281 | 0.402 | 0.485 |  | -0.094 | 0.404 | 0.816 |  | -0.098 | 0.401 | 0.807 |
| rs138818878-2 | -0.122 | 0.537 | 0.820 |  | 0.147 | 0.540 | 0.785 |  | -0.051 | 0.536 | 0.924 |
| rs138818878-1 | 0.140 | 0.543 | 0.797 |  | 0.398 | 0.545 | 0.466 |  | 0.213 | 0.542 | 0.694 |
| rs1386625-2 | -0.147 | 0.242 | 0.544 |  | -0.306 | 0.244 | 0.209 |  | -0.133 | 0.247 | 0.591 |
| rs1386625-1 | -0.131 | 0.246 | 0.594 |  | -0.280 | 0.247 | 0.258 |  | -0.127 | 0.251 | 0.613 |
| rs1385504-2 | -0.236 | 0.119 | 0.047 |  | -0.233 | 0.120 | 0.052 |  | -0.385 | 0.119 | 0.001 |
| rs1385504-1 | -0.046 | 0.040 | 0.254 |  | -0.055 | 0.040 | 0.175 |  | -0.059 | 0.040 | 0.140 |
| rs138273782-2 | -0.536 | 0.365 | 0.142 |  | -0.360 | 0.367 | 0.326 |  | -0.538 | 0.364 | 0.139 |
| rs138090420-2 | 0.524 | 0.543 | 0.334 |  | 0.714 | 0.545 | 0.190 |  | 0.842 | 0.541 | 0.120 |
| rs138090420-1 | 0.418 | 0.546 | 0.444 |  | 0.549 | 0.549 | 0.317 |  | 0.795 | 0.545 | 0.144 |
| rs137926455-1 | 0.029 | 0.094 | 0.754 |  | -0.011 | 0.094 | 0.903 |  | 0.091 | 0.093 | 0.332 |
| rs1358836-2 | -0.077 | 0.080 | 0.337 |  | -0.044 | 0.080 | 0.581 |  | -0.056 | 0.080 | 0.480 |
| rs1358836-1 | -0.077 | 0.082 | 0.348 |  | -0.052 | 0.083 | 0.529 |  | -0.088 | 0.082 | 0.286 |
| rs1353171-2 | -0.045 | 0.055 | 0.413 |  | -0.018 | 0.055 | 0.741 |  | 0.006 | 0.055 | 0.917 |
| rs1353171-1 | 0.007 | 0.037 | 0.853 |  | 0.013 | 0.037 | 0.733 |  | 0.014 | 0.037 | 0.695 |
| rs134613-2 | -0.030 | 0.055 | 0.586 |  | 0.006 | 0.055 | 0.920 |  | -0.025 | 0.055 | 0.653 |
| rs134613-1 | -0.029 | 0.055 | 0.596 |  | 0.009 | 0.055 | 0.870 |  | -0.021 | 0.055 | 0.698 |
| rs13429049-2 | -0.007 | 0.062 | 0.904 |  | 0.006 | 0.062 | 0.921 |  | 0.024 | 0.062 | 0.702 |
| rs13429049-1 | 0.011 | 0.063 | 0.868 |  | 0.019 | 0.064 | 0.766 |  | 0.044 | 0.063 | 0.488 |
| rs13384908-2 | 0.054 | 0.078 | 0.485 |  | 0.132 | 0.078 | 0.091 |  | -0.075 | 0.078 | 0.332 |
| rs13384908-1 | 0.057 | 0.081 | 0.482 |  | 0.157 | 0.081 | 0.054 |  | 0.007 | 0.080 | 0.931 |
| rs13379119-2 | -0.010 | 0.047 | 0.837 |  | 0.020 | 0.048 | 0.673 |  | -0.021 | 0.047 | 0.663 |
| rs13379119-1 | 0.002 | 0.043 | 0.959 |  | 0.028 | 0.043 | 0.516 |  | 0.023 | 0.043 | 0.583 |
| rs13336470-2 | -0.120 | 0.093 | 0.195 |  | -0.177 | 0.093 | 0.058 |  | -0.087 | 0.093 | 0.348 |
| rs13336470-1 | -0.090 | 0.095 | 0.343 |  | -0.151 | 0.095 | 0.112 |  | -0.088 | 0.095 | 0.353 |
| rs13336428-2 | 0.032 | 0.050 | 0.531 |  | -0.005 | 0.051 | 0.917 |  | 0.110 | 0.050 | 0.029 |
| rs13336428-1 | -0.051 | 0.038 | 0.181 |  | -0.046 | 0.038 | 0.227 |  | 0.001 | 0.038 | 0.983 |
| rs13334558-2 | -0.065 | 0.084 | 0.439 |  | -0.037 | 0.085 | 0.662 |  | -0.010 | 0.084 | 0.908 |
| rs13334558-1 | -0.102 | 0.037 | 0.006 |  | -0.101 | 0.037 | 0.006 |  | -0.046 | 0.037 | 0.211 |
| rs13303327-2 | -0.152 | 0.126 | 0.225 |  | -0.168 | 0.126 | 0.183 |  | -0.129 | 0.125 | 0.303 |
| rs13303327-1 | -0.105 | 0.126 | 0.403 |  | -0.157 | 0.126 | 0.214 |  | -0.146 | 0.125 | 0.244 |
| rs13291289-2 | -0.056 | 0.052 | 0.279 |  | -0.095 | 0.052 | 0.069 |  | -0.114 | 0.052 | 0.027 |
| rs13291289-1 | 0.008 | 0.049 | 0.867 |  | -0.025 | 0.050 | 0.614 |  | -0.086 | 0.049 | 0.082 |
| rs13267351-2 | -0.126 | 0.097 | 0.195 |  | -0.124 | 0.098 | 0.205 |  | -0.221 | 0.097 | 0.023 |
| rs13267351-1 | -0.097 | 0.101 | 0.336 |  | -0.077 | 0.101 | 0.447 |  | -0.238 | 0.100 | 0.018 |
| rs13225158-2 | 0.065 | 0.054 | 0.233 |  | 0.037 | 0.054 | 0.492 |  | -0.063 | 0.054 | 0.247 |
| rs13225158-1 | 0.052 | 0.037 | 0.156 |  | 0.021 | 0.037 | 0.570 |  | -0.060 | 0.037 | 0.105 |
| rs13220896-2 | 0.233 | 0.348 | 0.504 |  | 0.274 | 0.350 | 0.434 |  | 0.731 | 0.348 | 0.036 |
| rs13220896-1 | 0.038 | 0.062 | 0.544 |  | 0.041 | 0.062 | 0.510 |  | 0.034 | 0.062 | 0.583 |
| rs1320551-2 | 0.062 | 0.323 | 0.848 |  | 0.280 | 0.325 | 0.389 |  | 0.089 | 0.322 | 0.782 |
| rs1320551-1 | 0.027 | 0.057 | 0.641 |  | 0.010 | 0.057 | 0.861 |  | -0.043 | 0.057 | 0.452 |
| rs13201764-2 | -0.107 | 0.106 | 0.312 |  | -0.052 | 0.107 | 0.626 |  | -0.090 | 0.106 | 0.392 |
| rs13201764-1 | -0.142 | 0.109 | 0.194 |  | -0.090 | 0.110 | 0.411 |  | -0.110 | 0.109 | 0.314 |
| rs13195723-2 | 0.045 | 0.049 | 0.364 |  | 0.068 | 0.049 | 0.170 |  | -0.037 | 0.049 | 0.455 |
| rs13195723-1 | -0.036 | 0.039 | 0.368 |  | -0.049 | 0.040 | 0.215 |  | -0.076 | 0.039 | 0.055 |
| rs1318236-2 | 0.006 | 0.049 | 0.909 |  | -0.058 | 0.049 | 0.241 |  | -0.112 | 0.049 | 0.023 |
| rs1318236-1 | -0.003 | 0.041 | 0.950 |  | -0.045 | 0.041 | 0.268 |  | -0.088 | 0.041 | 0.030 |
| rs13179493-2 | 0.020 | 0.066 | 0.761 |  | -0.028 | 0.067 | 0.672 |  | -0.147 | 0.066 | 0.026 |
| rs13179493-1 | 0.033 | 0.067 | 0.623 |  | 0.018 | 0.067 | 0.783 |  | -0.074 | 0.067 | 0.268 |
| rs13138431-2 | -0.015 | 0.074 | 0.842 |  | -0.007 | 0.075 | 0.927 |  | 0.060 | 0.074 | 0.419 |
| rs13138431-1 | -0.039 | 0.036 | 0.281 |  | 0.004 | 0.036 | 0.905 |  | 0.038 | 0.036 | 0.298 |
| rs13088318-2 | 0.024 | 0.059 | 0.680 |  | 0.054 | 0.060 | 0.363 |  | 0.131 | 0.059 | 0.027 |
| rs13088318-1 | 0.030 | 0.059 | 0.611 |  | 0.060 | 0.059 | 0.314 |  | 0.064 | 0.059 | 0.278 |
| rs13072536-2 | -0.046 | 0.071 | 0.514 |  | -0.049 | 0.071 | 0.490 |  | -0.062 | 0.070 | 0.375 |
| rs13072536-1 | -0.036 | 0.072 | 0.617 |  | -0.058 | 0.073 | 0.426 |  | -0.001 | 0.072 | 0.987 |
| rs13070996-2 | 0.037 | 0.066 | 0.578 |  | 0.030 | 0.067 | 0.658 |  | 0.075 | 0.066 | 0.260 |
| rs13070996-1 | 0.031 | 0.036 | 0.397 |  | 0.035 | 0.036 | 0.327 |  | 0.070 | 0.036 | 0.051 |
| rs13043599-2 | -0.652 | 0.263 | 0.013 |  | -0.537 | 0.264 | 0.042 |  | 0.122 | 0.262 | 0.641 |
| rs13043599-1 | -0.626 | 0.266 | 0.019 |  | -0.493 | 0.268 | 0.065 |  | 0.118 | 0.266 | 0.656 |
| rs13017811-2 | -0.069 | 0.106 | 0.516 |  | 0.036 | 0.106 | 0.733 |  | -0.133 | 0.105 | 0.205 |
| rs13017811-1 | -0.021 | 0.038 | 0.593 |  | -0.020 | 0.039 | 0.610 |  | -0.005 | 0.038 | 0.894 |
| rs13002567-2 | 0.071 | 0.059 | 0.230 |  | 0.116 | 0.060 | 0.052 |  | 0.042 | 0.059 | 0.480 |
| rs13002567-1 | 0.105 | 0.059 | 0.076 |  | 0.124 | 0.060 | 0.038 |  | 0.090 | 0.059 | 0.130 |
| rs12958048-2 | -0.071 | 0.056 | 0.200 |  | -0.042 | 0.056 | 0.454 |  | -0.116 | 0.055 | 0.037 |
| rs12958048-1 | -0.050 | 0.036 | 0.166 |  | -0.043 | 0.036 | 0.231 |  | -0.036 | 0.036 | 0.311 |
| rs12956326-2 | -0.027 | 0.070 | 0.701 |  | -0.025 | 0.070 | 0.716 |  | -0.017 | 0.069 | 0.803 |
| rs12956326-1 | 0.012 | 0.071 | 0.864 |  | -0.022 | 0.071 | 0.759 |  | 0.000 | 0.071 | 0.996 |
| rs12954782-2 | 0.022 | 0.071 | 0.754 |  | 0.003 | 0.071 | 0.971 |  | -0.061 | 0.071 | 0.386 |
| rs12954782-1 | 0.023 | 0.073 | 0.748 |  | 0.005 | 0.073 | 0.942 |  | -0.059 | 0.073 | 0.418 |
| rs12951408-2 | -0.079 | 0.050 | 0.110 |  | -0.067 | 0.050 | 0.177 |  | -0.003 | 0.050 | 0.944 |
| rs12951408-1 | 0.004 | 0.045 | 0.933 |  | -0.032 | 0.045 | 0.477 |  | 0.006 | 0.045 | 0.901 |
| rs12948233-2 | -0.050 | 0.073 | 0.495 |  | -0.056 | 0.073 | 0.446 |  | -0.054 | 0.073 | 0.454 |
| rs12948233-1 | -0.031 | 0.036 | 0.386 |  | -0.070 | 0.036 | 0.054 |  | -0.053 | 0.036 | 0.140 |
| rs12945403-2 | -0.063 | 0.055 | 0.255 |  | -0.079 | 0.056 | 0.157 |  | 0.015 | 0.055 | 0.784 |
| rs12945403-1 | -0.032 | 0.054 | 0.557 |  | -0.066 | 0.054 | 0.226 |  | 0.040 | 0.054 | 0.458 |
| rs12943370-2 | 0.034 | 0.053 | 0.525 |  | 0.078 | 0.054 | 0.146 |  | 0.036 | 0.053 | 0.504 |
| rs12943370-1 | 0.027 | 0.038 | 0.473 |  | 0.019 | 0.038 | 0.609 |  | -0.006 | 0.037 | 0.877 |
| rs12942736-2 | -0.261 | 0.080 | 0.001 |  | -0.244 | 0.081 | 0.002 |  | -0.142 | 0.080 | 0.076 |
| rs12942736-1 | -0.028 | 0.040 | 0.478 |  | -0.028 | 0.040 | 0.483 |  | 0.041 | 0.040 | 0.303 |
| rs12938040-2 | -0.062 | 0.347 | 0.858 |  | -0.159 | 0.349 | 0.648 |  | 0.153 | 0.346 | 0.659 |
| rs12938040-1 | -0.114 | 0.353 | 0.747 |  | -0.176 | 0.354 | 0.619 |  | 0.144 | 0.352 | 0.682 |
| rs1293672-2 | 0.015 | 0.151 | 0.920 |  | -0.058 | 0.152 | 0.702 |  | 0.075 | 0.151 | 0.617 |
| rs1293672-1 | 0.012 | 0.042 | 0.783 |  | 0.010 | 0.043 | 0.818 |  | -0.004 | 0.042 | 0.927 |
| rs1286662-2 | -0.085 | 0.087 | 0.326 |  | -0.110 | 0.087 | 0.206 |  | -0.014 | 0.087 | 0.871 |
| rs1286662-1 | 0.005 | 0.038 | 0.888 |  | 0.007 | 0.038 | 0.846 |  | 0.041 | 0.037 | 0.279 |
| rs1286075-2 | -0.088 | 0.087 | 0.314 |  | -0.122 | 0.087 | 0.163 |  | -0.218 | 0.087 | 0.012 |
| rs1286075-1 | -0.069 | 0.090 | 0.443 |  | -0.086 | 0.090 | 0.342 |  | -0.211 | 0.090 | 0.019 |
| rs12855887-2 | -0.069 | 0.049 | 0.159 |  | -0.056 | 0.049 | 0.258 |  | -0.053 | 0.049 | 0.281 |
| rs12855887-1 | -0.002 | 0.044 | 0.965 |  | -0.004 | 0.044 | 0.927 |  | 0.051 | 0.044 | 0.248 |
| rs1284200-2 | -0.058 | 0.220 | 0.792 |  | -0.225 | 0.221 | 0.307 |  | 0.001 | 0.219 | 0.997 |
| rs1284200-1 | 0.004 | 0.222 | 0.984 |  | -0.141 | 0.222 | 0.526 |  | 0.080 | 0.221 | 0.718 |
| rs12811685-2 | 0.012 | 0.057 | 0.828 |  | 0.063 | 0.057 | 0.267 |  | 0.026 | 0.057 | 0.644 |
| rs12811685-1 | -0.001 | 0.037 | 0.978 |  | -0.018 | 0.037 | 0.627 |  | 0.019 | 0.036 | 0.593 |
| rs12776318-2 | -0.007 | 0.057 | 0.903 |  | 0.043 | 0.057 | 0.452 |  | -0.018 | 0.057 | 0.752 |
| rs12776318-1 | -0.054 | 0.056 | 0.341 |  | -0.003 | 0.057 | 0.961 |  | -0.019 | 0.056 | 0.736 |
| rs12769962-2 | 0.231 | 0.106 | 0.029 |  | 0.182 | 0.106 | 0.087 |  | 0.117 | 0.105 | 0.268 |
| rs12769962-1 | 0.030 | 0.039 | 0.437 |  | -0.007 | 0.039 | 0.860 |  | -0.016 | 0.038 | 0.669 |
| rs12756373-2 | -0.208 | 0.211 | 0.323 |  | -0.061 | 0.212 | 0.772 |  | 0.037 | 0.210 | 0.861 |
| rs12756373-1 | -0.171 | 0.215 | 0.426 |  | 0.016 | 0.216 | 0.941 |  | 0.067 | 0.214 | 0.754 |
| rs12756110-2 | -0.515 | 0.223 | 0.021 |  | -0.517 | 0.224 | 0.021 |  | 0.029 | 0.222 | 0.897 |
| rs12756110-1 | -0.470 | 0.225 | 0.037 |  | -0.443 | 0.226 | 0.050 |  | -0.006 | 0.225 | 0.980 |
| rs1272131-2 | -0.036 | 0.051 | 0.472 |  | -0.069 | 0.051 | 0.178 |  | -0.081 | 0.051 | 0.111 |
| rs1272131-1 | -0.032 | 0.048 | 0.500 |  | -0.062 | 0.048 | 0.193 |  | -0.058 | 0.048 | 0.219 |
| rs1268119-2 | -0.049 | 0.072 | 0.493 |  | -0.012 | 0.072 | 0.871 |  | -0.020 | 0.071 | 0.776 |
| rs1268119-1 | -0.020 | 0.037 | 0.588 |  | 0.005 | 0.037 | 0.883 |  | -0.007 | 0.037 | 0.852 |
| rs12673167-2 | 0.071 | 0.061 | 0.250 |  | 0.084 | 0.062 | 0.173 |  | 0.059 | 0.061 | 0.334 |
| rs12673167-1 | 0.050 | 0.061 | 0.407 |  | 0.076 | 0.061 | 0.212 |  | 0.087 | 0.061 | 0.152 |
| rs12673062-2 | 0.081 | 0.078 | 0.302 |  | 0.098 | 0.079 | 0.212 |  | 0.193 | 0.078 | 0.014 |
| rs12673062-1 | 0.041 | 0.080 | 0.611 |  | 0.063 | 0.081 | 0.433 |  | 0.070 | 0.080 | 0.385 |
| rs12616772-2 | -0.040 | 0.055 | 0.460 |  | -0.010 | 0.055 | 0.855 |  | -0.034 | 0.055 | 0.532 |
| rs12616772-1 | -0.019 | 0.054 | 0.721 |  | -0.004 | 0.054 | 0.941 |  | -0.032 | 0.054 | 0.557 |
| rs12614608-2 | -0.140 | 0.060 | 0.020 |  | -0.132 | 0.060 | 0.029 |  | -0.109 | 0.060 | 0.071 |
| rs12614608-1 | -0.005 | 0.036 | 0.891 |  | 0.007 | 0.037 | 0.845 |  | -0.025 | 0.036 | 0.490 |
| rs12545602-2 | -0.098 | 0.121 | 0.420 |  | -0.147 | 0.122 | 0.228 |  | -0.009 | 0.122 | 0.939 |
| rs12545602-1 | -0.140 | 0.125 | 0.261 |  | -0.165 | 0.125 | 0.188 |  | -0.071 | 0.125 | 0.572 |
| rs12534970-2 | 0.044 | 0.049 | 0.374 |  | 0.064 | 0.049 | 0.192 |  | -0.042 | 0.049 | 0.387 |
| rs12534970-1 | -0.043 | 0.041 | 0.299 |  | -0.023 | 0.041 | 0.584 |  | -0.089 | 0.041 | 0.031 |
| rs12487905-2 | -0.069 | 0.078 | 0.382 |  | -0.093 | 0.079 | 0.237 |  | -0.135 | 0.078 | 0.085 |
| rs12487905-1 | -0.022 | 0.081 | 0.788 |  | -0.021 | 0.082 | 0.800 |  | -0.133 | 0.081 | 0.101 |
| rs12482821-2 | 0.986 | 1.260 | 0.434 |  | 0.855 | 1.265 | 0.499 |  | 0.795 | 1.256 | 0.527 |
| rs12482821-1 | 0.888 | 1.262 | 0.482 |  | 0.730 | 1.268 | 0.565 |  | 0.793 | 1.258 | 0.528 |
| rs12469063-2 | -0.037 | 0.072 | 0.611 |  | -0.026 | 0.073 | 0.724 |  | 0.012 | 0.072 | 0.867 |
| rs12469063-1 | -0.095 | 0.073 | 0.196 |  | -0.060 | 0.074 | 0.417 |  | 0.001 | 0.073 | 0.986 |
| rs12462380-2 | -0.039 | 0.049 | 0.433 |  | -0.051 | 0.050 | 0.302 |  | -0.107 | 0.049 | 0.029 |
| rs12462380-1 | 0.007 | 0.046 | 0.875 |  | -0.017 | 0.047 | 0.710 |  | -0.043 | 0.046 | 0.353 |
| rs12460389-2 | 0.166 | 0.099 | 0.095 |  | 0.177 | 0.100 | 0.076 |  | 0.095 | 0.099 | 0.337 |
| rs12460389-1 | 0.201 | 0.101 | 0.047 |  | 0.244 | 0.102 | 0.017 |  | 0.098 | 0.101 | 0.333 |
| rs12452590-2 | -0.030 | 0.055 | 0.591 |  | -0.030 | 0.055 | 0.584 |  | -0.009 | 0.055 | 0.873 |
| rs12452590-1 | -0.040 | 0.053 | 0.443 |  | -0.031 | 0.053 | 0.564 |  | -0.001 | 0.053 | 0.977 |
| rs12452440-2 | 0.020 | 0.049 | 0.685 |  | 0.010 | 0.049 | 0.846 |  | 0.045 | 0.049 | 0.363 |
| rs12452440-1 | -0.050 | 0.038 | 0.183 |  | -0.042 | 0.038 | 0.265 |  | -0.033 | 0.038 | 0.385 |
| rs12441073-2 | 0.003 | 0.070 | 0.970 |  | -0.007 | 0.070 | 0.923 |  | -0.080 | 0.069 | 0.246 |
| rs12441073-1 | 0.058 | 0.071 | 0.413 |  | 0.049 | 0.071 | 0.494 |  | 0.013 | 0.071 | 0.855 |
| rs12427846-2 | 0.195 | 0.078 | 0.013 |  | 0.173 | 0.079 | 0.028 |  | 0.076 | 0.078 | 0.330 |
| rs12427846-1 | 0.199 | 0.080 | 0.013 |  | 0.134 | 0.080 | 0.095 |  | 0.102 | 0.080 | 0.201 |
| rs12379417-2 | 0.037 | 0.056 | 0.505 |  | 0.059 | 0.056 | 0.288 |  | 0.087 | 0.056 | 0.116 |
| rs12379417-1 | 0.009 | 0.055 | 0.874 |  | 0.054 | 0.055 | 0.332 |  | 0.023 | 0.055 | 0.683 |
| rs12340775-2 | -0.261 | 0.336 | 0.437 |  | -0.245 | 0.337 | 0.467 |  | -0.288 | 0.335 | 0.390 |
| rs12340775-1 | -0.032 | 0.057 | 0.573 |  | 0.006 | 0.057 | 0.911 |  | 0.043 | 0.057 | 0.447 |
| rs12326005-2 | -0.080 | 0.048 | 0.095 |  | -0.111 | 0.048 | 0.020 |  | -0.031 | 0.047 | 0.514 |
| rs12326005-1 | -0.100 | 0.042 | 0.016 |  | -0.086 | 0.042 | 0.039 |  | -0.102 | 0.041 | 0.014 |
| rs12325187-2 | 0.067 | 0.070 | 0.342 |  | 0.049 | 0.071 | 0.492 |  | 0.068 | 0.070 | 0.331 |
| rs12325187-1 | -0.005 | 0.072 | 0.946 |  | 0.003 | 0.072 | 0.972 |  | 0.009 | 0.071 | 0.896 |
| rs12323717-2 | -0.015 | 0.055 | 0.785 |  | 0.024 | 0.055 | 0.668 |  | 0.011 | 0.055 | 0.834 |
| rs12323717-1 | -0.021 | 0.054 | 0.697 |  | 0.025 | 0.054 | 0.645 |  | 0.017 | 0.053 | 0.751 |
| rs12305097-2 | 0.019 | 0.051 | 0.700 |  | 0.025 | 0.051 | 0.628 |  | -0.040 | 0.050 | 0.424 |
| rs12305097-1 | 0.039 | 0.038 | 0.313 |  | 0.032 | 0.039 | 0.411 |  | 0.027 | 0.038 | 0.482 |
| rs12300425-2 | 0.012 | 0.049 | 0.800 |  | 0.033 | 0.049 | 0.501 |  | -0.056 | 0.049 | 0.255 |
| rs12300425-1 | 0.002 | 0.038 | 0.962 |  | 0.043 | 0.038 | 0.267 |  | -0.034 | 0.038 | 0.368 |
| rs1229984-2 | 0.267 | 0.157 | 0.089 |  | 0.335 | 0.157 | 0.033 |  | -0.153 | 0.156 | 0.326 |
| rs1229984-1 | 0.272 | 0.154 | 0.077 |  | 0.277 | 0.154 | 0.072 |  | -0.127 | 0.153 | 0.409 |
| rs12254582-2 | -0.071 | 0.067 | 0.285 |  | -0.027 | 0.067 | 0.681 |  | 0.150 | 0.067 | 0.024 |
| rs12254582-1 | -0.036 | 0.036 | 0.327 |  | -0.032 | 0.036 | 0.380 |  | -0.001 | 0.036 | 0.987 |
| rs12251299-2 | 0.107 | 0.131 | 0.415 |  | 0.072 | 0.132 | 0.585 |  | -0.113 | 0.131 | 0.388 |
| rs12251299-1 | 0.020 | 0.044 | 0.656 |  | 0.025 | 0.044 | 0.567 |  | -0.077 | 0.044 | 0.080 |
| rs12228756-2 | 0.050 | 0.198 | 0.800 |  | 0.260 | 0.199 | 0.193 |  | 0.219 | 0.198 | 0.268 |
| rs12228756-1 | 0.062 | 0.202 | 0.760 |  | 0.311 | 0.202 | 0.125 |  | 0.224 | 0.201 | 0.265 |
| rs12218358-2 | 0.057 | 0.050 | 0.253 |  | 0.069 | 0.050 | 0.171 |  | -0.007 | 0.050 | 0.888 |
| rs12218358-1 | 0.033 | 0.039 | 0.404 |  | 0.052 | 0.039 | 0.187 |  | -0.002 | 0.039 | 0.951 |
| rs12214204-2 | 0.006 | 0.110 | 0.958 |  | -0.038 | 0.111 | 0.732 |  | -0.002 | 0.110 | 0.984 |
| rs12214204-1 | -0.053 | 0.040 | 0.191 |  | -0.041 | 0.041 | 0.310 |  | -0.023 | 0.040 | 0.571 |
| rs12213392-2 | -0.012 | 0.050 | 0.807 |  | 0.016 | 0.050 | 0.746 |  | -0.015 | 0.050 | 0.767 |
| rs12213392-1 | -0.040 | 0.038 | 0.299 |  | 0.016 | 0.038 | 0.677 |  | 0.002 | 0.038 | 0.966 |
| rs12208105-2 | 0.049 | 0.054 | 0.363 |  | 0.080 | 0.054 | 0.141 |  | 0.092 | 0.054 | 0.089 |
| rs12208105-1 | -0.015 | 0.050 | 0.763 |  | 0.042 | 0.050 | 0.406 |  | 0.034 | 0.050 | 0.494 |
| rs12206717-2 | 0.111 | 0.312 | 0.723 |  | 0.109 | 0.313 | 0.727 |  | 0.343 | 0.311 | 0.270 |
| rs12206717-1 | 0.010 | 0.316 | 0.976 |  | 0.053 | 0.318 | 0.867 |  | 0.316 | 0.315 | 0.316 |
| rs12154446-2 | -0.079 | 0.070 | 0.258 |  | -0.032 | 0.070 | 0.651 |  | -0.067 | 0.070 | 0.341 |
| rs12154446-1 | -0.060 | 0.071 | 0.403 |  | 0.024 | 0.072 | 0.736 |  | -0.023 | 0.071 | 0.746 |
| rs12149673-2 | -0.024 | 0.058 | 0.682 |  | 0.013 | 0.058 | 0.826 |  | 0.004 | 0.058 | 0.943 |
| rs12149673-1 | -0.010 | 0.058 | 0.861 |  | 0.021 | 0.059 | 0.717 |  | 0.045 | 0.058 | 0.444 |
| rs12135380-2 | -0.054 | 0.054 | 0.316 |  | -0.066 | 0.054 | 0.222 |  | -0.082 | 0.054 | 0.127 |
| rs12135380-1 | -0.024 | 0.053 | 0.653 |  | -0.021 | 0.053 | 0.692 |  | -0.050 | 0.053 | 0.345 |
| rs12134534-2 | -0.083 | 0.050 | 0.100 |  | -0.093 | 0.051 | 0.066 |  | -0.028 | 0.050 | 0.584 |
| rs12134534-1 | 0.004 | 0.039 | 0.921 |  | 0.000 | 0.039 | 0.992 |  | 0.027 | 0.039 | 0.492 |
| rs12112607-2 | 0.039 | 0.644 | 0.952 |  | 0.071 | 0.646 | 0.913 |  | -0.258 | 0.642 | 0.687 |
| rs12112607-1 | 0.113 | 0.652 | 0.862 |  | 0.106 | 0.655 | 0.872 |  | -0.198 | 0.650 | 0.761 |
| rs12080074-2 | -0.051 | 0.055 | 0.350 |  | -0.059 | 0.055 | 0.287 |  | 0.032 | 0.055 | 0.557 |
| rs12080074-1 | -0.009 | 0.054 | 0.873 |  | -0.002 | 0.054 | 0.976 |  | 0.066 | 0.054 | 0.222 |
| rs1206755-2 | 0.008 | 0.050 | 0.879 |  | 0.011 | 0.050 | 0.831 |  | -0.003 | 0.049 | 0.957 |
| rs1206755-1 | 0.033 | 0.044 | 0.457 |  | 0.020 | 0.045 | 0.651 |  | 0.061 | 0.044 | 0.168 |
| rs12042197-2 | -0.059 | 0.077 | 0.448 |  | 0.008 | 0.078 | 0.920 |  | -0.030 | 0.077 | 0.696 |
| rs12042197-1 | -0.060 | 0.036 | 0.100 |  | -0.072 | 0.036 | 0.048 |  | -0.077 | 0.036 | 0.033 |
| rs12034786-2 | 0.049 | 0.072 | 0.493 |  | 0.050 | 0.072 | 0.488 |  | -0.027 | 0.072 | 0.706 |
| rs12034786-1 | -0.020 | 0.036 | 0.576 |  | 0.012 | 0.036 | 0.750 |  | 0.002 | 0.036 | 0.962 |
| rs12031054-2 | -0.038 | 0.060 | 0.523 |  | -0.011 | 0.060 | 0.849 |  | -0.074 | 0.060 | 0.214 |
| rs12031054-1 | 0.050 | 0.036 | 0.167 |  | 0.059 | 0.037 | 0.104 |  | 0.006 | 0.036 | 0.870 |
| rs11946517-2 | -0.070 | 0.051 | 0.173 |  | -0.010 | 0.052 | 0.843 |  | 0.023 | 0.051 | 0.648 |
| rs11946517-1 | 0.015 | 0.048 | 0.755 |  | 0.036 | 0.048 | 0.462 |  | 0.041 | 0.048 | 0.396 |
| rs11934731-2 | -0.056 | 0.058 | 0.334 |  | -0.019 | 0.058 | 0.743 |  | -0.045 | 0.058 | 0.431 |
| rs11934731-1 | -0.006 | 0.057 | 0.915 |  | 0.048 | 0.057 | 0.406 |  | 0.015 | 0.057 | 0.794 |
| rs11915970-2 | 0.033 | 0.141 | 0.817 |  | 0.021 | 0.141 | 0.882 |  | 0.140 | 0.140 | 0.319 |
| rs11915970-1 | 0.113 | 0.143 | 0.431 |  | 0.083 | 0.144 | 0.567 |  | 0.264 | 0.143 | 0.065 |
| rs1188292-2 | 0.073 | 0.099 | 0.457 |  | 0.066 | 0.099 | 0.503 |  | 0.055 | 0.098 | 0.578 |
| rs1188292-1 | 0.067 | 0.039 | 0.080 |  | 0.066 | 0.039 | 0.090 |  | -0.017 | 0.038 | 0.651 |
| rs11881367-2 | 0.254 | 0.148 | 0.087 |  | 0.239 | 0.149 | 0.108 |  | 0.175 | 0.148 | 0.237 |
| rs11881367-1 | 0.261 | 0.147 | 0.075 |  | 0.229 | 0.147 | 0.120 |  | 0.165 | 0.146 | 0.259 |
| rs11880992-2 | -0.015 | 0.051 | 0.772 |  | 0.041 | 0.051 | 0.427 |  | 0.016 | 0.051 | 0.756 |
| rs11880992-1 | -0.041 | 0.038 | 0.276 |  | -0.019 | 0.038 | 0.621 |  | -0.050 | 0.038 | 0.182 |
| rs11875132-2 | -0.049 | 0.049 | 0.317 |  | -0.050 | 0.050 | 0.309 |  | -0.045 | 0.049 | 0.365 |
| rs11875132-1 | -0.047 | 0.039 | 0.237 |  | -0.021 | 0.040 | 0.600 |  | -0.037 | 0.039 | 0.347 |
| rs11866031-2 | 0.092 | 0.111 | 0.406 |  | 0.152 | 0.112 | 0.173 |  | 0.048 | 0.111 | 0.663 |
| rs11866031-1 | 0.037 | 0.040 | 0.366 |  | 0.001 | 0.041 | 0.974 |  | 0.010 | 0.040 | 0.796 |
| rs11858857-2 | -0.129 | 0.200 | 0.518 |  | 0.009 | 0.201 | 0.964 |  | -0.216 | 0.199 | 0.278 |
| rs11858857-1 | -0.149 | 0.205 | 0.467 |  | -0.010 | 0.206 | 0.961 |  | -0.263 | 0.204 | 0.198 |
| rs118172483-2 | 0.692 | 0.381 | 0.069 |  | 0.640 | 0.382 | 0.094 |  | -0.070 | 0.379 | 0.854 |
| rs118172483-1 | 0.652 | 0.385 | 0.090 |  | 0.670 | 0.386 | 0.083 |  | -0.103 | 0.383 | 0.787 |
| rs11814082-2 | -0.059 | 0.093 | 0.528 |  | -0.071 | 0.094 | 0.448 |  | 0.151 | 0.093 | 0.106 |
| rs11814082-1 | -0.044 | 0.095 | 0.642 |  | -0.073 | 0.096 | 0.445 |  | 0.165 | 0.095 | 0.083 |
| rs118115924-2 | -0.992 | 1.192 | 0.405 |  | -0.385 | 1.197 | 0.748 |  | 0.531 | 1.189 | 0.655 |
| rs118115924-1 | -0.441 | 0.140 | 0.002 |  | -0.354 | 0.141 | 0.012 |  | -0.527 | 0.140 | 0.000 |
| rs118009556-2 | -0.659 | 0.491 | 0.180 |  | -0.385 | 0.493 | 0.436 |  | -0.545 | 0.490 | 0.266 |
| rs118009556-1 | -0.582 | 0.495 | 0.240 |  | -0.288 | 0.497 | 0.562 |  | -0.428 | 0.493 | 0.386 |
| rs11792038-2 | 0.077 | 0.125 | 0.538 |  | 0.074 | 0.125 | 0.554 |  | -0.030 | 0.124 | 0.809 |
| rs11792038-1 | 0.028 | 0.041 | 0.489 |  | 0.035 | 0.041 | 0.397 |  | 0.024 | 0.041 | 0.560 |
| rs11763267-2 | 0.117 | 0.099 | 0.237 |  | 0.147 | 0.100 | 0.139 |  | -0.033 | 0.099 | 0.740 |
| rs11763267-1 | 0.004 | 0.038 | 0.918 |  | 0.024 | 0.038 | 0.537 |  | 0.036 | 0.038 | 0.339 |
| rs11759018-2 | 0.041 | 0.062 | 0.506 |  | 0.009 | 0.062 | 0.881 |  | 0.025 | 0.062 | 0.683 |
| rs11759018-1 | 0.031 | 0.037 | 0.411 |  | -0.005 | 0.037 | 0.892 |  | 0.014 | 0.037 | 0.697 |
| rs11754632-2 | 0.040 | 0.050 | 0.416 |  | 0.076 | 0.050 | 0.131 |  | -0.055 | 0.050 | 0.268 |
| rs11754632-1 | 0.033 | 0.045 | 0.464 |  | 0.063 | 0.045 | 0.167 |  | 0.004 | 0.045 | 0.927 |
| rs117481343-2 | -0.132 | 0.844 | 0.875 |  | -0.395 | 0.847 | 0.641 |  | -1.032 | 0.841 | 0.220 |
| rs117481343-1 | -0.127 | 0.847 | 0.881 |  | -0.383 | 0.850 | 0.652 |  | -1.043 | 0.844 | 0.216 |
| rs11743474-2 | 0.056 | 0.096 | 0.559 |  | 0.105 | 0.097 | 0.277 |  | 0.080 | 0.096 | 0.407 |
| rs11743474-1 | 0.026 | 0.039 | 0.496 |  | 0.034 | 0.039 | 0.382 |  | -0.006 | 0.039 | 0.879 |
| rs11741390-2 | 0.060 | 0.049 | 0.226 |  | 0.050 | 0.050 | 0.310 |  | 0.030 | 0.049 | 0.541 |
| rs11741390-1 | 0.014 | 0.045 | 0.752 |  | 0.007 | 0.046 | 0.885 |  | -0.003 | 0.045 | 0.954 |
| rs117400692-2 | 1.874 | 1.248 | 0.133 |  | 1.221 | 1.254 | 0.330 |  | -1.911 | 1.244 | 0.125 |
| rs117400692-1 | 2.004 | 1.249 | 0.109 |  | 1.323 | 1.254 | 0.292 |  | -1.736 | 1.245 | 0.163 |
| rs11736866-2 | 0.114 | 0.105 | 0.274 |  | 0.161 | 0.105 | 0.124 |  | 0.052 | 0.104 | 0.618 |
| rs11736866-1 | 0.112 | 0.107 | 0.296 |  | 0.200 | 0.107 | 0.063 |  | 0.080 | 0.107 | 0.450 |
| rs11731267-2 | -0.389 | 0.120 | 0.001 |  | -0.410 | 0.120 | 0.001 |  | -0.247 | 0.119 | 0.038 |
| rs11731267-1 | -0.411 | 0.123 | 0.001 |  | -0.437 | 0.123 | 0.000 |  | -0.273 | 0.123 | 0.026 |
| rs11729023-2 | -0.210 | 0.135 | 0.120 |  | -0.160 | 0.136 | 0.239 |  | 0.031 | 0.135 | 0.816 |
| rs11729023-1 | -0.196 | 0.139 | 0.157 |  | -0.170 | 0.139 | 0.223 |  | 0.076 | 0.138 | 0.581 |
| rs11726436-2 | -0.022 | 0.053 | 0.679 |  | -0.016 | 0.053 | 0.771 |  | 0.011 | 0.053 | 0.836 |
| rs11726436-1 | -0.023 | 0.045 | 0.609 |  | 0.006 | 0.045 | 0.890 |  | -0.002 | 0.045 | 0.960 |
| rs117208012-2 | -0.241 | 0.715 | 0.736 |  | -0.684 | 0.718 | 0.341 |  | 0.343 | 0.713 | 0.630 |
| rs117208012-1 | 0.089 | 0.110 | 0.420 |  | 0.000 | 0.111 | 0.997 |  | 0.169 | 0.110 | 0.125 |
| rs117204589-2 | -1.546 | 0.902 | 0.087 |  | -1.790 | 0.906 | 0.048 |  | -0.392 | 0.899 | 0.663 |
| rs117204589-1 | -1.436 | 0.907 | 0.113 |  | -1.693 | 0.911 | 0.063 |  | -0.500 | 0.904 | 0.581 |
| rs11719201-2 | -0.125 | 0.080 | 0.117 |  | -0.127 | 0.080 | 0.111 |  | -0.136 | 0.079 | 0.087 |
| rs11719201-1 | -0.008 | 0.037 | 0.830 |  | 0.022 | 0.037 | 0.563 |  | 0.005 | 0.037 | 0.891 |
| rs11712061-2 | 0.031 | 0.050 | 0.533 |  | 0.034 | 0.050 | 0.500 |  | -0.089 | 0.050 | 0.074 |
| rs11712061-1 | -0.014 | 0.038 | 0.715 |  | 0.026 | 0.038 | 0.504 |  | -0.009 | 0.038 | 0.806 |
| rs117111740-2 | -0.095 | 0.833 | 0.909 |  | -0.344 | 0.837 | 0.681 |  | 0.356 | 0.831 | 0.668 |
| rs117111740-1 | -0.017 | 0.086 | 0.846 |  | -0.039 | 0.086 | 0.653 |  | -0.071 | 0.086 | 0.408 |
| rs117028614-2 | -0.417 | 0.422 | 0.323 |  | -0.691 | 0.424 | 0.103 |  | -0.940 | 0.421 | 0.026 |
| rs117026123-2 | -0.700 | 0.380 | 0.065 |  | -0.534 | 0.381 | 0.162 |  | 0.322 | 0.378 | 0.395 |
| rs117026123-1 | -0.066 | 0.065 | 0.314 |  | -0.076 | 0.066 | 0.245 |  | -0.041 | 0.065 | 0.528 |
| rs11696009-2 | -0.042 | 0.056 | 0.455 |  | -0.070 | 0.056 | 0.208 |  | -0.049 | 0.055 | 0.379 |
| rs11696009-1 | 0.028 | 0.055 | 0.609 |  | -0.018 | 0.055 | 0.749 |  | 0.055 | 0.055 | 0.312 |
| rs116918730-2 | -0.231 | 0.312 | 0.460 |  | -0.178 | 0.314 | 0.571 |  | -0.638 | 0.312 | 0.041 |
| rs116918730-1 | -0.020 | 0.061 | 0.738 |  | 0.015 | 0.061 | 0.812 |  | 0.034 | 0.061 | 0.572 |
| rs11688492-2 | 0.002 | 0.049 | 0.968 |  | 0.012 | 0.049 | 0.804 |  | -0.073 | 0.049 | 0.134 |
| rs11688492-1 | 0.010 | 0.040 | 0.807 |  | -0.001 | 0.040 | 0.986 |  | -0.076 | 0.040 | 0.055 |
| rs116848211-2 | 0.455 | 0.715 | 0.524 |  | -0.113 | 0.718 | 0.874 |  | 0.336 | 0.713 | 0.638 |
| rs116848211-1 | 0.534 | 0.716 | 0.456 |  | -0.016 | 0.720 | 0.982 |  | 0.305 | 0.715 | 0.670 |
| rs11679303-2 | -0.052 | 0.087 | 0.546 |  | -0.001 | 0.087 | 0.988 |  | -0.079 | 0.086 | 0.361 |
| rs11679303-1 | -0.025 | 0.037 | 0.505 |  | -0.025 | 0.038 | 0.501 |  | -0.041 | 0.037 | 0.267 |
| rs11677953-2 | -0.116 | 0.050 | 0.021 |  | -0.102 | 0.051 | 0.045 |  | -0.091 | 0.050 | 0.070 |
| rs11677953-1 | -0.060 | 0.049 | 0.220 |  | -0.018 | 0.049 | 0.718 |  | -0.081 | 0.049 | 0.099 |
| rs11675489-2 | -0.042 | 0.051 | 0.405 |  | -0.061 | 0.051 | 0.232 |  | 0.017 | 0.051 | 0.735 |
| rs11675489-1 | -0.022 | 0.039 | 0.578 |  | -0.028 | 0.039 | 0.466 |  | 0.035 | 0.039 | 0.365 |
| rs11670562-2 | -0.025 | 0.071 | 0.720 |  | -0.040 | 0.071 | 0.576 |  | 0.019 | 0.071 | 0.791 |
| rs11670562-1 | -0.047 | 0.073 | 0.515 |  | -0.054 | 0.073 | 0.458 |  | 0.037 | 0.072 | 0.612 |
| rs11668064-2 | 0.033 | 0.061 | 0.588 |  | 0.010 | 0.061 | 0.875 |  | 0.067 | 0.061 | 0.273 |
| rs11668064-1 | -0.037 | 0.036 | 0.299 |  | -0.050 | 0.036 | 0.170 |  | -0.026 | 0.036 | 0.461 |
| rs116587722-2 | -0.699 | 0.277 | 0.012 |  | -0.713 | 0.278 | 0.011 |  | -0.632 | 0.276 | 0.022 |
| rs116587722-1 | -0.009 | 0.059 | 0.883 |  | -0.044 | 0.059 | 0.461 |  | -0.056 | 0.059 | 0.343 |
| rs11658168-2 | 0.035 | 0.054 | 0.515 |  | -0.004 | 0.054 | 0.944 |  | -0.044 | 0.053 | 0.415 |
| rs11658168-1 | -0.031 | 0.037 | 0.405 |  | -0.036 | 0.037 | 0.329 |  | -0.037 | 0.037 | 0.313 |
| rs11657987-2 | -0.028 | 0.049 | 0.572 |  | 0.010 | 0.049 | 0.846 |  | 0.016 | 0.049 | 0.741 |
| rs11657987-1 | 0.027 | 0.041 | 0.508 |  | 0.026 | 0.042 | 0.525 |  | -0.014 | 0.041 | 0.744 |
| rs116504838-2 | 0.878 | 0.487 | 0.072 |  | 0.581 | 0.489 | 0.235 |  | 1.199 | 0.486 | 0.014 |
| rs116504838-1 | 0.903 | 0.494 | 0.067 |  | 0.596 | 0.496 | 0.230 |  | 1.265 | 0.492 | 0.010 |
| rs11643303-2 | 0.060 | 0.114 | 0.600 |  | -0.002 | 0.114 | 0.985 |  | 0.277 | 0.113 | 0.014 |
| rs11643303-1 | 0.092 | 0.115 | 0.424 |  | -0.004 | 0.115 | 0.970 |  | 0.232 | 0.114 | 0.042 |
| rs11643240-2 | -0.083 | 0.063 | 0.191 |  | -0.064 | 0.064 | 0.317 |  | -0.086 | 0.063 | 0.176 |
| rs11643240-1 | -0.036 | 0.037 | 0.334 |  | -0.039 | 0.037 | 0.286 |  | -0.058 | 0.037 | 0.115 |
| rs11631075-2 | 0.023 | 0.049 | 0.632 |  | 0.032 | 0.049 | 0.515 |  | -0.072 | 0.049 | 0.140 |
| rs11631075-1 | 0.021 | 0.044 | 0.642 |  | -0.009 | 0.045 | 0.838 |  | -0.028 | 0.044 | 0.524 |
| rs116228246-2 | 0.140 | 0.684 | 0.838 |  | 0.135 | 0.687 | 0.844 |  | 0.018 | 0.682 | 0.978 |
| rs116228246-1 | 0.101 | 0.689 | 0.884 |  | 0.138 | 0.692 | 0.842 |  | 0.114 | 0.687 | 0.868 |
| rs116169065-2 | 0.039 | 0.106 | 0.713 |  | 0.080 | 0.107 | 0.454 |  | 0.088 | 0.106 | 0.407 |
| rs116169065-1 | 0.013 | 0.039 | 0.734 |  | 0.028 | 0.039 | 0.473 |  | -0.015 | 0.039 | 0.698 |
| rs11615765-2 | 0.219 | 0.154 | 0.155 |  | 0.114 | 0.155 | 0.462 |  | -0.045 | 0.153 | 0.770 |
| rs11615765-1 | 0.013 | 0.043 | 0.766 |  | 0.009 | 0.043 | 0.833 |  | 0.017 | 0.043 | 0.700 |
| rs11601792-2 | -0.200 | 0.183 | 0.274 |  | -0.160 | 0.183 | 0.382 |  | -0.101 | 0.184 | 0.582 |
| rs11601792-1 | 0.069 | 0.049 | 0.156 |  | 0.092 | 0.049 | 0.059 |  | 0.032 | 0.049 | 0.508 |
| rs1159798-2 | -0.014 | 0.075 | 0.854 |  | -0.011 | 0.076 | 0.883 |  | 0.050 | 0.075 | 0.502 |
| rs1159798-1 | -0.027 | 0.074 | 0.713 |  | -0.006 | 0.074 | 0.935 |  | -0.002 | 0.074 | 0.980 |
| rs11587434-2 | -0.027 | 0.066 | 0.679 |  | -0.086 | 0.066 | 0.195 |  | 0.064 | 0.066 | 0.330 |
| rs11587434-1 | 0.074 | 0.068 | 0.281 |  | -0.010 | 0.069 | 0.889 |  | 0.023 | 0.068 | 0.737 |
| rs11584380-2 | 0.007 | 0.049 | 0.888 |  | 0.031 | 0.050 | 0.527 |  | 0.002 | 0.049 | 0.965 |
| rs11584380-1 | -0.005 | 0.047 | 0.915 |  | -0.009 | 0.047 | 0.842 |  | 0.013 | 0.047 | 0.784 |
| rs115814778-1 | 0.056 | 0.124 | 0.651 |  | 0.064 | 0.124 | 0.608 |  | -0.062 | 0.123 | 0.613 |
| rs11576308-2 | 0.006 | 0.052 | 0.904 |  | -0.011 | 0.053 | 0.838 |  | 0.006 | 0.052 | 0.913 |
| rs11576308-1 | -0.036 | 0.038 | 0.338 |  | -0.034 | 0.038 | 0.371 |  | -0.023 | 0.037 | 0.541 |
| rs115242848-2 | -0.301 | 0.162 | 0.064 |  | -0.179 | 0.163 | 0.271 |  | -0.257 | 0.162 | 0.114 |
| rs1151490-2 | -0.075 | 0.052 | 0.147 |  | -0.053 | 0.052 | 0.303 |  | -0.081 | 0.051 | 0.113 |
| rs1151490-1 | -0.106 | 0.046 | 0.020 |  | -0.108 | 0.046 | 0.019 |  | -0.080 | 0.046 | 0.081 |
| rs114621605-2 | -1.505 | 0.893 | 0.092 |  | -0.350 | 0.897 | 0.696 |  | -1.872 | 0.891 | 0.036 |
| rs114621605-1 | -0.136 | 0.100 | 0.176 |  | -0.123 | 0.101 | 0.222 |  | -0.034 | 0.100 | 0.734 |
| rs1145656-2 | -0.098 | 0.086 | 0.254 |  | -0.149 | 0.086 | 0.084 |  | -0.023 | 0.085 | 0.784 |
| rs1145656-1 | 0.076 | 0.038 | 0.045 |  | 0.084 | 0.038 | 0.027 |  | 0.053 | 0.038 | 0.160 |
| rs114124763-2 | 0.505 | 0.414 | 0.223 |  | 0.386 | 0.416 | 0.353 |  | -0.219 | 0.413 | 0.595 |
| rs114124763-1 | 0.531 | 0.416 | 0.202 |  | 0.390 | 0.418 | 0.351 |  | -0.333 | 0.415 | 0.422 |
| rs113896694-2 | 0.005 | 0.132 | 0.970 |  | -0.050 | 0.133 | 0.704 |  | -0.081 | 0.132 | 0.539 |
| rs113896694-1 | 0.002 | 0.041 | 0.965 |  | -0.001 | 0.042 | 0.979 |  | -0.054 | 0.041 | 0.192 |
| rs113880644-2 | 0.139 | 0.258 | 0.589 |  | -0.056 | 0.259 | 0.829 |  | -0.176 | 0.257 | 0.494 |
| rs113880644-1 | -0.022 | 0.055 | 0.695 |  | -0.009 | 0.055 | 0.872 |  | 0.022 | 0.055 | 0.686 |
| rs113824873-1 | 0.094 | 0.141 | 0.506 |  | 0.019 | 0.142 | 0.892 |  | -0.248 | 0.141 | 0.078 |
| rs113821260-2 | 0.458 | 0.272 | 0.093 |  | 0.367 | 0.274 | 0.181 |  | 0.027 | 0.272 | 0.920 |
| rs113821260-1 | 0.474 | 0.274 | 0.084 |  | 0.365 | 0.276 | 0.185 |  | 0.034 | 0.274 | 0.901 |
| rs113773470-2 | -0.105 | 0.117 | 0.371 |  | -0.055 | 0.118 | 0.639 |  | -0.003 | 0.117 | 0.980 |
| rs113773470-1 | -0.051 | 0.120 | 0.673 |  | -0.032 | 0.121 | 0.790 |  | 0.007 | 0.120 | 0.951 |
| rs113557605-2 | -0.037 | 0.214 | 0.862 |  | -0.061 | 0.215 | 0.777 |  | 0.088 | 0.213 | 0.680 |
| rs113557605-1 | -0.072 | 0.050 | 0.153 |  | -0.062 | 0.051 | 0.220 |  | -0.034 | 0.050 | 0.504 |
| rs113477878-2 | 0.060 | 0.334 | 0.858 |  | 0.142 | 0.336 | 0.673 |  | -0.349 | 0.333 | 0.295 |
| rs113477878-1 | 0.050 | 0.058 | 0.381 |  | 0.030 | 0.058 | 0.610 |  | 0.043 | 0.058 | 0.458 |
| rs1133400-2 | -0.060 | 0.082 | 0.468 |  | -0.055 | 0.083 | 0.508 |  | -0.136 | 0.082 | 0.099 |
| rs1133400-1 | -0.059 | 0.085 | 0.489 |  | -0.048 | 0.085 | 0.575 |  | -0.138 | 0.084 | 0.103 |
| rs113150176-2 | 0.506 | 0.542 | 0.351 |  | 0.148 | 0.544 | 0.786 |  | -0.051 | 0.540 | 0.924 |
| rs113150176-1 | 0.336 | 0.547 | 0.539 |  | 0.004 | 0.550 | 0.995 |  | -0.083 | 0.545 | 0.879 |
| rs112900993-2 | 0.017 | 0.070 | 0.807 |  | 0.013 | 0.070 | 0.854 |  | 0.008 | 0.069 | 0.912 |
| rs112900993-1 | 0.020 | 0.071 | 0.778 |  | -0.006 | 0.071 | 0.937 |  | -0.017 | 0.070 | 0.814 |
| rs112766772-2 | -0.019 | 0.084 | 0.823 |  | 0.018 | 0.084 | 0.831 |  | -0.071 | 0.084 | 0.397 |
| rs112766772-1 | 0.019 | 0.086 | 0.822 |  | 0.060 | 0.086 | 0.484 |  | -0.045 | 0.086 | 0.601 |
| rs112745054-2 | -0.082 | 0.458 | 0.857 |  | -0.079 | 0.460 | 0.863 |  | -0.628 | 0.456 | 0.169 |
| rs112745054-1 | 0.027 | 0.070 | 0.697 |  | 0.018 | 0.070 | 0.797 |  | -0.040 | 0.070 | 0.567 |
| rs11259979-2 | -0.057 | 0.064 | 0.375 |  | -0.049 | 0.064 | 0.444 |  | -0.034 | 0.064 | 0.596 |
| rs11259979-1 | -0.073 | 0.036 | 0.043 |  | -0.058 | 0.036 | 0.110 |  | -0.054 | 0.036 | 0.133 |
| rs11245388-2 | 0.016 | 0.050 | 0.751 |  | 0.013 | 0.050 | 0.794 |  | -0.038 | 0.050 | 0.453 |
| rs11245388-1 | -0.032 | 0.040 | 0.412 |  | -0.053 | 0.040 | 0.186 |  | -0.038 | 0.039 | 0.341 |
| rs11242735-2 | 0.063 | 0.073 | 0.385 |  | 0.050 | 0.073 | 0.494 |  | -0.033 | 0.072 | 0.645 |
| rs11242735-1 | 0.023 | 0.074 | 0.758 |  | -0.008 | 0.075 | 0.920 |  | 0.036 | 0.074 | 0.625 |
| rs11238756-2 | 0.036 | 0.049 | 0.457 |  | 0.035 | 0.049 | 0.473 |  | 0.054 | 0.048 | 0.262 |
| rs11238756-1 | 0.010 | 0.044 | 0.812 |  | -0.010 | 0.044 | 0.821 |  | 0.051 | 0.044 | 0.243 |
| rs11238526-2 | -0.326 | 0.183 | 0.075 |  | -0.345 | 0.184 | 0.061 |  | -0.330 | 0.183 | 0.071 |
| rs11238526-1 | -0.251 | 0.185 | 0.174 |  | -0.260 | 0.186 | 0.162 |  | -0.221 | 0.185 | 0.232 |
| rs11231740-2 | 0.047 | 0.049 | 0.340 |  | 0.083 | 0.050 | 0.094 |  | -0.038 | 0.049 | 0.438 |
| rs11231740-1 | -0.018 | 0.039 | 0.652 |  | -0.022 | 0.040 | 0.574 |  | -0.050 | 0.039 | 0.200 |
| rs1123015-2 | 0.045 | 0.055 | 0.405 |  | 0.103 | 0.055 | 0.061 |  | 0.020 | 0.054 | 0.719 |
| rs1123015-1 | -0.116 | 0.037 | 0.002 |  | -0.058 | 0.037 | 0.120 |  | -0.028 | 0.037 | 0.453 |
| rs11228240-2 | -0.006 | 0.075 | 0.935 |  | 0.063 | 0.075 | 0.402 |  | -0.036 | 0.075 | 0.628 |
| rs11228240-1 | 0.018 | 0.038 | 0.633 |  | 0.057 | 0.038 | 0.130 |  | -0.042 | 0.038 | 0.268 |
| rs112187554-2 | -1.388 | 0.852 | 0.104 |  | -0.797 | 0.856 | 0.352 |  | -1.144 | 0.850 | 0.178 |
| rs112187554-1 | -1.394 | 0.856 | 0.104 |  | -0.795 | 0.860 | 0.355 |  | -1.192 | 0.853 | 0.163 |
| rs112073168-2 | -0.306 | 0.487 | 0.530 |  | -0.325 | 0.490 | 0.506 |  | -0.798 | 0.486 | 0.101 |
| rs112073168-1 | 0.052 | 0.077 | 0.500 |  | 0.037 | 0.077 | 0.628 |  | -0.042 | 0.077 | 0.585 |
| rs11196170-2 | -0.102 | 0.075 | 0.176 |  | -0.056 | 0.075 | 0.455 |  | -0.049 | 0.075 | 0.510 |
| rs11196170-1 | -0.032 | 0.037 | 0.379 |  | 0.003 | 0.037 | 0.945 |  | 0.028 | 0.037 | 0.451 |
| rs11195154-2 | -0.014 | 0.110 | 0.895 |  | -0.079 | 0.110 | 0.476 |  | 0.046 | 0.110 | 0.672 |
| rs11195154-1 | 0.034 | 0.113 | 0.764 |  | -0.026 | 0.113 | 0.816 |  | 0.145 | 0.113 | 0.199 |
| rs11191614-2 | 0.055 | 0.125 | 0.662 |  | 0.071 | 0.126 | 0.572 |  | 0.016 | 0.125 | 0.901 |
| rs11191614-1 | -0.039 | 0.041 | 0.339 |  | -0.038 | 0.041 | 0.360 |  | -0.013 | 0.041 | 0.742 |
| rs11185677-2 | -0.162 | 0.097 | 0.096 |  | -0.214 | 0.098 | 0.029 |  | -0.030 | 0.097 | 0.753 |
| rs11185677-1 | 0.046 | 0.039 | 0.234 |  | 0.034 | 0.039 | 0.379 |  | 0.076 | 0.039 | 0.048 |
| rs111785286-2 | -0.522 | 0.499 | 0.296 |  | -0.440 | 0.502 | 0.380 |  | -0.146 | 0.498 | 0.769 |
| rs111785286-1 | -0.494 | 0.501 | 0.325 |  | -0.375 | 0.504 | 0.457 |  | -0.082 | 0.500 | 0.870 |
| rs11175835-2 | 0.006 | 0.062 | 0.921 |  | 0.016 | 0.063 | 0.793 |  | -0.066 | 0.062 | 0.287 |
| rs11175835-1 | 0.032 | 0.061 | 0.606 |  | 0.004 | 0.062 | 0.947 |  | 0.047 | 0.061 | 0.444 |
| rs111632154-2 | -1.154 | 0.865 | 0.182 |  | -0.821 | 0.868 | 0.345 |  | 0.208 | 0.862 | 0.810 |
| rs111632154-1 | -0.174 | 0.070 | 0.013 |  | -0.164 | 0.071 | 0.021 |  | -0.127 | 0.070 | 0.070 |
| rs111528363-2 | -2.107 | 0.854 | 0.014 |  | -1.549 | 0.858 | 0.071 |  | -1.519 | 0.851 | 0.074 |
| rs111528363-1 | -0.018 | 0.100 | 0.855 |  | -0.091 | 0.100 | 0.365 |  | -0.139 | 0.099 | 0.160 |
| rs11142400-2 | -0.003 | 0.056 | 0.957 |  | -0.014 | 0.056 | 0.805 |  | -0.003 | 0.056 | 0.959 |
| rs11142400-1 | -0.064 | 0.056 | 0.252 |  | -0.075 | 0.057 | 0.187 |  | -0.114 | 0.056 | 0.043 |
| rs1111258-2 | -0.038 | 0.051 | 0.456 |  | -0.075 | 0.051 | 0.145 |  | -0.014 | 0.051 | 0.782 |
| rs1111258-1 | -0.021 | 0.038 | 0.575 |  | -0.030 | 0.038 | 0.433 |  | -0.017 | 0.038 | 0.642 |
| rs1110494-2 | -0.073 | 0.058 | 0.208 |  | -0.120 | 0.058 | 0.040 |  | -0.041 | 0.058 | 0.474 |
| rs1110494-1 | -0.046 | 0.036 | 0.204 |  | -0.050 | 0.036 | 0.167 |  | -0.024 | 0.036 | 0.505 |
| rs11088458-2 | -0.143 | 0.062 | 0.023 |  | -0.133 | 0.063 | 0.034 |  | -0.184 | 0.062 | 0.003 |
| rs11088458-1 | -0.133 | 0.062 | 0.032 |  | -0.162 | 0.062 | 0.010 |  | -0.143 | 0.062 | 0.021 |
| rs11084689-2 | -0.140 | 0.073 | 0.056 |  | -0.198 | 0.074 | 0.007 |  | -0.059 | 0.073 | 0.418 |
| rs11084689-1 | -0.181 | 0.075 | 0.016 |  | -0.224 | 0.075 | 0.003 |  | -0.086 | 0.074 | 0.247 |
| rs11079166-2 | 0.053 | 0.051 | 0.300 |  | 0.056 | 0.052 | 0.279 |  | 0.039 | 0.051 | 0.442 |
| rs11079166-1 | 0.045 | 0.049 | 0.366 |  | 0.019 | 0.049 | 0.699 |  | 0.034 | 0.049 | 0.491 |
| rs11078776-2 | -0.002 | 0.049 | 0.970 |  | -0.006 | 0.049 | 0.898 |  | 0.082 | 0.049 | 0.091 |
| rs11078776-1 | -0.044 | 0.045 | 0.335 |  | -0.033 | 0.046 | 0.470 |  | -0.010 | 0.045 | 0.828 |
| rs11073930-2 | -0.031 | 0.048 | 0.522 |  | -0.018 | 0.048 | 0.704 |  | -0.009 | 0.048 | 0.849 |
| rs11073930-1 | -0.055 | 0.041 | 0.180 |  | -0.058 | 0.041 | 0.153 |  | -0.012 | 0.041 | 0.771 |
| rs11067228-2 | 0.032 | 0.051 | 0.524 |  | 0.026 | 0.051 | 0.612 |  | 0.110 | 0.050 | 0.030 |
| rs11067228-1 | 0.033 | 0.046 | 0.477 |  | 0.020 | 0.046 | 0.661 |  | 0.035 | 0.046 | 0.449 |
| rs11061814-2 | -0.064 | 0.123 | 0.601 |  | -0.104 | 0.123 | 0.400 |  | -0.094 | 0.122 | 0.443 |
| rs11061814-1 | 0.000 | 0.041 | 0.999 |  | 0.024 | 0.041 | 0.555 |  | 0.016 | 0.041 | 0.692 |
| rs11049943-2 | 0.073 | 0.106 | 0.490 |  | 0.102 | 0.107 | 0.341 |  | 0.023 | 0.106 | 0.832 |
| rs11049943-1 | 0.037 | 0.040 | 0.354 |  | 0.052 | 0.040 | 0.199 |  | -0.014 | 0.040 | 0.734 |
| rs11023759-2 | -0.375 | 0.124 | 0.002 |  | -0.420 | 0.124 | 0.001 |  | -0.328 | 0.123 | 0.008 |
| rs11023759-1 | -0.124 | 0.041 | 0.002 |  | -0.106 | 0.041 | 0.009 |  | -0.086 | 0.040 | 0.033 |
| rs11023722-2 | 0.154 | 0.329 | 0.641 |  | 0.190 | 0.331 | 0.566 |  | 0.224 | 0.342 | 0.513 |
| rs11023722-1 | 0.287 | 0.333 | 0.389 |  | 0.266 | 0.334 | 0.426 |  | 0.309 | 0.345 | 0.370 |
| rs10992489-2 | 0.017 | 0.055 | 0.750 |  | 0.037 | 0.055 | 0.502 |  | 0.019 | 0.055 | 0.727 |
| rs10992489-1 | 0.044 | 0.037 | 0.231 |  | 0.051 | 0.037 | 0.170 |  | 0.053 | 0.037 | 0.149 |
| rs10980517-2 | -0.024 | 0.049 | 0.622 |  | -0.003 | 0.050 | 0.951 |  | -0.027 | 0.049 | 0.580 |
| rs10980517-1 | -0.025 | 0.043 | 0.558 |  | 0.007 | 0.043 | 0.863 |  | -0.019 | 0.043 | 0.662 |
| rs10979330-2 | 0.123 | 0.076 | 0.104 |  | 0.210 | 0.076 | 0.006 |  | 0.059 | 0.076 | 0.438 |
| rs10979330-1 | 0.117 | 0.076 | 0.123 |  | 0.218 | 0.076 | 0.004 |  | 0.027 | 0.076 | 0.726 |
| rs10956974-2 | -0.030 | 0.078 | 0.699 |  | 0.023 | 0.078 | 0.773 |  | -0.005 | 0.078 | 0.950 |
| rs10956974-1 | -0.018 | 0.080 | 0.825 |  | 0.027 | 0.080 | 0.731 |  | -0.014 | 0.079 | 0.858 |
| rs10948100-2 | -0.012 | 0.055 | 0.821 |  | 0.036 | 0.055 | 0.517 |  | -0.039 | 0.054 | 0.469 |
| rs10948100-1 | 0.005 | 0.036 | 0.899 |  | 0.040 | 0.036 | 0.267 |  | -0.006 | 0.036 | 0.863 |
| rs10941433-2 | -0.250 | 0.140 | 0.074 |  | -0.226 | 0.140 | 0.108 |  | -0.249 | 0.139 | 0.073 |
| rs10941433-1 | -0.137 | 0.042 | 0.001 |  | -0.093 | 0.043 | 0.029 |  | -0.084 | 0.042 | 0.047 |
| rs10931982-2 | 0.059 | 0.087 | 0.497 |  | 0.085 | 0.087 | 0.330 |  | 0.020 | 0.087 | 0.814 |
| rs10931982-1 | -0.055 | 0.037 | 0.141 |  | -0.067 | 0.037 | 0.074 |  | -0.023 | 0.037 | 0.538 |
| rs10920352-2 | 0.012 | 0.049 | 0.807 |  | -0.009 | 0.050 | 0.858 |  | -0.017 | 0.049 | 0.727 |
| rs10920352-1 | 0.011 | 0.046 | 0.814 |  | -0.013 | 0.046 | 0.781 |  | -0.018 | 0.046 | 0.689 |
| rs10917477-2 | 0.002 | 0.048 | 0.969 |  | 0.023 | 0.048 | 0.625 |  | -0.023 | 0.047 | 0.630 |
| rs10917477-1 | -0.020 | 0.043 | 0.635 |  | -0.023 | 0.043 | 0.598 |  | -0.003 | 0.043 | 0.944 |
| rs10893348-2 | 0.007 | 0.050 | 0.884 |  | -0.016 | 0.051 | 0.748 |  | 0.024 | 0.050 | 0.630 |
| rs10893348-1 | 0.014 | 0.047 | 0.768 |  | 0.010 | 0.047 | 0.839 |  | 0.008 | 0.047 | 0.857 |
| rs10887745-2 | 0.016 | 0.051 | 0.746 |  | -0.013 | 0.051 | 0.794 |  | 0.058 | 0.050 | 0.247 |
| rs10887745-1 | -0.052 | 0.039 | 0.179 |  | -0.067 | 0.039 | 0.082 |  | 0.034 | 0.038 | 0.378 |
| rs10885434-2 | 0.078 | 0.062 | 0.211 |  | 0.004 | 0.062 | 0.943 |  | 0.077 | 0.062 | 0.212 |
| rs10885434-1 | 0.099 | 0.061 | 0.106 |  | 0.002 | 0.062 | 0.975 |  | 0.093 | 0.061 | 0.129 |
| rs10878984-2 | -0.022 | 0.056 | 0.699 |  | -0.088 | 0.056 | 0.117 |  | 0.028 | 0.056 | 0.617 |
| rs10878984-1 | 0.051 | 0.037 | 0.172 |  | 0.000 | 0.037 | 0.998 |  | -0.009 | 0.037 | 0.799 |
| rs10875906-2 | -0.031 | 0.064 | 0.624 |  | -0.080 | 0.065 | 0.218 |  | -0.047 | 0.064 | 0.466 |
| rs10875906-1 | -0.009 | 0.064 | 0.890 |  | -0.057 | 0.064 | 0.376 |  | -0.007 | 0.064 | 0.912 |
| rs10859140-2 | -0.090 | 0.051 | 0.079 |  | -0.035 | 0.052 | 0.500 |  | 0.011 | 0.051 | 0.829 |
| rs10859140-1 | -0.065 | 0.038 | 0.091 |  | -0.050 | 0.038 | 0.193 |  | -0.021 | 0.038 | 0.578 |
| rs10852610-2 | -0.105 | 0.053 | 0.049 |  | -0.071 | 0.053 | 0.182 |  | -0.097 | 0.053 | 0.068 |
| rs10852610-1 | -0.089 | 0.043 | 0.037 |  | -0.081 | 0.043 | 0.060 |  | -0.098 | 0.043 | 0.022 |
| rs10849492-2 | 0.023 | 0.090 | 0.798 |  | 0.107 | 0.090 | 0.237 |  | -0.034 | 0.090 | 0.708 |
| rs10849492-1 | 0.038 | 0.092 | 0.678 |  | 0.136 | 0.092 | 0.140 |  | 0.004 | 0.092 | 0.966 |
| rs10842704-2 | -0.190 | 0.071 | 0.008 |  | -0.114 | 0.072 | 0.112 |  | -0.054 | 0.071 | 0.452 |
| rs10842704-1 | -0.205 | 0.072 | 0.004 |  | -0.146 | 0.072 | 0.043 |  | -0.039 | 0.071 | 0.589 |
| rs10835483-2 | -0.290 | 0.116 | 0.012 |  | -0.215 | 0.116 | 0.064 |  | -0.185 | 0.116 | 0.109 |
| rs10835483-1 | -0.077 | 0.040 | 0.053 |  | -0.083 | 0.040 | 0.038 |  | -0.060 | 0.040 | 0.128 |
| rs10835169-2 | -0.079 | 0.061 | 0.195 |  | -0.062 | 0.061 | 0.309 |  | -0.049 | 0.061 | 0.422 |
| rs10835169-1 | -0.041 | 0.058 | 0.486 |  | -0.001 | 0.058 | 0.985 |  | -0.022 | 0.058 | 0.709 |
| rs10817896-2 | -0.129 | 0.066 | 0.050 |  | -0.121 | 0.066 | 0.068 |  | -0.108 | 0.066 | 0.100 |
| rs10817896-1 | -0.154 | 0.067 | 0.021 |  | -0.117 | 0.067 | 0.079 |  | -0.149 | 0.066 | 0.025 |
| rs1080789-2 | -0.077 | 0.049 | 0.115 |  | -0.058 | 0.049 | 0.238 |  | -0.018 | 0.049 | 0.717 |
| rs1080789-1 | -0.003 | 0.040 | 0.931 |  | -0.028 | 0.040 | 0.483 |  | 0.041 | 0.040 | 0.303 |
| rs10805874-2 | -0.019 | 0.061 | 0.758 |  | -0.010 | 0.061 | 0.866 |  | -0.094 | 0.060 | 0.121 |
| rs10805874-1 | 0.016 | 0.061 | 0.794 |  | 0.008 | 0.061 | 0.901 |  | -0.050 | 0.061 | 0.412 |
| rs10800531-2 | -0.001 | 0.048 | 0.976 |  | 0.003 | 0.049 | 0.956 |  | 0.016 | 0.048 | 0.745 |
| rs10800531-1 | 0.002 | 0.042 | 0.954 |  | -0.005 | 0.043 | 0.912 |  | -0.023 | 0.042 | 0.593 |
| rs10792352-2 | -0.037 | 0.061 | 0.548 |  | 0.021 | 0.061 | 0.738 |  | 0.031 | 0.061 | 0.611 |
| rs10792352-1 | 0.004 | 0.036 | 0.903 |  | -0.003 | 0.036 | 0.937 |  | 0.013 | 0.036 | 0.723 |
| rs10790255-2 | -0.107 | 0.075 | 0.155 |  | -0.073 | 0.075 | 0.334 |  | -0.148 | 0.075 | 0.048 |
| rs10790255-1 | -0.017 | 0.036 | 0.630 |  | -0.015 | 0.036 | 0.678 |  | -0.029 | 0.035 | 0.421 |
| rs10783573-2 | 0.024 | 0.055 | 0.663 |  | 0.007 | 0.056 | 0.896 |  | -0.115 | 0.055 | 0.038 |
| rs10783573-1 | 0.058 | 0.056 | 0.293 |  | 0.028 | 0.056 | 0.617 |  | -0.109 | 0.055 | 0.049 |
| rs10779795-2 | -0.025 | 0.055 | 0.648 |  | -0.030 | 0.055 | 0.587 |  | 0.023 | 0.055 | 0.677 |
| rs10779795-1 | 0.005 | 0.036 | 0.887 |  | 0.011 | 0.036 | 0.762 |  | -0.027 | 0.036 | 0.446 |
| rs10777536-2 | 0.076 | 0.049 | 0.122 |  | 0.080 | 0.049 | 0.106 |  | 0.075 | 0.049 | 0.126 |
| rs10777536-1 | 0.019 | 0.042 | 0.646 |  | -0.002 | 0.042 | 0.955 |  | 0.040 | 0.042 | 0.339 |
| rs10765568-2 | -0.038 | 0.055 | 0.484 |  | -0.060 | 0.055 | 0.273 |  | -0.048 | 0.054 | 0.381 |
| rs10765568-1 | -0.002 | 0.037 | 0.955 |  | -0.022 | 0.037 | 0.565 |  | -0.077 | 0.037 | 0.039 |
| rs10764201-2 | -0.055 | 0.049 | 0.258 |  | -0.068 | 0.049 | 0.166 |  | -0.042 | 0.049 | 0.384 |
| rs10764201-1 | -0.080 | 0.041 | 0.051 |  | -0.062 | 0.041 | 0.129 |  | -0.030 | 0.041 | 0.458 |
| rs10756762-2 | -0.085 | 0.054 | 0.115 |  | -0.086 | 0.054 | 0.114 |  | -0.059 | 0.054 | 0.276 |
| rs10756762-1 | -0.052 | 0.052 | 0.320 |  | -0.064 | 0.052 | 0.219 |  | -0.041 | 0.052 | 0.435 |
| rs10750766-2 | -0.067 | 0.062 | 0.282 |  | -0.064 | 0.063 | 0.310 |  | -0.039 | 0.062 | 0.526 |
| rs10750766-1 | 0.010 | 0.037 | 0.781 |  | 0.027 | 0.037 | 0.473 |  | -0.042 | 0.037 | 0.257 |
| rs10749436-2 | -0.053 | 0.073 | 0.467 |  | -0.002 | 0.073 | 0.975 |  | -0.006 | 0.073 | 0.938 |
| rs10749436-1 | -0.086 | 0.074 | 0.241 |  | -0.067 | 0.074 | 0.367 |  | -0.070 | 0.074 | 0.339 |
| rs10746495-2 | 0.096 | 0.088 | 0.274 |  | 0.162 | 0.088 | 0.066 |  | 0.032 | 0.087 | 0.711 |
| rs10746495-1 | 0.050 | 0.089 | 0.575 |  | 0.134 | 0.090 | 0.134 |  | 0.006 | 0.089 | 0.948 |
| rs10744592-2 | 0.026 | 0.050 | 0.609 |  | 0.042 | 0.050 | 0.402 |  | -0.037 | 0.050 | 0.462 |
| rs10744592-1 | 0.003 | 0.038 | 0.935 |  | -0.012 | 0.038 | 0.763 |  | -0.004 | 0.038 | 0.916 |
| rs10741695-2 | -0.083 | 0.086 | 0.338 |  | -0.137 | 0.087 | 0.115 |  | -0.064 | 0.086 | 0.457 |
| rs10741695-1 | 0.047 | 0.089 | 0.597 |  | -0.024 | 0.089 | 0.790 |  | 0.050 | 0.088 | 0.570 |
| rs10740059-2 | -0.014 | 0.048 | 0.765 |  | -0.015 | 0.048 | 0.759 |  | 0.042 | 0.048 | 0.385 |
| rs10740059-1 | 0.000 | 0.046 | 0.998 |  | -0.034 | 0.046 | 0.454 |  | -0.004 | 0.046 | 0.929 |
| rs10732635-2 | -0.110 | 0.049 | 0.025 |  | -0.116 | 0.049 | 0.019 |  | -0.106 | 0.049 | 0.030 |
| rs10732635-1 | -0.039 | 0.039 | 0.315 |  | -0.033 | 0.039 | 0.402 |  | -0.100 | 0.039 | 0.010 |
| rs10515269-2 | -0.076 | 0.048 | 0.114 |  | -0.035 | 0.048 | 0.470 |  | -0.055 | 0.048 | 0.249 |
| rs10515269-1 | -0.041 | 0.041 | 0.319 |  | -0.038 | 0.041 | 0.355 |  | -0.058 | 0.041 | 0.154 |
| rs10505116-2 | -0.048 | 0.158 | 0.760 |  | -0.137 | 0.159 | 0.389 |  | -0.165 | 0.158 | 0.295 |
| rs10505116-1 | -0.020 | 0.044 | 0.655 |  | 0.004 | 0.044 | 0.925 |  | -0.014 | 0.044 | 0.755 |
| rs10499878-2 | 0.023 | 0.091 | 0.803 |  | 0.009 | 0.092 | 0.922 |  | 0.011 | 0.091 | 0.906 |
| rs10499878-1 | 0.041 | 0.095 | 0.665 |  | 0.029 | 0.095 | 0.757 |  | 0.040 | 0.094 | 0.671 |
| rs10493130-2 | 0.069 | 0.059 | 0.248 |  | 0.071 | 0.060 | 0.230 |  | -0.039 | 0.059 | 0.515 |
| rs10493130-1 | 0.002 | 0.060 | 0.970 |  | 0.006 | 0.061 | 0.919 |  | -0.037 | 0.060 | 0.538 |
| rs10490046-2 | -0.076 | 0.076 | 0.319 |  | -0.053 | 0.076 | 0.485 |  | 0.017 | 0.076 | 0.819 |
| rs10490046-1 | -0.042 | 0.037 | 0.260 |  | -0.017 | 0.038 | 0.655 |  | -0.014 | 0.037 | 0.706 |
| rs1048932-2 | -0.080 | 0.050 | 0.110 |  | -0.049 | 0.050 | 0.326 |  | -0.001 | 0.050 | 0.990 |
| rs1048932-1 | -0.073 | 0.047 | 0.119 |  | -0.042 | 0.047 | 0.370 |  | -0.032 | 0.047 | 0.495 |
| rs10481112-2 | -0.030 | 0.059 | 0.609 |  | -0.084 | 0.059 | 0.157 |  | 0.014 | 0.059 | 0.809 |
| rs10481112-1 | 0.022 | 0.036 | 0.554 |  | 0.022 | 0.037 | 0.545 |  | 0.041 | 0.036 | 0.260 |
| rs10473868-2 | 0.033 | 0.048 | 0.493 |  | -0.013 | 0.049 | 0.788 |  | 0.050 | 0.048 | 0.303 |
| rs10473868-1 | 0.003 | 0.040 | 0.932 |  | -0.048 | 0.041 | 0.239 |  | 0.029 | 0.040 | 0.468 |
| rs10462395-2 | 0.043 | 0.085 | 0.610 |  | 0.035 | 0.085 | 0.678 |  | 0.042 | 0.084 | 0.621 |
| rs10462395-1 | 0.062 | 0.087 | 0.472 |  | 0.029 | 0.087 | 0.740 |  | 0.015 | 0.086 | 0.866 |
| rs1043003-2 | 0.073 | 0.054 | 0.179 |  | 0.084 | 0.054 | 0.121 |  | 0.006 | 0.054 | 0.904 |
| rs1043003-1 | -0.066 | 0.037 | 0.076 |  | -0.035 | 0.037 | 0.349 |  | -0.045 | 0.037 | 0.218 |
| rs1042704-2 | -0.116 | 0.094 | 0.217 |  | -0.116 | 0.095 | 0.220 |  | -0.125 | 0.094 | 0.184 |
| rs1042704-1 | -0.076 | 0.037 | 0.042 |  | -0.044 | 0.037 | 0.235 |  | -0.081 | 0.037 | 0.030 |
| rs10407062-2 | -0.022 | 0.049 | 0.660 |  | -0.024 | 0.049 | 0.626 |  | -0.030 | 0.049 | 0.533 |
| rs10407062-1 | 0.041 | 0.040 | 0.306 |  | 0.028 | 0.040 | 0.486 |  | 0.036 | 0.040 | 0.364 |
| rs10405373-2 | 0.026 | 0.160 | 0.873 |  | 0.039 | 0.161 | 0.807 |  | 0.055 | 0.160 | 0.730 |
| rs10405373-1 | -0.022 | 0.045 | 0.628 |  | 0.005 | 0.045 | 0.906 |  | -0.057 | 0.045 | 0.204 |
| rs10264106-2 | 0.089 | 0.103 | 0.385 |  | 0.034 | 0.103 | 0.739 |  | -0.263 | 0.102 | 0.010 |
| rs10264106-1 | 0.164 | 0.106 | 0.121 |  | 0.102 | 0.106 | 0.339 |  | -0.174 | 0.105 | 0.099 |
| rs10264053-2 | 0.030 | 0.058 | 0.606 |  | 0.085 | 0.058 | 0.144 |  | -0.089 | 0.058 | 0.121 |
| rs10264053-1 | 0.092 | 0.057 | 0.108 |  | 0.137 | 0.057 | 0.017 |  | -0.049 | 0.057 | 0.387 |
| rs10261246-2 | 0.026 | 0.062 | 0.680 |  | -0.011 | 0.062 | 0.864 |  | 0.065 | 0.062 | 0.296 |
| rs10261246-1 | 0.009 | 0.039 | 0.821 |  | 0.039 | 0.039 | 0.321 |  | -0.006 | 0.039 | 0.875 |
| rs10245353-2 | 0.076 | 0.092 | 0.410 |  | 0.121 | 0.093 | 0.192 |  | -0.043 | 0.092 | 0.640 |
| rs10245353-1 | 0.078 | 0.094 | 0.405 |  | 0.120 | 0.094 | 0.201 |  | -0.049 | 0.093 | 0.603 |
| rs10239787-2 | -0.108 | 0.064 | 0.092 |  | -0.084 | 0.064 | 0.190 |  | -0.084 | 0.064 | 0.186 |
| rs10239787-1 | -0.007 | 0.040 | 0.854 |  | 0.008 | 0.040 | 0.835 |  | -0.014 | 0.040 | 0.725 |
| rs1022463-2 | -0.015 | 0.095 | 0.875 |  | -0.017 | 0.095 | 0.859 |  | -0.057 | 0.094 | 0.543 |
| rs1022463-1 | 0.037 | 0.037 | 0.316 |  | 0.005 | 0.037 | 0.896 |  | -0.032 | 0.037 | 0.384 |
| rs10206992-2 | 0.070 | 0.072 | 0.333 |  | 0.094 | 0.072 | 0.195 |  | 0.032 | 0.072 | 0.654 |
| rs10206992-1 | 0.013 | 0.036 | 0.712 |  | -0.003 | 0.037 | 0.932 |  | -0.004 | 0.036 | 0.914 |
| rs10199437-2 | -0.172 | 0.270 | 0.524 |  | -0.078 | 0.271 | 0.773 |  | -0.306 | 0.269 | 0.256 |
| rs10199437-1 | -0.045 | 0.274 | 0.870 |  | 0.014 | 0.275 | 0.959 |  | -0.283 | 0.273 | 0.300 |
| rs10196726-2 | 0.058 | 0.068 | 0.392 |  | 0.093 | 0.068 | 0.175 |  | -0.005 | 0.068 | 0.939 |
| rs10196726-1 | 0.035 | 0.068 | 0.606 |  | 0.060 | 0.068 | 0.374 |  | -0.017 | 0.068 | 0.796 |
| rs10196674-2 | 0.003 | 0.059 | 0.956 |  | -0.001 | 0.060 | 0.983 |  | -0.048 | 0.059 | 0.422 |
| rs10196674-1 | -0.051 | 0.036 | 0.158 |  | -0.038 | 0.036 | 0.291 |  | -0.013 | 0.036 | 0.710 |
| rs10171206-2 | -0.030 | 0.064 | 0.638 |  | -0.035 | 0.064 | 0.581 |  | -0.090 | 0.064 | 0.156 |
| rs10171206-1 | -0.045 | 0.036 | 0.205 |  | -0.044 | 0.036 | 0.220 |  | -0.131 | 0.036 | 0.000 |
| rs10147522-2 | -0.106 | 0.058 | 0.069 |  | -0.095 | 0.059 | 0.105 |  | -0.089 | 0.058 | 0.126 |
| rs10147522-1 | -0.062 | 0.059 | 0.288 |  | -0.078 | 0.059 | 0.185 |  | -0.039 | 0.058 | 0.502 |
| rs10145299-2 | -0.067 | 0.053 | 0.204 |  | -0.061 | 0.053 | 0.251 |  | -0.061 | 0.053 | 0.247 |
| rs10145299-1 | -0.018 | 0.046 | 0.690 |  | 0.017 | 0.046 | 0.705 |  | -0.053 | 0.046 | 0.249 |
| rs10142836-2 | -0.068 | 0.060 | 0.259 |  | -0.046 | 0.060 | 0.442 |  | -0.028 | 0.060 | 0.636 |
| rs10142836-1 | -0.002 | 0.061 | 0.972 |  | 0.005 | 0.061 | 0.938 |  | -0.003 | 0.060 | 0.954 |
| rs10084164-2 | -0.082 | 0.056 | 0.145 |  | -0.103 | 0.057 | 0.069 |  | -0.016 | 0.056 | 0.773 |
| rs10084164-1 | -0.141 | 0.056 | 0.012 |  | -0.149 | 0.056 | 0.008 |  | -0.084 | 0.056 | 0.131 |
| rs10066412-2 | 0.019 | 0.051 | 0.717 |  | 0.073 | 0.052 | 0.156 |  | 0.005 | 0.051 | 0.927 |
| rs10066412-1 | 0.017 | 0.039 | 0.670 |  | 0.050 | 0.040 | 0.208 |  | -0.006 | 0.039 | 0.887 |
| rs10059225-2 | 0.036 | 0.080 | 0.652 |  | -0.082 | 0.081 | 0.307 |  | -0.101 | 0.080 | 0.209 |
| rs10059225-1 | -0.057 | 0.037 | 0.123 |  | -0.080 | 0.037 | 0.031 |  | -0.050 | 0.037 | 0.172 |
| rs10057855-2 | -0.042 | 0.173 | 0.806 |  | 0.020 | 0.174 | 0.909 |  | -0.224 | 0.172 | 0.194 |
| rs10057855-1 | -0.016 | 0.046 | 0.727 |  | -0.024 | 0.047 | 0.609 |  | -0.001 | 0.046 | 0.985 |
| rs10057211-2 | 0.249 | 0.160 | 0.119 |  | 0.322 | 0.160 | 0.045 |  | -0.033 | 0.159 | 0.834 |
| rs10057211-1 | 0.190 | 0.163 | 0.245 |  | 0.267 | 0.164 | 0.104 |  | -0.068 | 0.163 | 0.675 |
| rs1005502-2 | -0.107 | 0.064 | 0.097 |  | -0.068 | 0.065 | 0.295 |  | -0.081 | 0.064 | 0.209 |
| rs1005502-1 | -0.126 | 0.064 | 0.049 |  | -0.099 | 0.064 | 0.122 |  | -0.092 | 0.064 | 0.149 |
| rs10045962-2 | -0.003 | 0.057 | 0.958 |  | -0.048 | 0.057 | 0.405 |  | 0.000 | 0.057 | 0.998 |
| rs10045962-1 | -0.027 | 0.056 | 0.627 |  | -0.047 | 0.056 | 0.401 |  | -0.032 | 0.056 | 0.571 |
| rs10015974-2 | -0.057 | 0.069 | 0.410 |  | -0.045 | 0.069 | 0.518 |  | -0.023 | 0.069 | 0.738 |
| rs10015974-1 | -0.024 | 0.070 | 0.733 |  | -0.021 | 0.070 | 0.765 |  | -0.046 | 0.070 | 0.507 |
| rs10013456-2 | -0.208 | 0.093 | 0.025 |  | -0.279 | 0.093 | 0.003 |  | -0.249 | 0.092 | 0.007 |
| rs10013456-1 | -0.208 | 0.095 | 0.029 |  | -0.288 | 0.096 | 0.003 |  | -0.241 | 0.095 | 0.011 |

BMD: Bone Mineral Density, SE: Standard Error
